# Structure Sensitivity of CO_2_ Conversion over Nickel Metal Nanoparticles Explained by Micro-Kinetics Simulations

**DOI:** 10.1021/jacsau.2c00430

**Published:** 2022-10-14

**Authors:** Ellen B. Sterk, Anne-Eva Nieuwelink, Matteo Monai, Jaap N. Louwen, Eelco T. C. Vogt, Ivo A. W. Filot, Bert M. Weckhuysen

**Affiliations:** † Inorganic Chemistry and Catalysis Group, Debye Institute for Nanomaterials Science, Utrecht University, Universiteitsweg 99, 3584 CGUtrecht, The Netherlands; ‡ Schuit Institute of Catalysis, Department of Chemical Engineering and Chemistry, Eindhoven University of Technology, P.O. Box 513, 5600 MBEindhoven, The Netherlands

**Keywords:** Sabatier reaction, nickel, carbon dioxide, density functional theory, micro-kinetics simulations

## Abstract

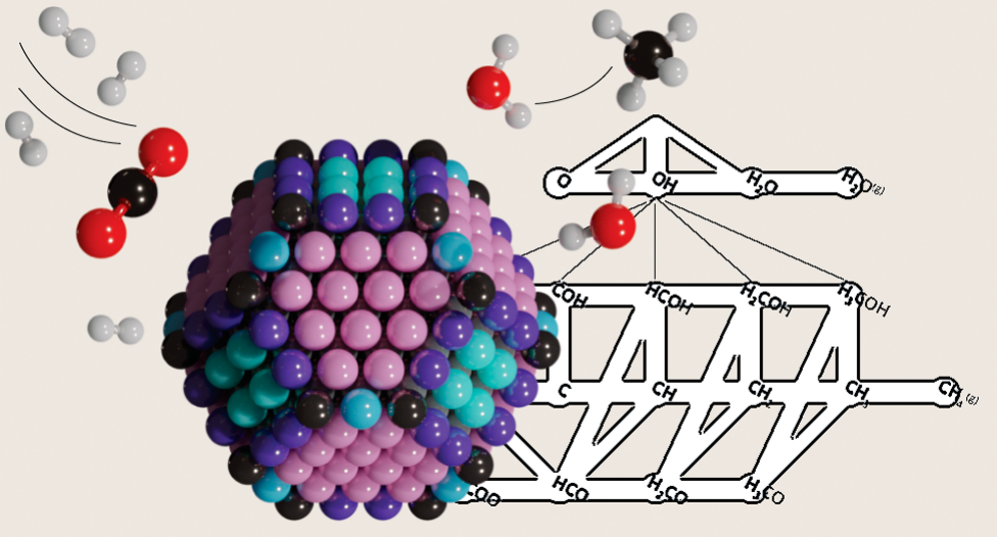

Nickel metal nanoparticles are intensively researched for the catalytic conversion of carbon dioxide. They are commercially explored in the so-called power-to-methane application in which renewably resourced H_2_ reacts with CO_2_ to produce CH_4_, which is better known as the Sabatier reaction. Previous work has shown that this reaction is structure-sensitive. For instance, Ni/SiO_2_ catalysts reveal a maximum performance when nickel metal nanoparticles of ∼2–3 nm are used. Particularly important to a better understanding of the structure sensitivity of the Sabatier reaction over nickel-based catalysts is to understand all relevant elementary reaction steps over various nickel metal facets because this will tell as to which type of nickel facets and which elementary reaction steps are crucial for designing an efficient nickel-based methanation catalyst. In this work, we have determined by density functional theory (DFT) calculations and micro-kinetics modeling (MKM) simulations that the two terrace facets Ni(111) and Ni(100) and the stepped facet Ni(211) barely show any activity in CO_2_ methanation. The stepped facet Ni(110) turned out to be the most effective in CO_2_ methanation. Herein, it was found that the dominant kinetic route corresponds to a combination of the carbide and formate reaction pathways. It was found that the dissociation of H_2_CO* toward CH_2_* and O* is the most critical elementary reaction step on this Ni(110) facet. The calculated activity of a range of Wulff-constructed nickel metal nanoparticles, accounting for varying ratios of the different facets and undercoordinated atoms exposed, reveals the same trend of activity-versus-nanoparticle size, as was observed in previous experimental work from our research group, thereby providing an explanation for the structure-sensitive nature of the Sabatier reaction.

## 1 Introduction

CO_2_ activation and valorization as a low- or even negative-cost feedstock has become a hot topic. During the past century, there has been a significant increase in the CO_2_ level in our atmosphere, which has a cumulative negative effect on our climate. In preceding years, a lot of research has been carried out in the field of catalytic CO_2_ hydrogenation, aiming to find methods to mitigate and valorize CO_2_ emissions. CO_2_ can, for example, be used as a feedstock for the synthesis of platform molecules, such as CO, CH_4_, CH_3_OH, and higher hydrocarbons.^1,2^ From these compounds, we will be able to make many different useful, value-added chemicals, including base chemicals, such as olefins and aromatics.^3,4^ Another interesting approach to valorize CO_2_ is methanation for the chemical storage of electricity via the so-called power-to-gas (P2G) concept, providing routes toward the synthesis of, e.g., methanol and methane.^5,6^

**Figure 1:**
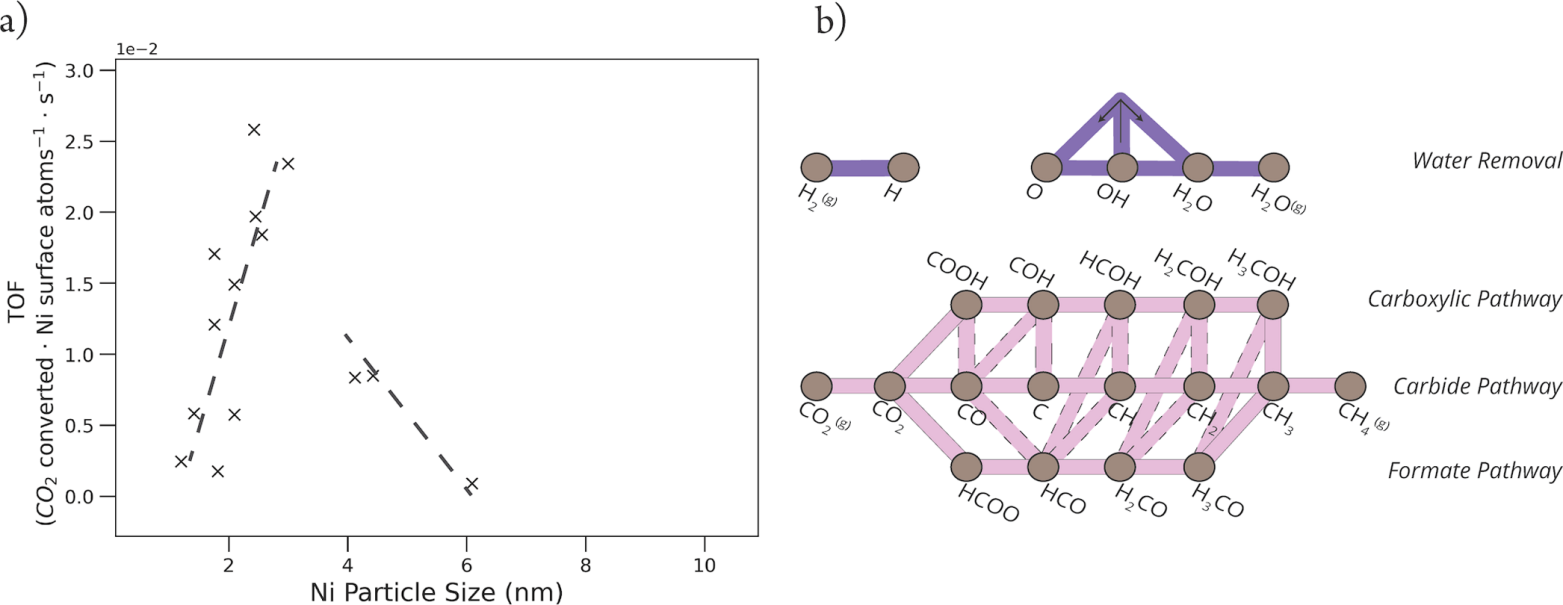
(a) Data points from published turn-over frequency (TOF) values for the catalytic CO_2_ hydrogenation over Ni/SiO_2_ catalysts.^9−11^ (b) Schematic overview of relevant possible elementary reaction steps in the catalytic CO_2_ methanation over nickel-based catalysts. The brown nodes correspond to the reactants, products, and reaction intermediates. The pink and purple edges between the nodes represent the elementary reaction steps that connect these compounds. The primary pathways are shown in pink: i.e., direct CO dissociation in the carbide pathway and hydrogen-assisted dissociation in the carboxylic and formate pathways. Interlinks are the elementary reactions that connect the primary pathways whose edges are boxed with a dashed line. Elementary reaction steps for the removal of water are shown in purple. The intermediate H_2_O* can be formed in two ways, via the protonation of OH* and via proton shuffling between two OH* intermediates.

Supported nickel metal nanoparticles are well-known to be excellent CO_2_ hydrogenation catalysts for the Sabatier reaction in which CO_2_ and H_2_ react to form CH_4_ and H_2_O at a temperature between 575 and 725 K.^7,8^ Previously published work^9−11^ of research performed in our group has shown that catalytic CO_2_ hydrogenation over well-defined supported nickel nanoparticles in the range of 1–10 nm diameter is a structure-sensitive reaction. A structure-sensitive reaction generally shows a dependency between the catalytic activity and the size of the metal nanoparticle, typically in the range of 2–20 nm.^12−15^ Catalytic testing showed a dependency of the surface-normalized activity related to particle size, with an optimum turn-over frequency (TOF) to CH_4_ for nickel metal nanoparticles of about 2.5 nm, as shown in Figure 1a.^9−11^ In addition to the changes in methane production rate, changes in the related operando infrared (IR) spectra and product distribution (i.e., CH_4_, CO, CH*_x_*O, and C_2+_) were also observed for different sizes of nickel metal nanoparticles. However, the elementary reaction steps that play a significant role in this concept of structure sensitivity have to the best of our knowledge not yet been identified. With the current stage of technological development of spectroscopy in terms of space and time resolution, it is not yet possible to experimentally follow the Sabatier reaction and the related reaction intermediates formed on isolated sites of the supported nickel nanoparticles. Therefore, during the interpretation of both the catalytic performances and operando spectroscopic data, heterogeneities at the outer surface of the nickel metal nanoparticles are averaged out.

To be able to tune the selectivity of the CO_2_ methanation reaction experimentally, we first need to understand the structure sensitivity on a level of elementary reaction steps on different nickel facets. It is, for example, important to know which elementary reaction steps are active on the catalytic surface, and which step is rate-controlling or rate-inhibiting and how this all varies with different nickel surface facets. These concepts are important for the rational design of more efficient and selective catalytic Ni nanoparticles in CO_2_ methanation.

The mechanism of CO_2_ hydrogenation over nickel catalysts has been under debate for many decades.^7,15−19^ There are studies that indicate that CO_2_ is directly hydrogenated toward HCOO* without the formation of CO*. Other studies show that first CO* is formed, after which the hydrogenation occurs either via H*_x_*CO* or via the formation of surface carbon in the direct CO* dissociation. In short, the removal of both oxygen atoms from CO_2_* could occur via direct C–O dissociation or via H-assisted C–O dissociation.

The complexity of the system is illustrated in Figure 1b in which an overview of the possible reaction pathways to convert CO_2_ into CH_4_ is presented. The three primary pathways together with their interlinks are shown in pink. These include direct CO dissociation via the carbide pathway and two hydrogen-assisted CO dissociation routes, referred to as the carboxylic and formate pathways. Furthermore, elementary reaction steps for the formation of water are depicted in purple. The formation of the H_2_O* intermediate in the Sabatier reaction can occur via two pathways; i.e., by direct hydrogenation of OH* and via proton shuffling between two OH* species to produce O* and H_2_O*.

In this work, we elucidate the mechanism of the Sabatier reaction over the four facets of a nickel metal nanoparticle with increasing size by a combination of density functional theory (DFT) and micro-kinetics modeling (MKM) simulations of all relevant elementary reaction steps. Since nickel metal nanoparticles on nonreducible oxide supports are 90–98% selective^10,20^ toward methane at 640 K, in this work, we do not consider the formation of any gaseous CO or C_2+_ products and we have chosen 640 K to be the temperature of interest in this study.

To the best of our knowledge, this is the first detailed first-principles-based micro-kinetics study of the entire reaction network of catalytic CO_2_ methanation over nickel beyond a single site approximation. Based on these results, we propose an ideal nickel metal nanoparticle in terms of exposed facets and size for catalytic CO_2_ methanation.

## 2 Methods

### 2.1 Density Functional Theory

**Figure 2:**
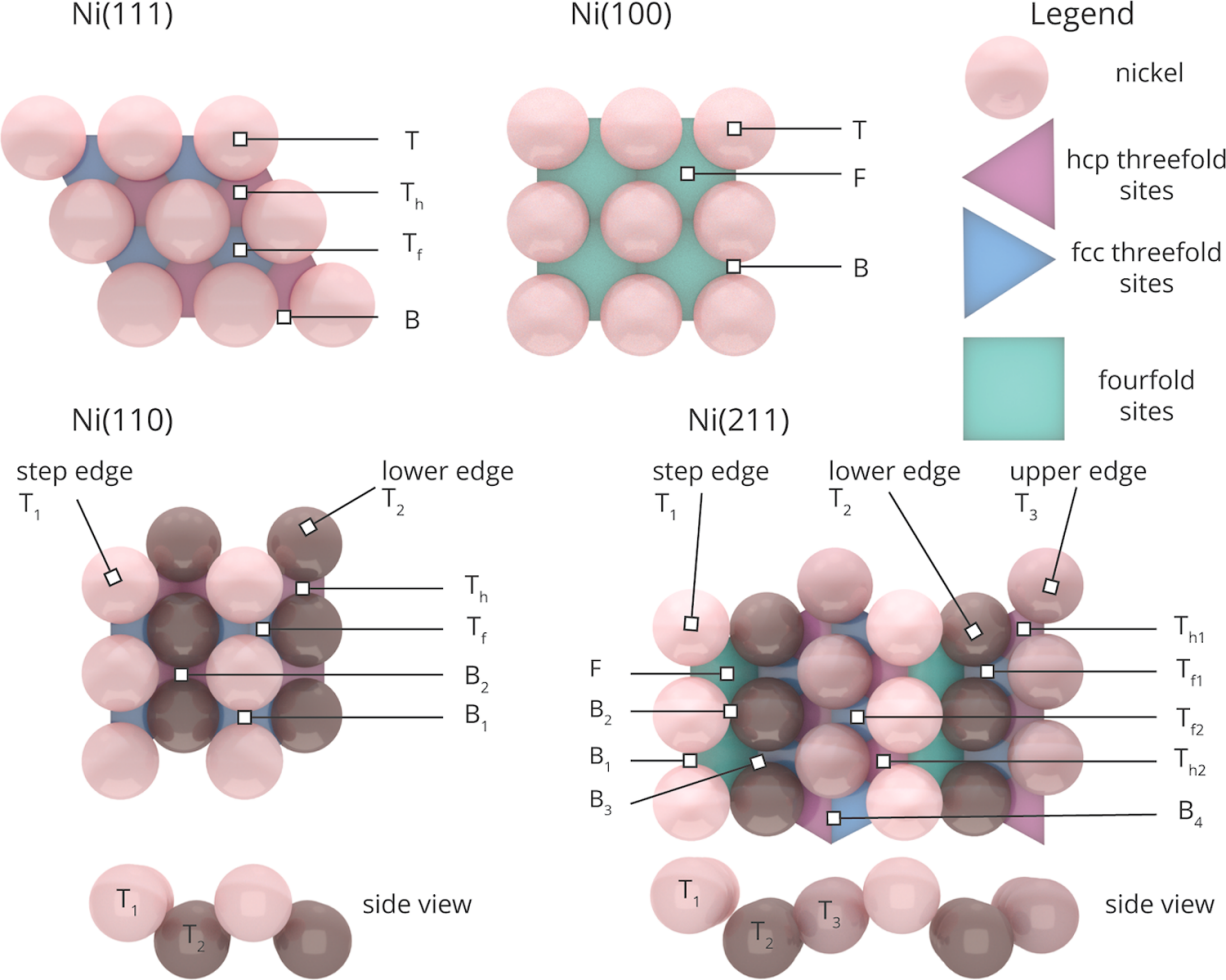
Schematic representation of the facets Ni(111), Ni(100), Ni(110), and Ni(211). Adsorption sites on Ni(111) include top (T) sites, bridge (B) sites, threefold-fcc (T_f_), and threefold hexagonal close-packed (hcp) (T_h_) sites. For the surface atoms of Ni(111), the coordination number (CN) is 9. Ni(100) gives top (T), bridge (B), and fourfold (F) adsorption sites, CN = 8. Ni(110) has two top sites (T_1_), (T_2_); two threefold sites (T_f_), (T_h_); and a bridge site, CN = 7 for the step edge and CN = 11 for the lower edge. Ni(211) has three top sites (T_1_), (T_2_), and (T_3_); two threefold-fcc sites (T_f_^1^), (T_f_^2^); two threefold-hcp sites (T_h_^1^), (T_h_^2^); fourfold sites (F); and four bridge adsorption sites (B_1_), (B_2_), (B_3_), and (B_4_) for the step edge CN = 7, lower edge CN = 10, and upper edge CN = 9.

All quantum-chemical calculations in this work were performed using a plane-wave density functional theory (DFT) approach with the projector-augmented wave (PAW) method^21,22^ in conjunction with a Perdew–Burke–Ernzerhof (PBE) exchange-correlation functional as implemented in Vienna Ab initio Simulation Package (VASP).^23,24^ A detailed rationale on the choice between a soft or hard potential and an analysis of the numerical approach can be found in Sections A and B of the Supporting Information. The kinetic energy cutoff for the plane-wave basis set was set to 400 eV. Higher cutoff energies (i.e., higher number of plane waves) did not result in a significant change in the electronic energy. A conventional face-centered cubic (fcc)-Ni unit cell was used to build the surface terminations. Herein, the bulk lattice constant of nickel in its face-centered cubic (fcc) crystal structure was optimized, yielding a theoretical optimum of 3.521 Å. This corresponds well to the experimental bulk lattice constant of 3.517 Å.^25^ Four nickel surface terminations were chosen as representative models for a nanoparticle. Closed-packed Ni(111) and open Ni(100) are used to model flat terrace sites and Ni(110) and Ni(211) are used to model step-edge sites. A schematic depiction of these facets is shown in Figure 2. Ni(111) and Ni(100) surfaces were modeled using a (3 × 3) surface with seven metal layers, constituting slab heights of 12.11 and 10.43 Å, respectively. The Ni(110) and Ni(211) surface facets were modeled using (4 × 4) and (3 × 6) surfaces, both with four metal layers giving slab heights of 7.26 and 6.92 Å, respectively. A Monkhorst–Pack mesh of *k*-points of (5 × 5 × 1) for Ni(111), Ni(100), and Ni(110) and (3 × 3 × 1) for Ni(211) were used.^26^ A vacuum layer of 15 Å perpendicular to the surface was employed to avoid the spurious interaction of neighboring supercells. To avoid the buildup of a large dipole moment between neighboring supercells, the adsorbates were placed on both sides of the surface slabs, retaining a point of inversion. Partial occupancies were determined using a first-order Methfessel–Paxton scheme with a smearing width of 0.2 eV.^27^ Electronic convergence was set to 10^–5^ eV, and geometries were converged to 10^–4^ eV (≈0.01 kJ/mol) using a conjugate-gradient algorithm that employs trial and corrector steps to converge both the energy of the structure as well as the forces on the ions. All geometries were confirmed to correspond to a local minimum on the potential energy surface by means of a frequency analysis (vide infra). For the gas-phase calculations of the reactants and products, the molecules were placed at the center of a 10 × 10 × 10 Å^3^ unit cell. Gaussian smearing with a width of 0.05 eV was used for electron smearing and only the Γ-point was used to sample the Brillouin zone. To determine transition states, we used the climbing image nudged elastic band (CI-NEB) method as implemented in VASP.^28^ A frequency analysis was performed to confirm that all transition geometries correspond to a first-order saddle point on the potential energy surface. To determine the frequencies, a Hessian matrix was constructed using a finite-difference approach with a step size of 0.02 Å for displacement of individual atoms along each Cartesian coordinate. These frequencies were also used to determine the zero-point energy (ZPE) corrections and vibrational partition functions for all adsorbed species and transition states.

### 2.2 Micro-Kinetics Modeling

All micro-kinetics modeling (MKM) simulations were carried out using the MKMCXX code, which has been used and reported extensively in previous works to investigate CO hydrogenation on Rh and Ru surfaces.^29−31^ The methods to perform an analysis of the rate of the individual elementary reaction steps and to conduct a sensitivity analysis are implemented in the MKMCXX code.^29^ A detailed overview of these methods can be found in the literature.^32,33^ For adsorption/desorption reactions, Hertz–Knudsen kinetics was used. The reaction rate constant for adsorption^34^ is
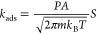
1where *P* is the partial pressure of the adsorbate in the gas phase (in Pa), *A* is the surface area of the adsorption site (in m^2^), *m* is the mass of the adsorbate (in kg), and *S* is the dimensionless sticking coefficient. The simulations were performed with an initial CO_2_/H_2_ mixture of 1:4, a total pressure of 1 bar, and temperatures between 500 and 800 K, which are typical operating conditions. The surface area was set to 2.68 × 10^–20^ and 2.19 × 10^–20^ m^2^ for Ni(111) and Ni(110), respectively. This value corresponds to the area of their threefold sites. For Ni(100) and Ni(211), the surface area of their fourfold site was used, which corresponds to 6.20 × 10^–20^ and 6.10 × 10^–20^ m^2^. For each facet, the sticking coefficients of H_2_, CO_2_, CH_4_, and H_2_O were taken from refs (35) and (36) (see Section H of the Supporting Information). The rate constant for desorption can be approximated from the enthalpy of desorption and the entropy gain of two translational degrees of freedom and all rotational degrees of freedom.^37^ For the adsorption enthalpy, we used the most stable zero-point energy-corrected adsorption heat as computed by DFT. The desorption rate constant and desorption rate are then calculated with
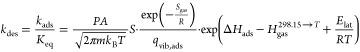
2and
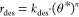
3

Herein, *n* is one for associative and two for dissociative adsorption. Both *S*_gas_ and *H*_gas_^298.15→*T*^ are calculated from thermodynamic tables^38^ using the Shomate equation.^39^ The remaining entropy of the adsorbed intermediate is described by *q*_vib,ads_, where only vibrations above 200 cm^–1^ were taken into account. *E*_lat_ was added to the rate of desorption to incorporate the qualitative effect of lateral interactions, which is considered to be surface independent. The values for *E*_lat_ were chosen the same as reported in the literature.^37^ Justification and validation of this choice was done via a sensitivity analysis (see Table 1), which is given in Section J of the Supporting Information. For a more in-depth explanation of the lateral interaction potential, the reader is referred to the literature.^37^

**Table 1:** Performed Sensitivity Analysis for the Micro-kinetic Modeling of CO_2_ Methanation over Nickel Surfaces^a^

	potential energy diagram	surface coverage	reaction rate	activation energy	reaction orders	degree of rate control	flux diagram
(1) destabilization of CO* and H*	x						
(2) correction of CO* overbinding		x	x	x	x	x	x
(3) lateral interaction potential		x		x	x		

a The results are reported in Supporting Information (1) Section G, (2) Section I, and (3) Section J.

For a hypothetical surface reaction
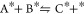
4the net reaction rate, *r_j_*, was calculated as
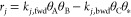
5where θ_A_, θ_B_, and θ_C_ are the fractional coverages of species A*, B*, and C*, respectively. θ_*_ corresponds to the surface fraction of empty sites. Moreover, *k*_*j*,fwd_ and *k*_*j*,bwd_ are the rate constants in the forward and backward directions, respectively. The rate constant (*k*) of an elementary reaction step can be determined using the Eyring equation, which is defined as follows
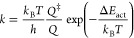
6where *Q*^‡^ and *Q* are the partition functions of the activated complex and its corresponding initial state, and Δ*E*_act_ is the ZPE-corrected activation energy. Energies and vibrational frequencies were obtained from DFT calculations. To correct for CO overbinding, we added 40 kJ/mol to the energy of the most stable CO* configuration. This is in line with the reported overbinding of 0.5 eV for CO using PBE functionals.^40^ A sensitivity analysis (see Table 1) on the correction of CO* overbinding is given in Section I of the Supporting Information. H* was destabilized with 20 kJ/mol to account for the assumption that catalysis for H* does not happen from the most stable adsorption site since these will be mainly occupied by other reaction intermediates. This is in close agreement to the best destabilization of 0.2 eV in the literature.^41^

Entropic contributions to the rate constants are included in the *Q*^‡^/*Q* term. These ratios are calculated using the vibrational partition function
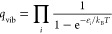
7where ε*_i_* is the *i*th eigenvalue of the mass-weighted Hessian. The reaction rates of the surface reactions are calculated with
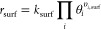
8where θ*_i_* and υ*_i_* represent the surface coverage and stoichiometric coefficient of species *i*, respectively.

The ordinary differential equations for each surface component are defined by
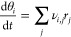
9

These ordinary differential equations are time-integrated until a steady state is found for all surface intermediates. To describe the dependency of the overall reaction rate on the height of the transition state of the individual elementary reaction steps, the degree of rate control (DRC) concept, as proposed by Campbell, was used.^42^ Herein, the degree of rate control coefficient is defined as
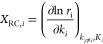
10

A positive DRC coefficient indicates that the elementary reaction step is rate-controlling, whereas a negative coefficient suggests that the step is rate-inhibiting. In the case that only one single elementary reaction step has a DRC coefficient of 1, that step is termed the rate-determining step from the perspective of Langmuir–Hinshelwood–Hougen–Watson kinetics.

An overview of necessary input values to create a micro-kinetics model for each nickel facet is given in Section H of the Supporting Information. Kinetic coupling between nickel facets is not included in this study.

### 2.3 Wulff-Constructed Metal Nanoparticles

For the design of Wulff-constructed nickel metal nanoparticles, we have used the WulffPack software.^43^ An fcc crystal structure with a lattice parameter of 3.521 Å was used, equal to the theoretical optimum lattice parameter used for the DFT calculations. The surface energies for Ni(111), Ni(100), Ni(110), and Ni(211) were determined with DFT-based calculations and set to 2.1930, 2.4596, 2.4571, and 2.3843 J/m^2^, respectively. These values are fully in line with previous reports.^44^ Edge and vertex energies were not taken into account. The local structures of the surface atoms of each slab model and each constructed nanoparticle were determined by pattern recognition algorithm based on the common neighbor analysis (CNA) method.^45,46^ The pattern recognition is based on a preestablished library of common crystal terminations of fcc, hcp, and single crystal (SC) bulk crystals.^46^ The activity of each Wulff-constructed metal nanoparticle was calculated as a linear combination of the activity of the exposed facets, with the following formula
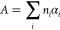
11where *A* is the total activity of a nanoparticle, *n_i_* is the number of surface atoms of facet *i*, and α*_i_* is the theoretical activity per atom of facet *i* at 640 K.

## 3 Results

### 3.1 Density Functional Theory

To study the structure sensitivity of CO_2_ methanation over nickel metal nanoparticles, four periodic slab models were chosen to be representative for the catalytic surface of nanoparticles in the size range of 1–10 nm. These model facets are two terraces, i.e., Ni(111) and Ni(100), and two stepped surfaces, i.e., Ni(110) and Ni(211). A schematic representation of the four facets is given in Figure 2. For each of these nickel facets, we have performed DFT calculations for all relevant elementary reaction steps in the catalytic CO_2_ methanation, as depicted in Figure 1b.

To explore the overall thermodynamics of the CO_2_ methanation pathways over the four model systems, we first study the stability of all relevant reaction intermediates. The adsorption energy, *E*_ads_, is a measure of the strength of the adsorbate–substrate interaction. *E*_ads_ is defined as follows
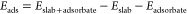
12where *E*_slab+adsorbate_ represents the total energy of the optimized adsorbate on the surface, *E*_slab_ is the energy of the nickel slab, and *E*_adsorbate_ is the energy of the adsorbate in the gas phase. For all energy terms, the zero-point energy (ZPE) has been added. In Sections D and E of the Supporting Information, the calculated adsorption energies are listed and visualized. When possible, we compared our results to previous results reported in the open literature, as shown in Section C of the Supporting Information. The majority of the calculated energies are in line with other studies. Notable differences between ours and results reported in the literature are attributed to differences in computational codes, settings (e.g., a different exchange-correlation functional), or geometries.

Based on the adsorption energies, we can establish the following general trends. Highly coordinatively unsaturated adsorbates, such as C* and CH*, favor three- and fourfold coordination sites. More saturated adsorbates, such as CH_3_*, are preferentially located in bridge and top positions of the surface. These trends are also observed in other studies.^30,47,48^ The preference for these intermediates is attributed to the bond-order conservation principle^49^ and the hybridization of the atomic orbitals in these configurations.^50^ These principles can also be readily utilized to rationalize the observed adsorption strength for the same species between different Ni facets. For example, ordering the adsorption energy of H_2_O* on a top site from least to most exothermic shows the following trend: Ni(111) > Ni(100) > Ni(110)/Ni(211). This trend of more exothermic adsorption energy can be directly linked to the coordination number of the metal atoms involved (CN = 9, 8, and 7 for Ni(111), Ni(100), and Ni(110) ≈ Ni(211), respectively).

Conventionally, the adsorption energies of the reaction intermediates are reported with respect to their gas-phase configuration. In principle, the reference state can be arbitrarily chosen; however, not all intermediates have stable gas-phase configurations and such intermediates consequently show very high chemisorption energies. This makes comparison of the surface stability between different adsorbates quite difficult. From a catalytic point of view, it thus makes more sense to use the most stable adsorption configuration of the individual atoms that constitute the molecules as the reference state.^30^ In line with this reasoning, we adopt the following definition for the intermediate stability as given by
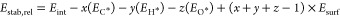
13where *E*_stab,rel_ is the relative stability, *E*_int_ is the electronic energy of the intermediate on the surface, *E_j_* is the electronic energy of C*, H*, or O* in their most stable configurations on the surface, *E*_surf_ is the electronic energy of an empty surface, and *x*, *y*, and *z* are the number of atoms of C*, H*, and O* constituting the intermediate, respectively. All energies are in kJ/mol. In this part of the work, explicit lateral interactions between intermediates were not yet included.

**Figure 3:**
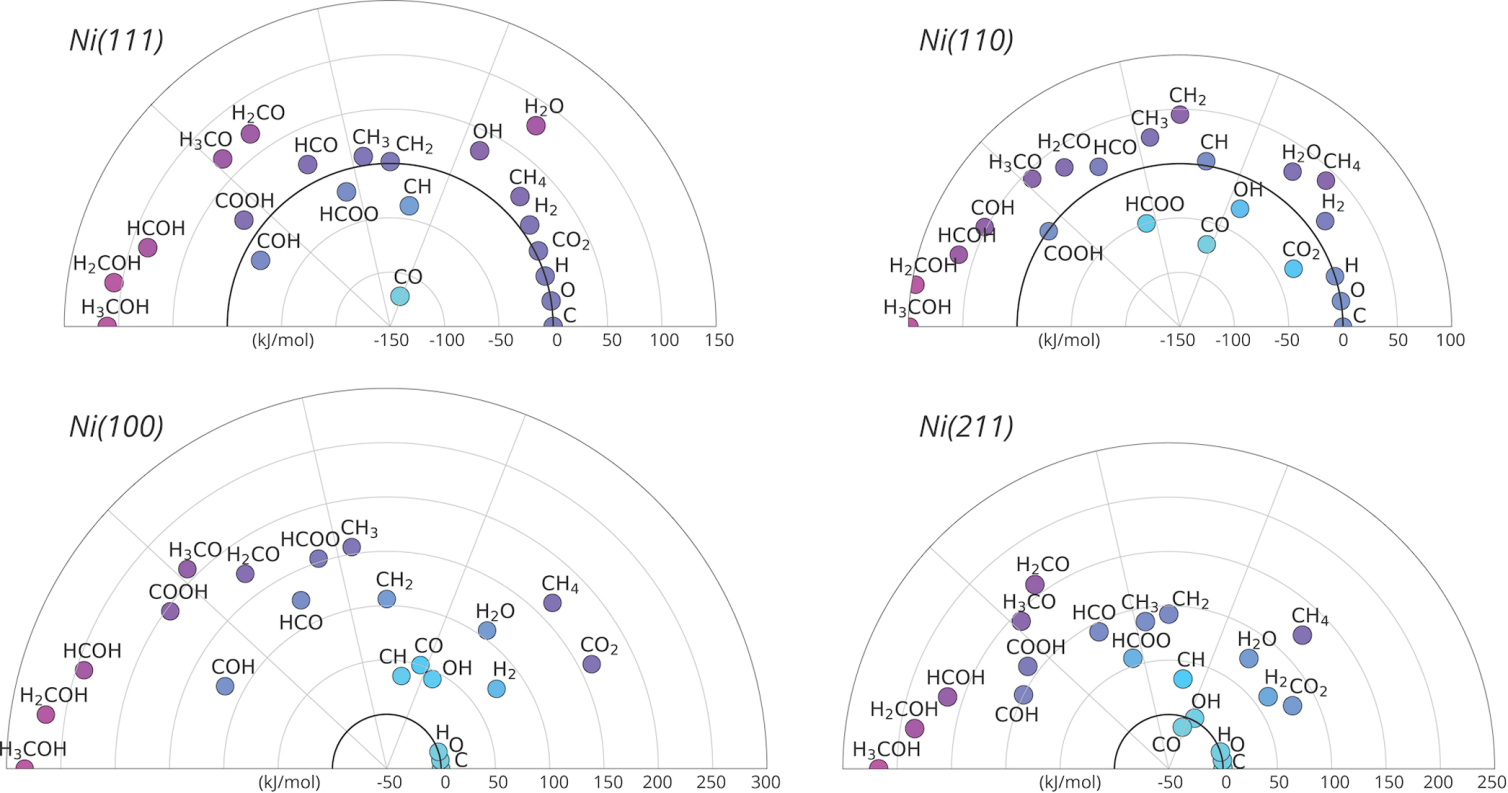
Stability plot per nickel metal facet with surface reaction intermediates in their most stable geometry. The energy (kJ/mol) is calculated using the reference energy of atomic carbon, hydrogen, and oxygen adsorbed on the surface in their most stable configuration. Intermediates located closer to the center have a lower *E*_stab,rel_ and are therefore more stable. For clarity, the reaction intermediates were grouped into four sections. The first three sections, from left to right, are based upon the carboxylic-, formate-, and carbide pathways for catalytic CO_2_ hydrogenation over nickel metal nanoparticles. The fourth section on the right contains products, reactants, and intermediates for water formation. Also note, the plots for Ni(100) and Ni(211) have a different energy axis as compared to Ni(111) and Ni(110).

In Figure 3, the surface stability of all intermediates for each of the four nickel facets is shown. Intermediates located closer to the center of the plot, thus with a lower *E*_stab,rel_, are more stable on the surface than those located more to the outside. This analysis already gives a qualitative impression on the catalytic performance and aids in understanding the efficiency of the kinetic pathways. Ideal catalysts showing high activities have many intermediates at roughly the same relative stability. Conversely, catalytic pathways wherein the product states of the elementary reaction steps are significantly more endothermic as compared to their reactant states will consequently show large energy barriers.

From Figure 3, it can be observed that for intermediates adsorbed on the Ni(100) surface, the relative stabilization energy is positive, thus formation of these complexes from their constituting atoms is endothermic. This is the direct result of the significant bond strength between Ni and C* or O* in a fourfold site. Methane formation from adsorbed CH*_x_*O_y_* is therefore expected to be endothermic and consequently associated with relatively high energetic barriers. This phenomenon is to a lesser extent also observed for the Ni(111) and Ni(211) surfaces. In contrast, for the Ni(110) surface, it is found that some of the intermediates, especially those resulting from CO* and CO_2_* hydrogenation are more stable than C*, O*, and H*.

In general terms, it is found that C_1_ oxygenates corresponding to the carboxylic pathway (located on the leftmost parts in the stability plots) are the least stable species on each nickel facet. While the formation energies of both COOH* and COH* are comparable to those found in the carbide and formate pathways, progression via this mechanism toward more hydrogenated carboxylic species is increasingly endothermic and therefore less favorable. This suggests that the carboxylic pathway is unlikely to be involved in methane formation from CO_2_.

For the formate and carbide mechanisms, it is found that the stabilities of the intermediates are quite similar on the Ni(111), Ni(100), and Ni(110) facets. For Ni(211), however, it can be seen that the intermediates of the carbide pathway are significantly more stable than the intermediates of the more hydrogenated species of the formate pathway. Therefore, it is expected that the rate of methane formation via the formate and carbide pathways is fairly similar for Ni(111), Ni(100), and Ni(110), whereas for Ni(211), it is expected that the carbide pathway is preferred.

**Figure 4:**
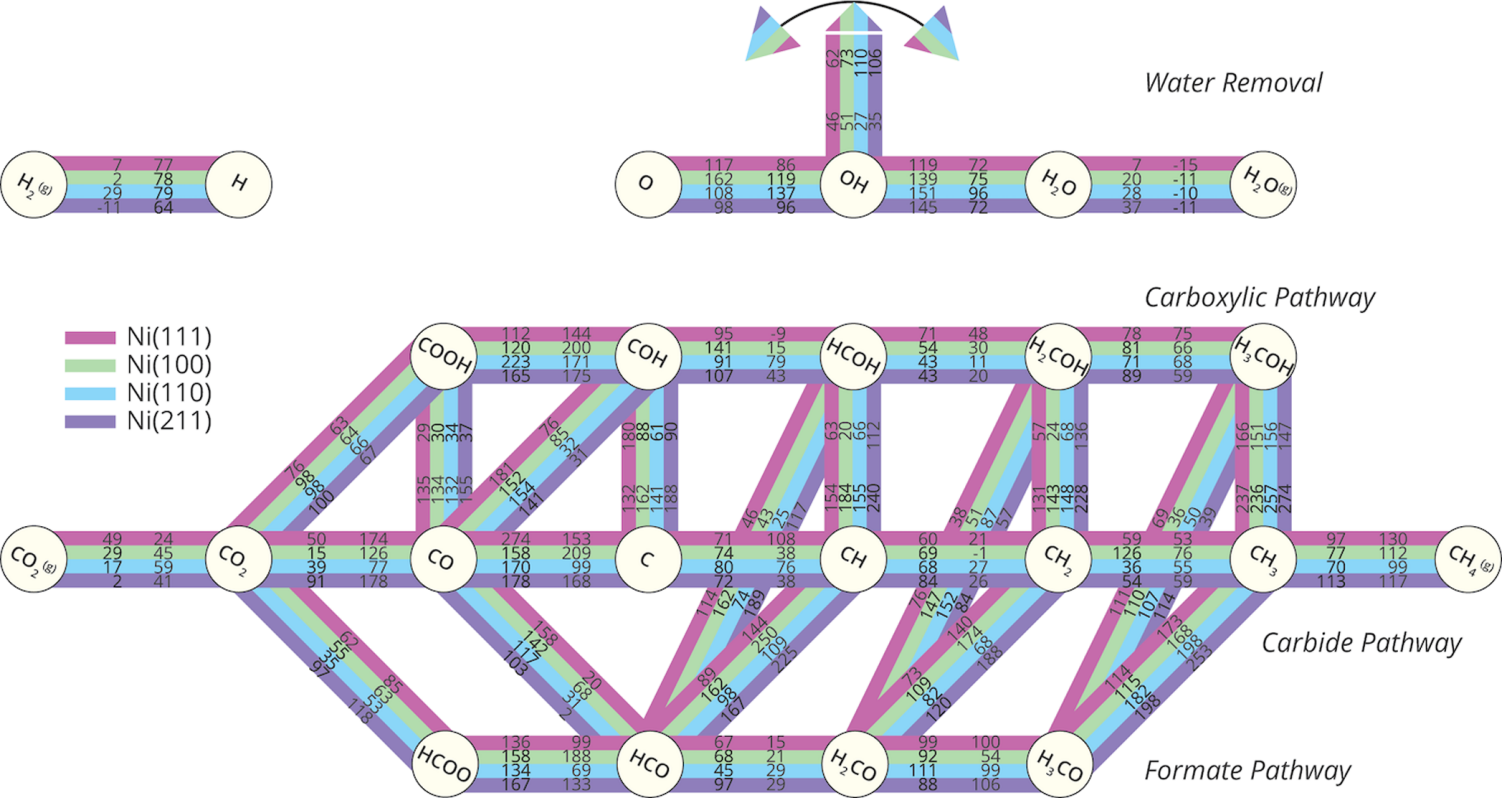
Forward and backward activation energies (kJ/mol) for the catalytic conversion of CO_2_ to CH_4_ on Ni(111), Ni(100), Ni(110), and Ni(211) metal surfaces. Each of the four colors represent a certain nickel metal facet. The first number, going from one intermediate to the next, is the forward activation energy (*E*_f_), and the backward activation energy (*E*_b_) is listed as the second number. For example, the activation energy of hydrogenation of C* on Ni(111) is 71 kJ/mol, and the backward activation energy is 108 kJ/mol.

Next, we present the activation energies and transition-state structures. The forward and backward energy barriers for each elementary reaction step are presented in the form of a reaction network depicted in Figure 4. The geometries of the initial, transition, and final states of each elementary reaction step over the four different metal nickel facets are presented in Section F of the Supporting Information. All energy barriers are given with respect to the most stable configuration of the corresponding adsorbed states. In other words, migration barriers with respect to these stable states are accounted for in the activation energies. The corresponding potential energy diagrams (PEDs) for the carbide, carboxyl, and formate pathways for each of the four facets can be found in Section G of the Supporting Information.

The potential energy surfaces give us insight as to which elementary reaction steps are expected to be critical in the formation of methane from CO_2_ for each of the different pathways and each of the different surface facets. A detailed analysis of the complete chemokinetic network is complex as methane formation can not only proceed according to a trajectory associated with a single one of the three pathways but also by a multitude of combinations between these three pathways. This can be readily seen by the broad set of elementary reaction steps that connect the three different pathways. As such, to analyze the relatively complex chemokinetic network, we look upon three critical steps in the formation process of methane revolving around the modes of oxygen removal. These are

- initial C–O bond scission to form a single oxygenated species,
- secondary C–O bond scission to form a nonoxygenated C_1_ hydrocarbon, and
- removal of oxygen as water.

Initial activation of CO_2_* can proceed either in a direct fashion according to the carbide pathway or in a hydrogen-assisted manner according to the carboxylic or formate pathway. For the Ni(111) and Ni(100) terraces, it can be seen from Figure 4 that the lowest activation energies are found for direct CO_2_* dissociation. This step has activation energies of 50 and 15 kJ/mol for Ni(111) and Ni(100), respectively. The alternative pathway that involves the hydrogenation of C or O atom in CO_2_* is found to exhibit higher barriers. For Ni(110), the formation of CO* and HCOO* has a comparable activation barrier. However, the removal of the first oxygen from HCOO* has an activation barrier of 134 kJ/mol, which is ∼3 times higher as compared to the direct dissociation of CO_2_* to CO*. This also suggests that for Ni(110), the first oxygen is removed via the carbide pathway. For Ni(211), direct CO_2_* dissociation and CO_2_* hydrogenation toward HCOO* and COOH* all have similar reaction barriers (around 90–100 kJ/mol). The formate pathway via HCOO* is however kinetically hindered as the subsequent C–O bond scission in HCOO* has a very high barrier of 167 kJ/mol. In contrast, C–O bond scission in COOH* to form CO* and OH* in the carboxylic pathway is activated by only 37 kJ/mol. Thus, these results suggest that for Ni(211), both the carbide and carboxylic pathways can be utilized for the first C–O bond breaking event. Conclusively, for Ni(111), Ni(100), and Ni(110) surfaces, it is found that the carbide pathway is the most feasible pathway. For the Ni(211) facet, it is also found that the carboxylic pathway might be involved. Irrespective of the latter, these results show that CO* is a critical node in the network and that all kinetic routes are expected to proceed via this intermediate.

After initial C–O bond scission, the secondary oxygen atom needs to be removed. From the discussion for the removal of the first oxygen, it is clear that the removal of the second oxygen is expected to proceed from the CO* intermediate. Similar to CO_2_* activation, this can also proceed either in a direct or in a hydrogen-assisted manner. Here, it is found that when direct CO dissociation has a high barrier (i.e., >150 kJ/mol), which is the case for all of the facets, the hydrogen-assisted route is preferred. This is a well-known trend also observed for other transition metals such as Co, Ru, and Rh.^51,52^ For Ni(111), the pathway via HCO* (*E*_act_ = 158 kJ/mol) is preferred over the pathway via COH* (*E*_act_ = 181 kJ/mol). For Ni(100), both COH* and HCO* formation have similar activation energies (152 and 142 kJ/mol, respectively); however, the subsequent C–O bond scission in COH* has much lower activation energy (88 kJ/mol) as compared to C–O bond scission in HCO* (162 kJ/mol). For Ni(110), the pathway with the lowest barriers proceeds via HCO* (117 kJ/mol). Finally, for Ni(211), the lowest barrier is found for HCO* formation (103 kJ/mol) followed by COH* formation (141 kJ/mol); however, the reverse barriers for these hydrogenation reactions are very low (2 and 31 kJ/mol, respectively), which is lower than any step further toward the formation of CH_4_. As such, it is expected that the system goes backward to CO*, and direct CO dissociation, despite having the highest forward barrier, is the preferred route for secondary C–O bond scission.

The final critical aspect to consider is the removal of water. The presence of O* and OH* groups on the surface and the efficiency at which these can be hydrogenated and removed from the catalytic surface as water have a critical impact on the preferred mode of C–O bond scission. There are two routes toward water formation starting from oxygen. Initially, O* can be hydrogenated to form OH*. Next, either two OH* species undergo a proton transfer to form H_2_O* and O* or alternatively, the OH* moiety is directly hydrogenated to form H_2_O. From Figure 4, it can be seen that irrespective of the catalytic surface, the proton shuffling has a much lower activation energy as compared to direct OH* hydrogenation. As such, it is expected that the main pathway for oxygen removal is via proton shuffling. Nevertheless, micro-kinetic simulations remain necessary to confirm such hypotheses as reaction rates are not only solely dependent on activation energies but also on the surface concentrations of the intermediates and lateral interactions. Critically, the rate of proton shuffling scales quadratically in the surface concentration of OH*, whereas the direct pathway scales linearly in OH*.

### 3.2 Micro-Kinetics Modeling Simulations

Energies of stable geometries and activation barriers alone will not explain as to which reaction pathway is predominant and which elementary steps are rate-controlling. As alluded to the previous section, the availability of reaction intermediates and vacant sites is essential to determine which reaction pathway will be more favorable over others. For this purpose, we performed micro-kinetics modeling and studied the complete CO_2_ methanation network under steady-state conditions at the zero-conversion limit.

**Figure 5:**
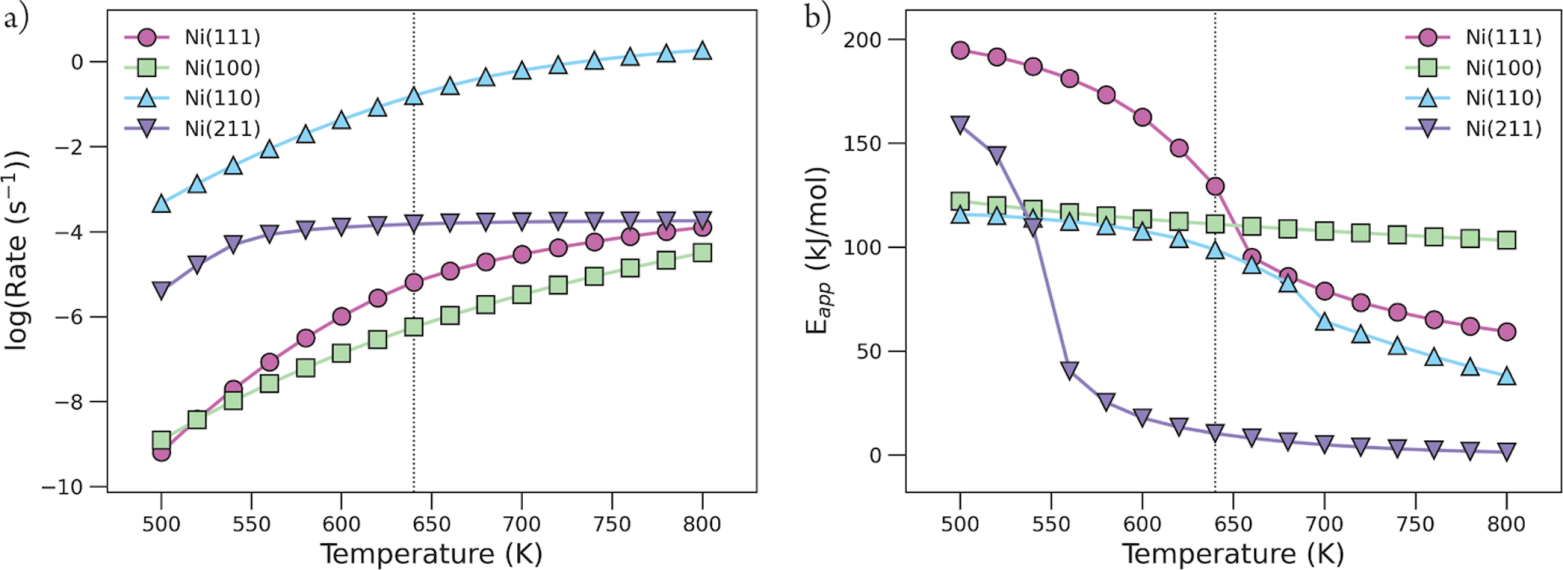
(a) CO_2_ methanation rate for each nickel metal facet in a logarithmic scale as a function of temperature (K). (b) Apparent activation energy (kJ/mol) as a function of reaction temperature (K). The vertical dotted line indicates the temperature of interest (640 K).

In Figure 5, the reaction rate and apparent activation energy as a function of the temperature are given. From this figure, it is clear that the highest rates are observed for the stepped Ni(110) surface. Clearly, the Ni(211) stepped surface and Ni(111) and Ni(100) terraces are several orders of magnitude less active. This result already highlights the need for a specific type of highly active sites to obtain an appreciable rate of methane formation. Reported apparent activation energies for CO_2_ methanation over nickel catalysts supported on various metal oxides range from 77 to 92 kJ/mol.^7,9,53−55^ The calculated apparent activation energy of 98.8 kJ/mol for Ni(110) at 640 K gives a satisfactory agreement with the literature. At this temperature, the activation energy of Ni(211) is much too low (10.3 kJ/mol), while the activation energies for Ni(111) and Ni(100) are too high with 129.3 and 111.1 kJ/mol, respectively. At lower temperatures, the apparent activation energies of Ni(110) and Ni(100) are very much alike. However, the associated reaction rates differ remarkably, which relate to a significant difference in the composition of available surface species necessary for the formation of methane. We will discuss surface coverages per nickel facet in more detail further in this section.

The apparent activation energies as a function of temperature reveal a typical trend commonly observed in heterogeneous catalysis. At too low temperature, the surface is mainly poisoned by one of the intermediates (here CO* or C*) and a sufficient amount of heat needs to be invested to activate and remove these intermediates from the surface. With increasing temperature, the number of active sites increases, resulting in a decrease of the apparent activation energy. Typically, a strong inflection in the curve is observed at the transition from an inactive to an active catalyst. For the stepped Ni(211) surface, this transition occurs around 500 K where a rapid decrease in the apparent activation energy is observed. For the Ni(110) and Ni(111) surfaces, the inflection is less pronounced and happens at somewhat higher temperatures of ∼600 K. For the Ni(100) facet, there is no inflection point observed in the apparent activation energy as a function of temperature in the plotted temperature regime. This is indicative of a surface process that increases with temperature but inhibits the overall rate toward methane formation. This is typically the result of an elementary reaction step wherein the product state is thermodynamically very stable, leading to poisoning of the catalyst surface.

To better understand the fundamental factors underlying the catalytic activity for each of the facets, we consider the surface coverage as a function of temperature, perform an analysis of the rates of the individual elementary reaction steps, and conduct a sensitivity analysis utilizing Campbell’s degree of rate control.^42^ A complete overview of the results of the DRC analysis is given in Section H of the Supporting Information. We will first discuss the results for each of the facets and then make a comparison of the most salient details.

#### 3.2.1 Ni(111)

**Figure 6:**
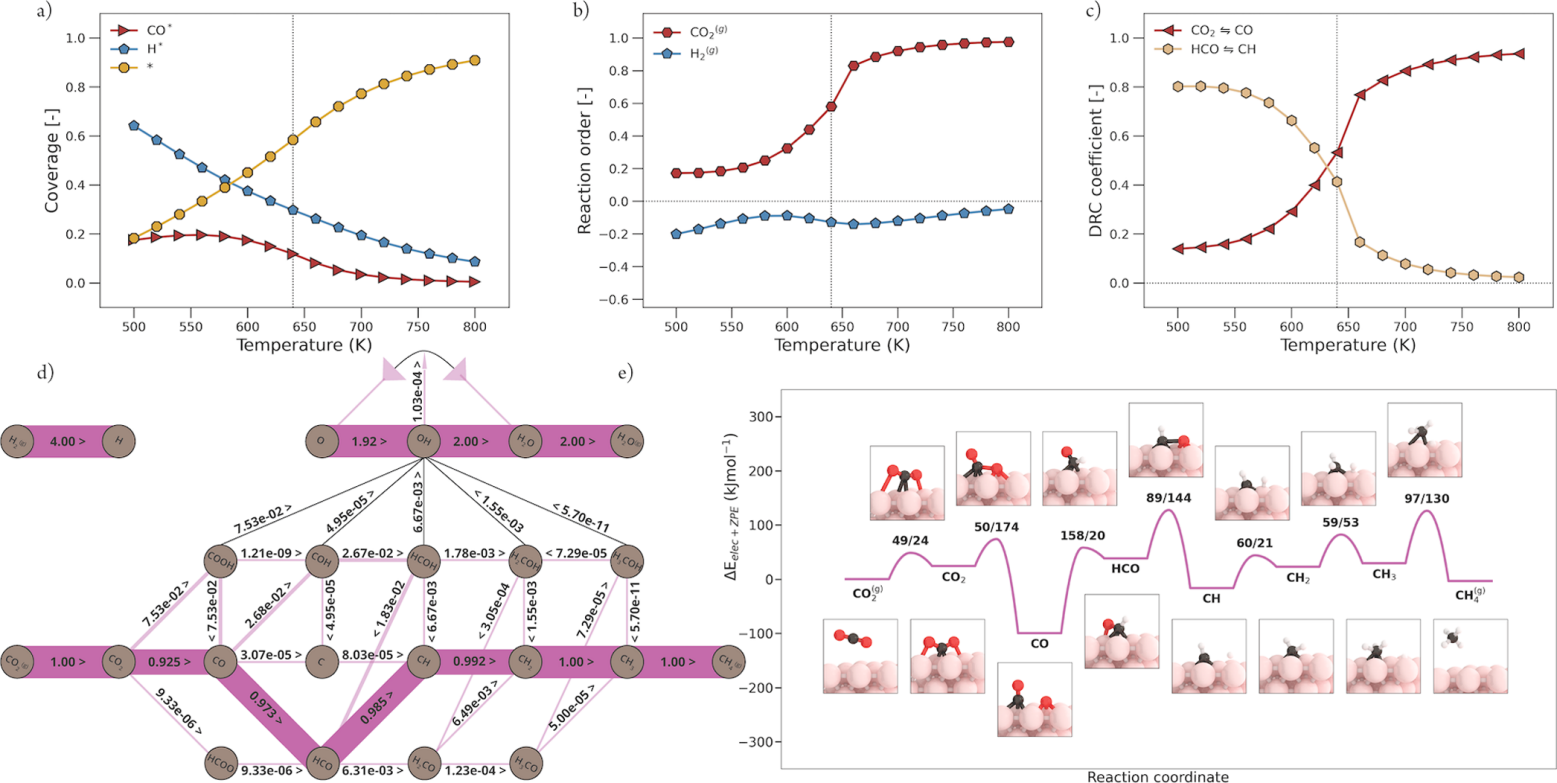
Micro-kinetics modeling (MKM) simulations of CO_2_ methanation over Ni(111). (a) Surface coverages as a function of temperature (K). Vertical line indicates the temperature of interest (640 K). (b) Reaction order. (c) Elementary reaction steps with their significant degree of rate control (DRC) coefficients plotted as a function of temperature. (d) Flux diagram at 640 K and 1 bar. The thickness of the bar that connects the nodes of the reaction network scales with the size of the flux between these nodes. The direction of the flux is indicated with “<” or “>”. (e) Potential energy diagram with geometry visualizations of the reaction pathway corresponding to the largest flux, as shown in panel (d).

In Figure 6a, the surface coverage for Ni(111) is shown as a function of temperature. Here, it can be seen that at a low temperature, Ni is mainly covered by H* and CO*, indicative that initial C–O bond scission of CO_2_* is facile but that subsequent CO activation is associated with a high reaction barrier. An increased temperature results in an increased rate of CO dissociation, and as a consequence, CO* and H* coverage decrease, while vacant sites increase. The reaction orders of CO_2_ and H_2_ as a function of temperature are depicted in Figure 6b. At low temperatures, the reaction order in CO_2_ is slightly positive. With increasing temperature, this reaction order increases with a strong inflection around 600 K, thereby showing an inverse correlation to the apparent activation energy *E*_app_ shown in Figure 5b. The increase in CO_2_ order can be related to a decrease in CO* coverage. At higher temperatures, a very limited amount of CO* covers the surface and a sufficient number of vacant sites appear. At 800 K, the surface is nearly free of adsorbates. Here, the surface is lacking in C-containing intermediates, and a more positive reaction order in CO_2_ is seen. For H_2_, a slightly negative reaction order is observed over the complete temperature range. The results of the degree of rate control (DRC) analysis are presented in Figure 6c. Herein, we observe that at a low temperature, the DRC coefficient of HCO* dissociation to CH* is strongly positive (rate-controlling). With increasing temperature, this DRC coefficient decreases to 0. An inverse trend is observed for the DRC coefficient of CO_2_* dissociation to CO*, with a slightly positive value at lower temperatures and an increase toward unity with an increasing temperature. This indicates that CO_2_* dissociation to CO* is rate-determining at a high temperature, which means that the overall reaction rate depends only on the rate of this elementary reaction step. The rate of elementary reaction steps prior to the rate-determining reaction step, in this case CO_2_^(g)^ adsorption, is at pseudo-equilibrium.^32^ At the temperature of interest, both HCO* and CO_2_* dissociation to CH* and CO*, respectively, are equally rate-controlling. These results can be readily explained using the flux analysis at 640 K as shown in Figure 6d and the reaction barriers as shown in Figure 4. CO* activation on Ni(111) is associated with relatively high barriers. At a low temperature, CO_2_* dissociation is faster than the subsequent CO* dissociation and the former elementary reaction step thus results in a small buildup of CO* intermediates. From the flux diagram, it is evident that the dominant reaction pathway goes mainly via the carbide pathway with a H-assisted CO* dissociation via the HCO* intermediate. This is in close agreement with the literature,^41^ where it was claimed that the only significant source of CH* originates from the dissociation of HCO*. For a more detailed comparison, the reader is referred to Section H of the Supporting Information. The potential energy diagram corresponding to the flux at 640 K is depicted in Figure 6e. Herein, we observe that HCO* dissociation toward CH* and O* has the highest forward activation barrier and is thus a rate-controlling step. Conclusively, at 640 K, the rate of CO_2_* dissociation becomes comparable to the rate of HCO* dissociation. As a consequence, both reactions become equally important steps in the methanation reaction and thus share roughly the same DRC coefficient of 0.5.

#### 3.2.2 Ni(100)

**Figure 7:**
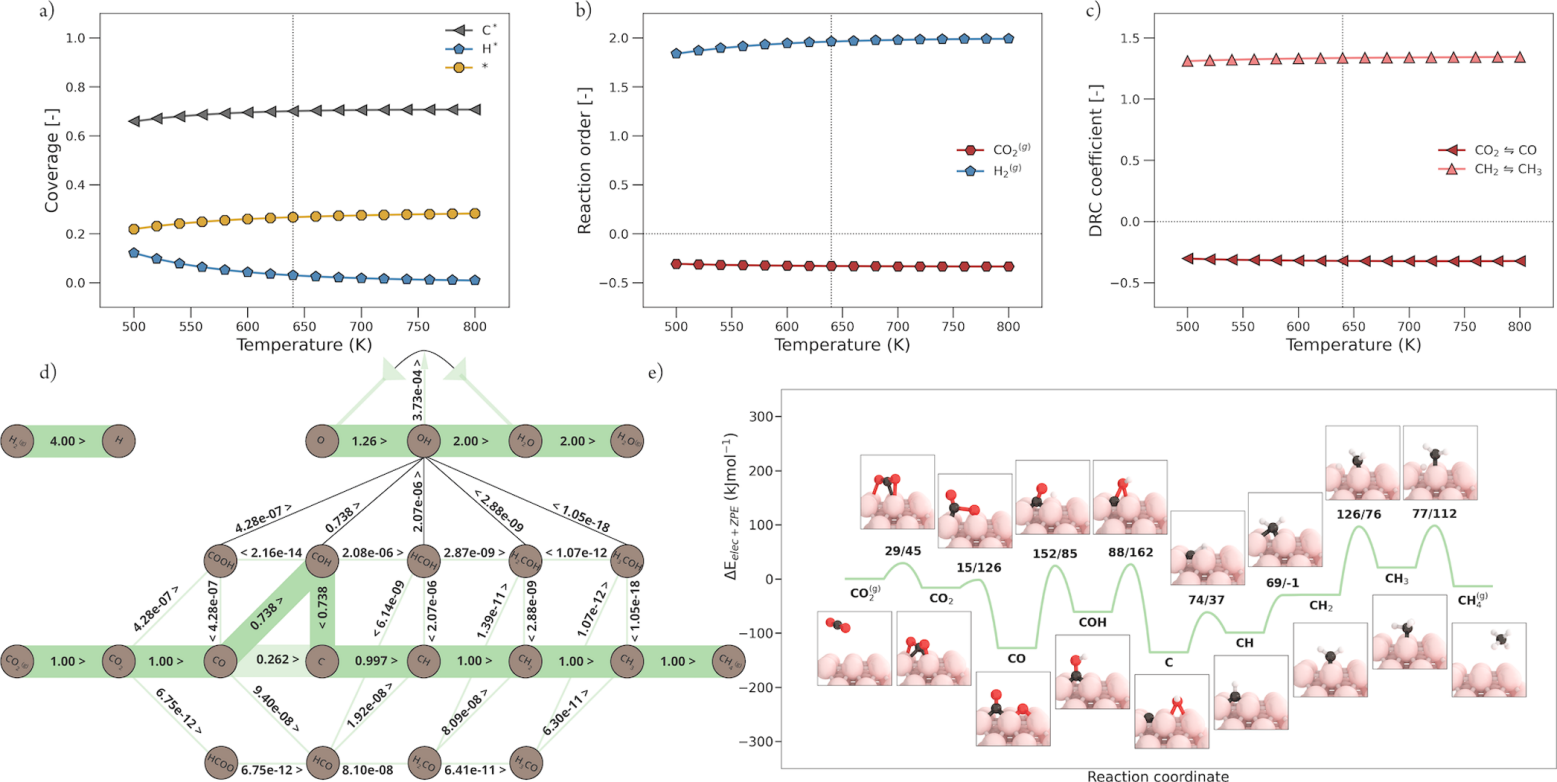
Micro-kinetics modeling (MKM) simulations of CO_2_ methanation over Ni(100). Explanation of visual style is further given in Figure 6. (a) Surface coverages as a function of temperature. (b) Reaction order. (c) Elementary reaction steps with their significant degree of rate control (DRC) coefficients. (d) Flux diagram at 640 K and 1 bar. The bars that connect the nodes CO* and C* represent a significant—but not the largest—flux and is therefore transparent. (e) Potential energy diagram with geometry visualizations of the reaction pathway corresponding to the largest flux, as shown in panel (d). See Section G of the Supporting Information for the PED of the other significant pathway.

From the *E*_app_ plot (Figure 5b), it is clear that only in the case of Ni(100), the activation energy remains roughly constant with an increase in temperature. The surface coverage plot in Figure 7a shows that this facet is highly covered with C* over the whole temperature range. Only at a lower temperature, a small fraction of the surface is covered with H*. The high coverage of C* on Ni(100) poisons the catalytic surface and hinders the production of methane. This originates from the fact that Ni(100) has a fourfold site where C* is tightly bound. From the relative stability plots (Figure 3), it is seen that the atomic intermediates indeed are most stable compared to any other intermediate. This very well could explain carbon whisker formation observed for steam and dry methane reforming.^56^

The negative and positive reaction orders for CO_2_ and H_2_, respectively (Figure 7b), indicate that either a decrease in the partial pressure of CO_2_ or an increase in the partial pressure of H_2_ could facilitate the reaction rate. Ultimately, this would reduce the amount of C* present on the surface as such that vacant sites can appear, which is a prerequisite for any surface reaction to take place.

The results of the DRC analysis depicted in Figure 7c reveal one rate-limiting and one rate-controlling elementary reaction step. Both can be explained in view of the necessity to reduce the amount of poisonous C* to increase the rate of methane production. The hydrogenation of CH_2_* toward CH_3_* is a rate-limiting step with the highest *E*_forward_ from C* toward CH_4_ (Figure 4). To pull the equilibrium state of C* toward product formation, one needs to diminish specifically this barrier. The dissociation of CO_2_* to CO* is rate-inhibiting, which originates from the fact that if the rate of CO* formation is restrained, the subsequent formation of poisonous C* will be diminished as well. The reaction flux on Ni(100) goes mainly via the direct carbide pathway (Figure 7d). For CO* dissociation, there are two reaction routes with a significant flux. The potential energy diagram of the most probable reaction flux is depicted in Figure 7e, which is the H-assisted CO* dissociation via COH*. However, direct CO* dissociation is also likely to take place. Due to the poisoning of C* on Ni(100), CO_2_ methanation cannot occur at a characteristic temperature of 640 K.

#### 3.2.3 Ni(211)

**Figure 8:**
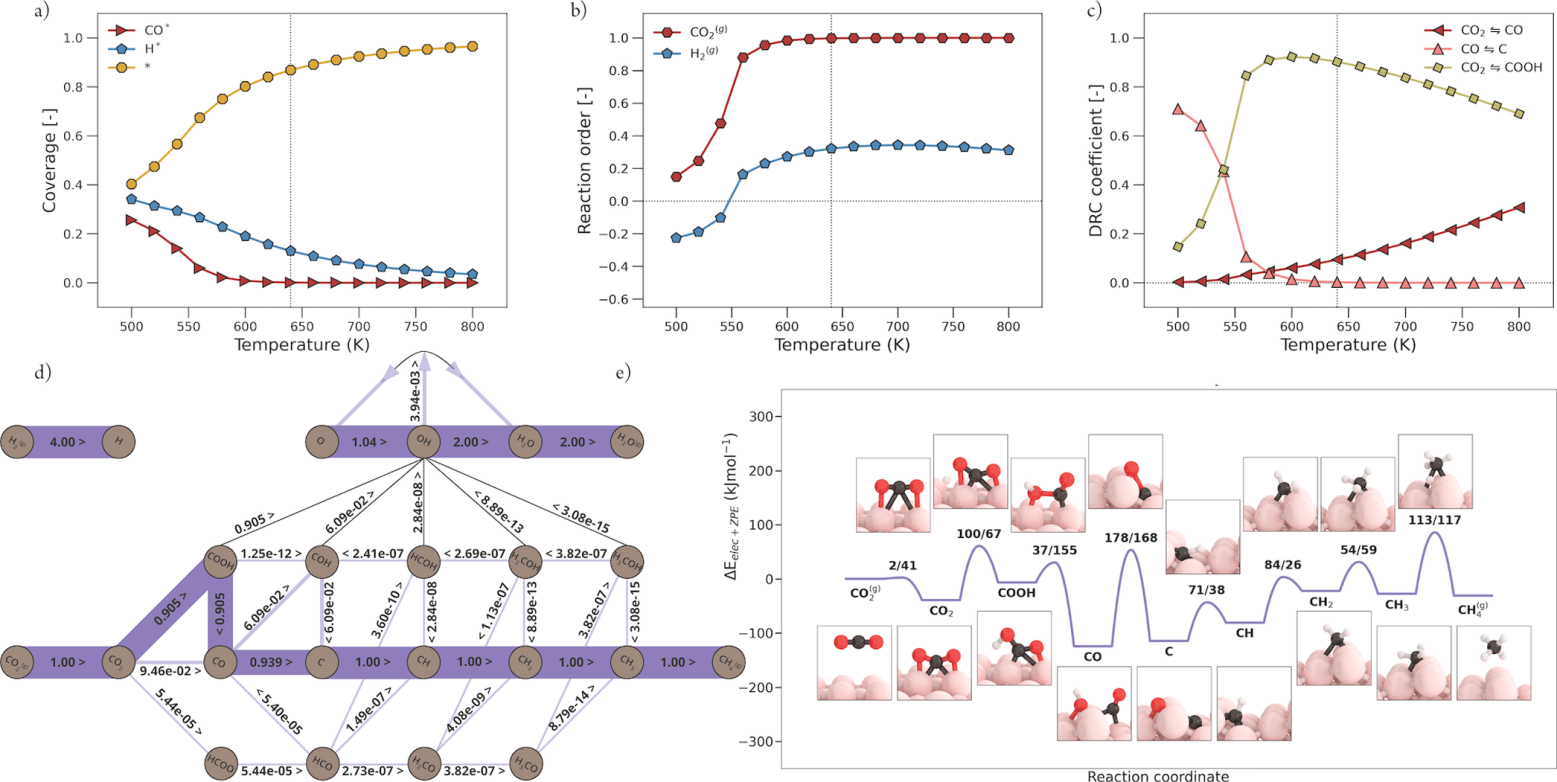
Micro-kinetics modeling (MKM) simulations of CO_2_ methanation over Ni(211). Explanation of visual style is further given in Figure 6. (a) Surface coverages as a function of temperature. (b) Reaction order. (c) Elementary reaction steps with their significant degree of rate control (DRC) coefficients. (d) Flux diagram at 640 K and 1 bar. (e) Potential energy diagram with geometry visualizations of the reaction pathway corresponding to the largest flux, as shown in panel (d).

According to the rate plot in Figure 5a, Ni(211) is the second best performing facet in CO_2_ methanation. However, Ni(211) is 3 orders of magnitude less active compared to the best performing facet Ni(110). From Figure 8a, the surface coverage as a function of temperature is shown. Herein, it can be seen that at a low temperature, both CO* and H* cover the Ni(211) surface mildly. With a slight increase in temperature, CO* coverage drops to zero, which indicates a sufficient increase in CO* dissociation rate. The surface fraction of H* also drops with increasing temperature, but less steeply. At the temperature of interest, the surface consists completely out of vacant sites, apart from 0.1 coverage of H*. The lack of any carbonaceous reaction intermediate explains why Ni(211) is significantly less active, compared to Ni(110). In Figure 8b, the reaction orders of CO_2_ and H_2_ as a function of temperature are shown. The reaction order in CO_2_ is strongly positive due to the lack of carbonaceous species on the catalytic surface. The reaction order in CO_2_ appears to be inversely correlated to the trend in the apparent activation energy *E*_app_ (Figure 5b) of Ni(211). This further indicates that the activity is limited due to the absence of carbonaceous species. The orders in H_2_ are mildly positive since some H* remains present on the surface. The DRC analysis shown in Figure 8c reveals three rate-controlling steps. At lower temperatures, the CO* dissociation toward C* is prominent. The trend of this graph corresponds to the trend observed in *E*_app_ (Figure 5b), indicating that the transition from an inactive to an active catalyst is hampered by CO* dissociation. From the potential energy diagram of the predominant reaction pathway at 640 K depicted in Figure 8e, it can be seen that the direct CO dissociation has the highest forward activation barrier. Thus, a rate-controlling character can be expected. At higher temperatures, this elementary reaction becomes noncritical (DRC coefficient of 0).^32^ CO_2_* hydrogenation to COOH* becomes strongly rate-controlling with increasing temperature. This is caused by an initial imbalance in the rates for primary and secondary C–O bond scissions as a function of temperature. At a low temperature, there is insufficient thermal energy for secondary C–O bond scission. Hence, the generation of this surface intermediate, here via COOH* formation and COOH* dissociation to form CO* and OH*, leads to the buildup in CO* coverage. At elevated temperatures, the rate for direct CO* dissociation rapidly increases and only CO_2_* hydrogenation to COOH* remains to be a significant rate-controlling step. This is fully in line with the most dominant pathway, as shown in Figure 8d, where CO_2_* dissociation mainly takes place via COOH* and the dissociation of CO* proceeds via the carbide pathway.

#### 3.2.4 Ni(110)

**Figure 9:**
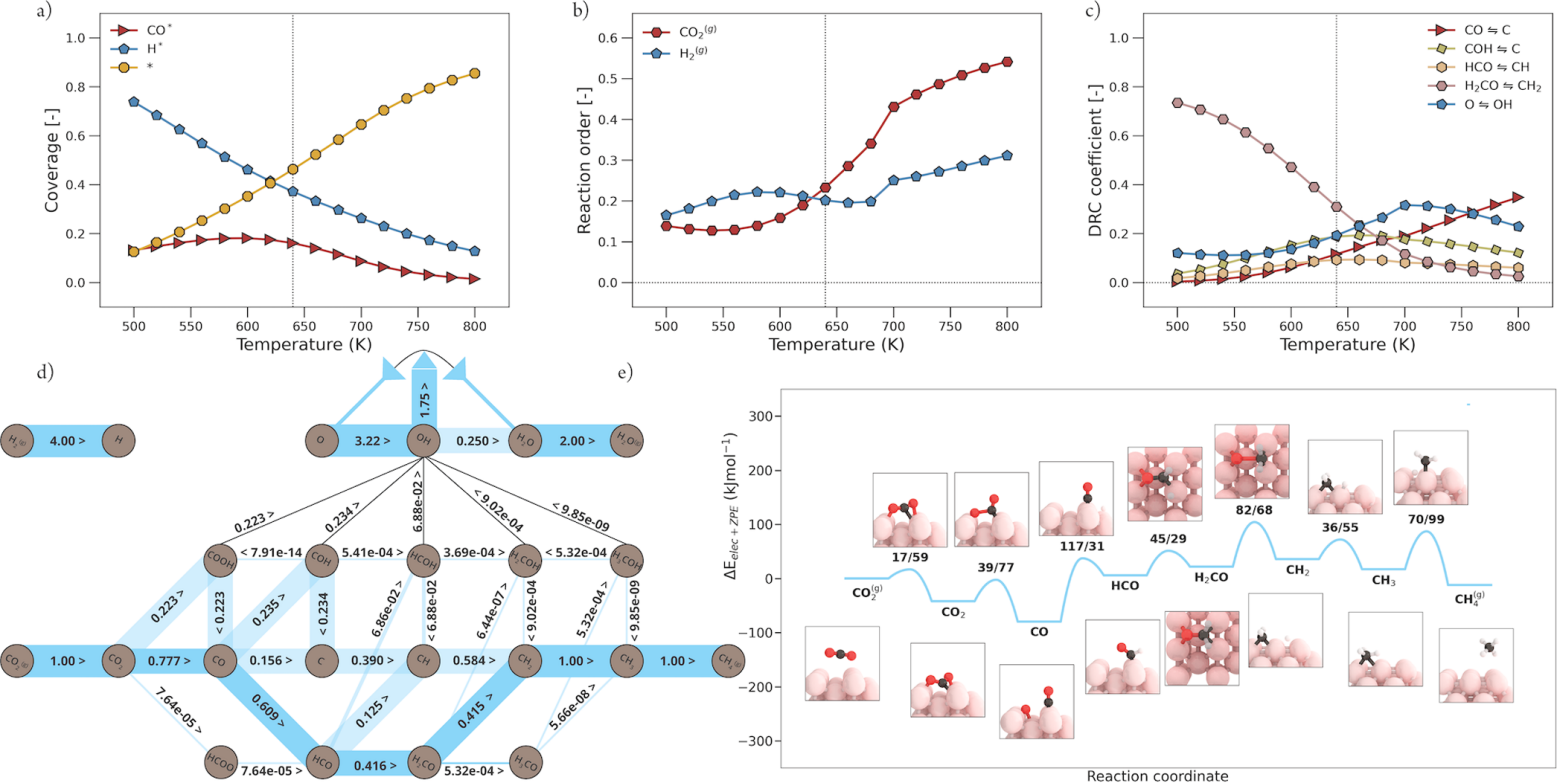
Micro-kinetics modeling (MKM) simulations of CO_2_ methanation over Ni(110). Explanation of the visual style is further given in Figure 6. (a) Surface coverages as a function of temperature. (b) Reaction order. (c) Elementary reaction steps with their significant degree of rate control (DRC) coefficients. (d) Flux diagram at 640 K and 1 bar. Bars of elementary reaction steps with significant—but not the largest—flux are transparent. (e) Potential energy diagram with geometry visualizations of the reaction pathway corresponding to the largest flux, as shown in panel (d). See Section G of the Supporting Information for the PED of the other significant pathways.

Ni(110) shows the highest activity in CO_2_ methanation compared to the other three facets (Figure 5a). Similar to Ni(111) and Ni(211), also this facet is covered both by CO* and H* (Figure 9a). With an increase in temperature, the rate for CO* activation increases, resulting in a decrease of CO* and H* coverage, which causes an increase in vacant sites. The reaction orders in CO_2_ and H_2_ for Ni(110) are shown in Figure 9b. The orders of both reactants are mildly positive, with a higher order for H_2_ compared to CO_2_, in agreement with experimental results obtained over Ni/SiO_2_ at 600 K in the literature.^15^ This experimentally based evidence gives us another reason to assume that the experimentally measured activity is likely to be caused by the presence and activity of Ni(110). The result of the DRC analysis is depicted in Figure 9c. Four elementary reaction steps with a significant DRC coefficient are observed for the secondary C–O bond cleavage. This indicates that the reaction rates of these elementary reactions are within the same order of magnitude, which can be further substantiated using the flux analysis at 640 K and the potential energy diagram corresponding to the predominant reaction flux, as shown in Figure 9d,e. The initial C–O bond scission of CO_2_* mainly occurs via a direct C–O dissociation belonging to the carbide pathway. However, the dissociation via COOH* from the carboxylic pathway is significant as well. For the second C–O dissociation, each primary reaction pathway is accessible with a comparable significant flux, which is in contrast to the other evaluated facets where only one of the primary pathways is predominantly taking place. The fundamental reason for accessibility of each pathway lies in the fact that the corresponding activation energies all have the same order of magnitude, as can be seen in Figure 4. Therefore, each of these elementary reaction steps show a comparable DRC coefficient. Among these reaction steps, H_2_CO* dissociation toward CH_2_* and O* has the smallest forward activation barrier, which results in the highest DRC coefficient up to 650 K. Thus, to improve the catalytic activity of this most active facet, investigations need to be conducted in search of supports or promotors to speed up this elementary reaction step.

Animations of the predominant reaction mechanism per nickel facet are made available in the Supporting Information.

### 3.3 Wulff Constructions

Ni-catalyzed CO_2_ hydrogenation is a structure-sensitive reaction,^9−11,13,14^ and thus it is expected that the surface density of particular active sites, as represented by the four facets studied, critically influences the activity and selectivity of the reaction. To understand the relation between nanoparticle size and the TOF toward methane production, a set of nickel metal nanoparticles in the range of 1.0–9.5 nm was synthesized in silico by means of a Wulff construction.

**Figure 10:**
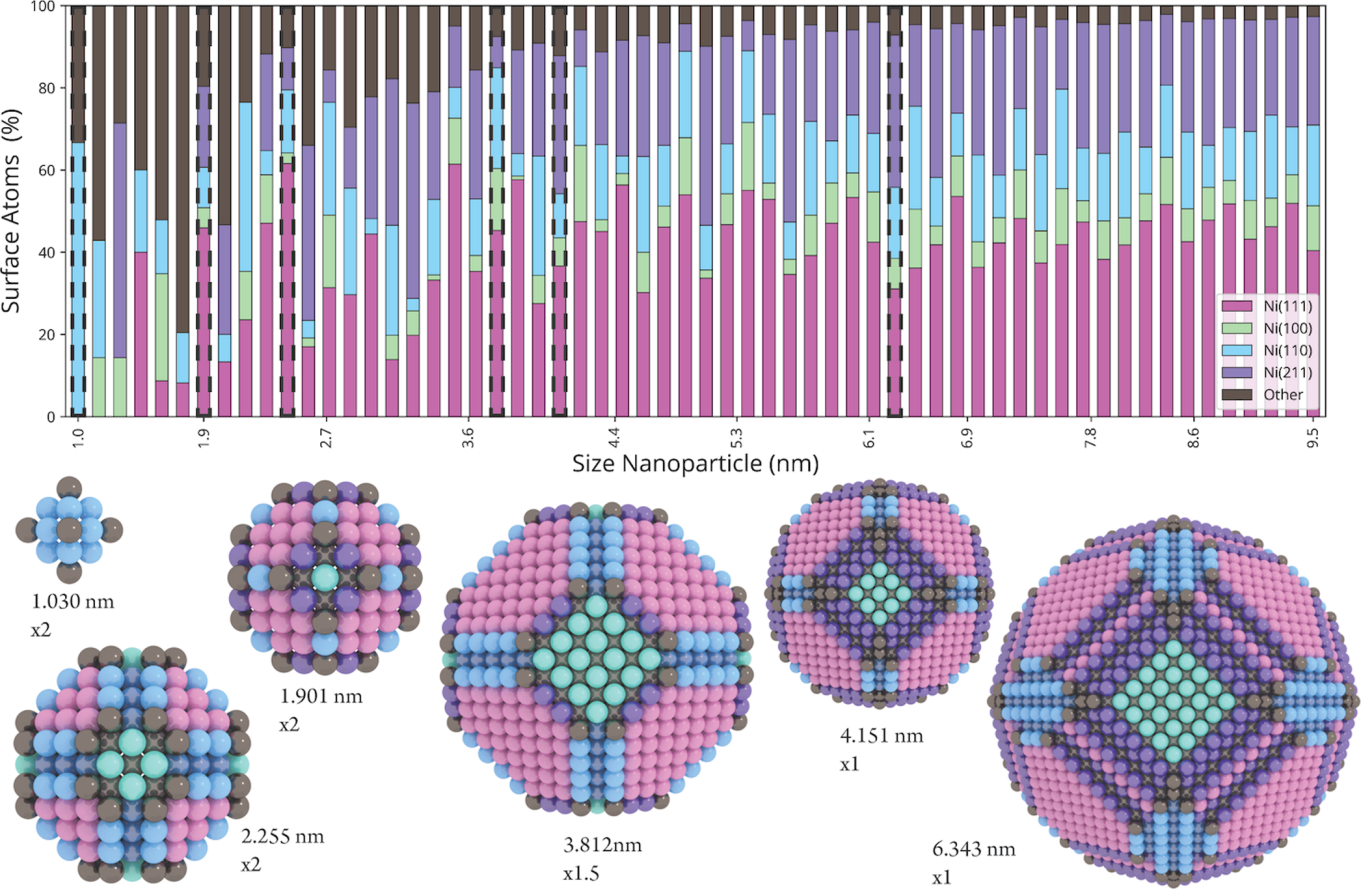
Bar chart depicts the fractional contribution of four nickel facets on Wulff-constructed metal nanoparticles, based on the common neighbor analysis (CNA) signature of each surface atom (for scatter and line plots, see Section K of the Supporting Information). Undercoordinated surface atoms with CNA signatures that do not belong to one of the four nickel facets were marked as Other. Visualizations of a selection of six Wulff-constructed nickel metal nanoparticles corresponding to six bars with a dotted edge. The colors of the surface atoms correspond to their CNA signature. Their relative size is indicated with a magnification factor.

To enumerate the active sites on these nanoparticles, an atomic pattern recognition algorithm based on the common neighbor analysis method was used.^46^ Fortunately, atomic fingerprints of each of the studied surface facets were found to be unique. This allows us to efficiently determine the surface density of the different types of active sites as a function of the nanoparticle size, the result of which is depicted in a bar chart in Figure 10 and in scatter- and line plots in Section K of the Supporting Information. Some atomic configurations are not part of any of the four studied facets and these are grayed out and labeled as “Other” in Figure 10. Unrecognized atomic configurations of atoms on the nanoparticle are commonly encountered for small nanoparticles, which have relatively irregular (i.e., nonidealized) topologies, but with increasing nanoparticle size, the contribution of such clusters becomes negligible. The surface fraction attributed to each of the four facets does not evolve in the same manner with an increase in the nanoparticle size. In general, the smaller metal nanoparticles have a larger amount of stepped nickel facets compared to terraces, while for the larger metal nanoparticles the opposite is true. The increase of Ni(111) is most prominent and increases roughly from 20% up to 45% with an increase in metal nanoparticle size. Even though less pronounced, a comparable trend is seen for the stepped surface Ni(211), which increases up to 30%. For the most active facet, Ni(110), the relative amount slightly decreases with an increase in the nanoparticle size and remains stable at 15%. The amount of Ni(100) is around 10% and barely changes throughout the size range. All in all, this indicates that the surface fraction of the most active facet, Ni(110), together with that of undercoordinated atoms becomes less pronounced with an increase in the nanoparticle size. The question now is what the effect would be on the overall activity for the complete Wulff-constructed metal nanoparticles with an increase in the nanoparticle size. For this, we constructed an activity plot, but we will first discuss some restrictions involved in this method.

Three limiting factors comparing theoretical and experimental activity plots need to be taken into account. First, in this study, we use a slab model approach where surface properties are optimized using a sufficient number of bulk atoms. A decrease in the number of bulk atoms will change the electronic behavior of surface atoms and consequently a change in the catalytic behavior can be expected. The Wulff-constructed nanoparticles of 1.0 and 1.2 nm only have one bulk atom, which makes the use of a slab model approach insufficient for these particles. Therefore, although we have examined them, for further analysis, we do not take into account the nanoparticles with only one bulk atom. Also, because of the slab model approach, we do not have information on the kinetic response of undercoordinated atoms, of which a higher fraction is only present for the smallest nanoparticles. However, due to geometrical resemblance to the atomic configuration of the stepped facets, we assume that their activity is close to the activity of the two most active facets, i.e., Ni(110) and Ni(211). Using a first-order approximation, we have estimated the activity of these undercoordinated atoms. An overview of the first-order approximation as well as an overview of Wulff constructions with their size, number of bulk atoms, and corresponding partition of each facet can be found in Section K of the Supporting Information. Second, for the theoretical activity plots, quantum effects become increasingly more dominant for Wulff-constructed nanoparticles smaller than ∼1.5 nm. This means that there is a somewhat higher uncertainty in the theoretical TOF plot for the smallest Wulff-constructed nanoparticles.

Lastly, the main difference between the construction of a TOF plot based on experiments on the one hand and on Wulff constructions on the other hand, lies in particle size distribution. The size of the nanoparticles that are experimentally measured can only be obtained with an average size dispersion. In contrast, the Wulff-constructed nanoparticles are very constrained and their size can be exactly determined. Also, the range of the constructed nanoparticles is very granular, which is inherent to the method of Wulff constructions. Hence, theoretically, we can assign a specific TOF value to a specific size of nanoparticle, while experimentally, an average TOF will be assigned to nanoparticles with a certain size distribution. To bridge this gap, we introduced mathematically a particle size distribution by taking a polynomial over the moving average of the theoretical data; see Section K of the Supporting Information for an overview of the applied techniques.

**Figure 11:**
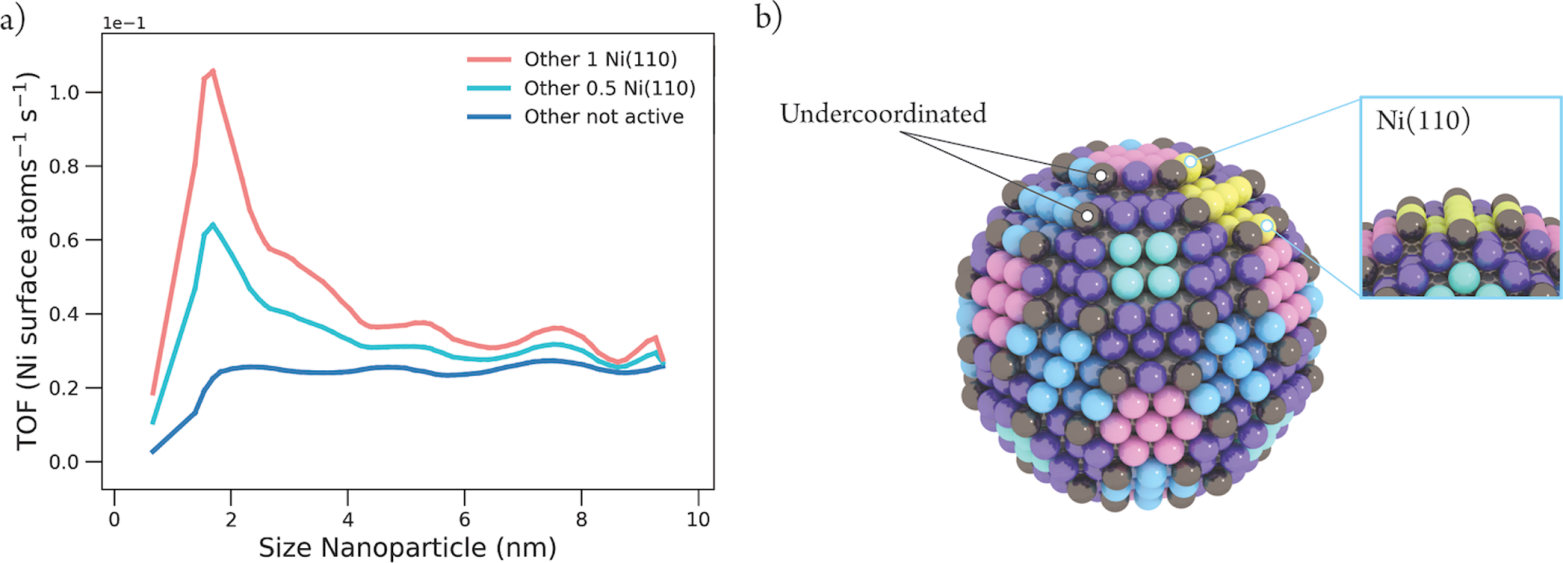
(a) Theoretical turn-over-frequencies (TOF) (Ni-surface atoms^–1^ s^–1^) plotted as a function of the size of a Wulff-constructed nickel metal nanoparticle. The activity of the undercoordinated atoms (Other) was assigned with no activity (blue), 0.5 times, and 1 time the activity of the most active facet Ni(110) (cyan and pink, respectively). A moving average with window 2 was applied and the curve was smoothened with a polynomial order 10. (b) Visualization of a Wulff-constructed nickel metal nanoparticle of 3.008 nm size, where undercoordinated atoms (Other) are shown in gray, Ni(111) is shown in pink, Ni(100) in green, Ni(211) in purple, and Ni(110) in blue. On the right, the area of Ni(110) is highlighted in yellow, representing the proposed most active site for catalytic CO_2_ hydrogenation.

The resulting theoretical total activity plots for catalytic CO_2_ methanation over nickel metal nanoparticles at 640 K are shown in Figure 11a, where the activity of the undercoordinated atoms was assigned with no activity (blue), half of the activity (cyan), and 1 time the activity (pink) of the most active facet, Ni(110). It is apparent that the characteristic peak starts to appear around 2 nm when the undercoordinated atoms are assigned with a significant amount of activity (cyan, pink). The shape of these TOF plots is in satisfactory agreement with the experimentally obtained TOF plot (Figure 1a). However, the maximum TOF values are 0.064 and 0.106 CO_2_-converted Ni-surface atom^–1^ s^–1^ (cyan and pink, respectively). Even though this is 2–4 times higher than the experimentally measured activity for Ni/SiO_2_ catalysts (Figure 1a), it is well within the same order of magnitude. The TOF plot where the activity of the undercoordinated atoms was set at zero (blue) shows an increase in activity with an increase in the nanoparticle size up to 2 nm, after which it reaches a plateau with a maximum activity of 0.025 CO_2_ converted Ni-surface atom^–1^ s^–1^. This value is in excellent agreement with the maximum TOF determined experimentally (Figure 1a). However, the shape of this plot is missing the characteristic peak around 2 nm. Without this characteristic peak, the shape of this plot resembles the characteristics of a TOF plot for CO hydrogenation over cobalt nanoparticles in a Fischer–Tropsch reaction, which typically shows an increase in activity with an increase in the nanoparticle size up to 6–8 nm after which it reaches a plateau.^57,58^

Plotted TOF for each facet can be found in Section K of the Supporting Information. From this, it is evident that the activity of Ni(110) dominates over the other facets and, together with the undercoordinated atoms, dictates the overall trend in the total activity of the nanoparticles. Even though the other stepped facet, Ni(211), is the second most active facet, its activity contributes barely to the overall activity. The activities of the terraces, Ni(111) and Ni(100), do not contribute significantly to the total activity even though half of the surface of the larger nickel metal particles consists out of these facets. This is not surprising since flat surfaces are known to have difficulty in dissociating a π-bond such as that of CO and CO_2_.^12,44^^12,44^ The overall trend between TOF and nanoparticle size shown in Figure 11a, where undercoordinated atoms are assigned with significant activity, corresponds to the trends observed in the experiments (e.g., Figure 1a for Ni/SiO_2_ catalysts),^9−11^ that is a steep increase in TOF up to ∼2 nm, a low activity for the larger particles, and an optimum in CO_2_ methanation for nickel nanoparticles of 2–3 nm. This implies that, in addition to the four chosen nickel metal facets from this study, undercoordinated atoms are also needed to get a good representative model of nickel metal nanoparticles and study the structure sensitivity of CO_2_ methanation. Future studies will have to focus on what the effect is of the metal nanoparticle–support interfacial structures, especially for reducible support oxides (e.g., Ni/TiO_2_ catalysts), where the activity was shown to be much improved compared to Ni/SiO_2_.^9,59^ Another interesting aspect for future research is to unravel the intrinsic kinetic response together with the reaction flux and degree of rate control for undercoordinated atoms of nickel metal nanoparticles in the CO_2_ methanation reaction.

One can now start to envisage what the most active nickel metal nanoparticle for catalytic CO_2_ hydrogenation would look like, taking the Taylor view of the concept of active sites into account.^14^Figure 11b shows a “static” view of a 3 nm sized Wulff-constructed nickel metal particle, where the yellow surface atoms represent the most active surface structure for catalytic CO_2_ hydrogenation. It should be clear that this is a “static” view, while it has been shown that nanoparticles are more “dynamic” in their shape and size during catalytic operation.^11,60,61^

## 4 Conclusions

This work provides new insights into the structure-sensitive nature of the catalytic hydrogenation of CO_2_ to CH_4_, which is better known as the Sabatier reaction. A combination of density functional theory (DFT) calculations and micro-kinetics modeling (MKM) simulations was employed on an extended reaction network to unravel the predominant reaction pathway of CO_2_ methanation over Ni(111), Ni(100), Ni(110), and Ni(211) surfaces.

Based on the MKM simulations, the two terraces, Ni(111) and Ni(100), barely show any activity in CO_2_ methanation. Crucial elementary reaction steps for Ni(111) at reaction temperatures are the formation of CO* after the first C–O bond scission and the removal of CO* via HCO* dissociation, both identified as equally rate-controlling. Ni(100) suffers severely from C* poisoning due to its high stability in the fourfold sites. The stepped Ni(211) facet is second most active; however, its activity is 3 orders of magnitude lower compared to Ni(110). The predominant reaction mechanism on Ni(211) is identified via the carbide pathway, but with CO_2_* dissociation via the carboxyl intermediate, i.e., COOH*. Ni(110) shows the highest activity in CO_2_ methanation compared to the other studied facets with a comparable TOF to values reported in the literature.^9−11^ The reaction flux shows that CO_2_* activation goes both via the carbide and carboxylic pathway and each primary pathway is energetically accessible, considering CO* dissociation. This optimally facilitates CO_2_ methanation.

With the combination of a common neighbor analysis (CNA) approach, MKM simulations, and first-order approximations, theoretical TOF plots were constructed for a range of Wulff-constructed nickel metal nanoparticles. The main characteristics observed for this structure-sensitive reaction are covered, despite the limitations of Wulff constructions. The maximum activity of the theoretical activity plots is in close agreement with the experimentally observed activity. With the kinetic response of the four nickel facets, the theoretical TOF plot shows a plateau for nanoparticles larger than 2 nm. The characteristic peak in activity around 2 nm for nickel nanoparticles in CO_2_ methanation was obtained only when the undercoordinated atoms were assigned with a significant kinetic response. The optimal activity for the Wulff-constructed nickel metal nanoparticles was found to be 1.7 nm and the larger particles show a decrease in activity. The trend in the TOF plot is mainly dictated by the relative amount of Ni(110) as well as the undercoordinated atoms on the outer surface and corresponds to the experimentally observed structure-sensitive relation between nanoparticle size and catalytic activity.

## Supporting Information Available

Notes on the choice of the pseudopotential, numerical approach, lists of calculated adsorption energies including references, visualization of stable geometries of reaction intermediates as well as transition states, potential energy diagrams, full overview of DRC coefficients, input files of applied micro-kinetics modeling simulations, sensitivity analysis on the correction of CO* overbinding, sensitivity analysis on the lateral interaction potential, details of the Wulff-constructed nickel metal nanoparticles with the calculation of their activity in CO_2_ methanation, and animations of the predominant reaction mechanisms per nickel facet (PDF)
Animation of the reaction pathway with the highest flux per nickel facet, corresponding to the main flux depicted in Figure 6d (MP4)
Animation of the reaction pathway with the highest flux per nickel facet, corresponding to the main flux depicted in Figure 7d (MP4)
Animation of the reaction pathway with the highest flux per nickel facet, corresponding to the main flux depicted in Figure 8d (MP4)
Animation of the reaction pathway with the highest flux per nickel facet, corresponding to the main flux depicted in Figure 9d (MP4)

## Notes

This work has been mainly performed by E.B.S. as part of her Ph.D. thesis work.

## Abbreviations

CNA: common neighbor analysis
DFT: density functional theory
DRC: degree of rate control
fcc: face-centered cubic
hcp: hexagonal close-packed
MKM: micro-kinetics modeling
P2G: power-to-gas
PAW: projector-augmented wave
PBE: Perdew–Burke–Ernzerhof
PtM: power-to-methane
SC: single crystal
VASP: Vienna Ab initio Simulation Package
