# Supplementary material for: Structure Sensitivity of CO2 Conversion over Nickel Metal Nanoparticles Explained by Micro-Kinetics Simulations
Source: JACS Au. 2022 Oct 14;2(12):2714–30. doi: 10.1021/jacsau.2c00430 (PMC9795468; doi:10.1021/jacsau.2c00430)
Supplement: Supplementary file 1 — au2c00430_si_001.pdf [file au2c00430_si_001.pdf]

Supporting Information: Structure Sensitivity of CO_2 Conversion over Nickel Metal Nanoparticles Explained by Micro-Kinetics Simulations

Ellen B. Sterk[a], Anne-Eva Nieuwelink[a], M. Monai[a], Jaap N. Louwen[a], Eelco T.C. Vogt[a], Ivo A.W. Filot[b,*] and Bert M. Weckhuysen[a,*]

[a] *Inorganic Chemistry and Catalysis group, Debye Institute for Nanomaterials Science, Utrecht University, Universiteitsweg 99, 3584 CG Utrecht, The Netherlands*

[b] *Schuit Institute of Catalysis, Department of Chemical Engineering and Chemistry, Eindhoven University of Technology, PO Box 513 5600 MB Eindhoven, The Netherlands*

*Email: I.A.W.Filot@tue.nl, B.M.Weckhuysen@uu.nl

Table of Contents

A	Hard or soft pseudopotential	3 - 4
B	Numerical approach	5
C	References adsorption energies	6 - 9
D	Stable geometries of reaction intermediates	10 - 28
E	Table adsorption energy of stable geometries	29 - 33
F	Geometries of elementary reaction steps	34 - 42
G	Potential energy diagrams	
	Primary pathways	43 - 45
	Reaction pathway based on flux diagrams	46 - 56
H	Microkinetics simulations	
	Input	57 - 62
	Output	63 – 65
I	Sensitivity analysis on the correction of CO^* overbinding	66 – 74
J	Sensitivity analysis on the lateral interaction potential	75 – 79
K	Wulff constructions	
	Wulff constructed nanoparticles	80 – 81
	Turnover frequency	82 - 84

A Hard or Soft Pseudopotential

In this work we used pseudopotentials for Ni, H, C and O atoms. In order to be able to compare results within the obtained data set we need to use the same settings for each calculation. This means that each calculation needs to be performed with the same ENCUT value, determined by the highest default cutoff (or ENMAX) of the atoms Ni, H, C and O. For this set of atoms, the highest default cutoff belongs to oxygen and carbon for both the soft and the hard pseudopotentials. An increase in the cutoff energy generally results in an increase in computational costs, thus this needs to be chosen carefully.

On the VASP site[1], an important note is made for the usage of PAW pseudopotentials when one needs to calculate dimers with short bonds. As this is the case for the slabs with CO^* adsorbed, we investigated the energetic effect of the hard- and soft PAW-PBE potential. For the hard variant an ENCUT of 700 eV is required, while for the soft one an ENCUT of 400 eV is sufficient.

The adsorption energies of CO^* in the most- and least stable adsorption site for each investigated nickel facet, are listed in Table S 1 below. The energies calculated with the hard pseudopotential are higher compared to the soft pseudopotential, except for CO^* adsorbed atop on Ni(100). In each case the energy difference is within 10 kJ/mol.

Table S 1. Comparison of adsorption energies calculated with hard- and soft- PAW-PBE pseudopotential.

**Adsorption site**	$\mathbf{E}_{\mathrm{PBE}}$	$\mathbf{E}_{\mathrm{PBE\_h}}$	$\Delta\mathrm{E}_{\mathrm{PBE-PBE\_h}}$
Ni(111) T	-144.38	-136.83	7.55
Ni(111) $\mathrm{T_h}$	-178.65	-169.08	9.57
Ni(100) T	-147.65	-150.37	-2.72
Ni(100) F	-174.93	-173.21	1.72
Ni(110) T	-157.35	-152.15	5.20
Ni(110) B1	-174.60	-167.47	7.15
Ni(211) $\mathrm{T_1}$	-165.05	-158.45	6.60
Ni(211) $\mathrm{T_h^2}$	-186.79	-176.54	10.25

Adsorption energies are ZPE-corrected. Values are given in kJ/mol.

To put this in perspective, we have compared gas phase reaction energies for some relevant reactions by using molecular energies computed by the PBE DFT and with CCSD(T) method (the "gold standard" in computational chemistry), in both cases with a 6-311+G(3df,2pd) basis set. These values, listed in Table S 2, were taken from the NIST Computational Chemistry Comparison and Benchmark DataBase[2].

[1] VASP. Available PAW potentials. https://www.vasp.at/wiki/index.php/Available_PAW_potentials (accessed March 31, 2022).
[2] Computational Chemistry Comparison and Benchmark DataBase. https://cccbdb.nist.gov (accessed March 31, 2022).

A. Hard or Soft Pseudopotential

Table S 2. Gas phase reaction energies of relevant reactions.

Reaction	$E_{reaction}$ PBE	$E_{reaction}$ CCSD(T)	$\|\Delta\|$
$CO_2 + H_2 \longrightarrow CO + H_2O$	76.5	31.6	44.9
$CO + H_2 \longrightarrow H_2CO$	-52.8	-21.8	31.0
$CO_2 + 4\ H_2 \longrightarrow 2\ H_2O + CH_4$	-222.6	-239.1	16.4
$H_2CO + 2\ H_2 \longrightarrow 2\ H_2O + CH_4$	-246.3	-248.9	2.6
$CO + 3\ H_2 \longrightarrow H_2O + CH_4$	-299.1	-270.7	28.5

All energies are given in kJ/mol

Approximations need to be balanced in order to avoid wasting computer time. Therefore, in view of the deviations in the calculated thermochemistry of CO_2 methanation between PBE and CCSD(T), the usage of the soft PAW-PBE potential throughout this work is justified.

B Numerical Approach

We also did a limited study to gauge the sensitivity of our results to the model and numerical approach chosen.
PAW calculations were done with the GPAW program[3,4,5] version 21.6.0 using the supplied PAW setups (version 0.9.2000), a plane wave cutoff energy of 500 eV and the PBE density functional. The Ni(111) surface was modeled by four layers of 4 by 4 Ni atoms. The bulk unit cell size of FCC Ni was computed to be 3.5291Å, which leads to unit cell axes of length 9.9818 Å along the slab. The length of the axis perpendicular to the slab was set to 26.1126 Å to have 10 Å of vacuum to both sides if the slab. Periodic boundary conditions were applied for the two axes along the slab; no periodic boundary conditions were applied perpendicular to the slab. A Monkhorst-Pack 4x4x1 k-point mesh was used for sampling the Brillouin zone.
CO was placed on one side of the slab in T, B and Tf coordination mode. We compared the adsorption energy, computed as $E_{\text{adsorption}} = E_{\text{slab+CO}} - E_{\text{slab}} - E_{\text{CO}}$ and compared these values to those derived from the VASP calculations (see paper for details).
Obtained values are reported in Table S 3. Despite the difference in atomic model, the difference in software and the difference in numerical parameters the results are very close together. We conclude that our results are not critically dependent on the choices made.

Table S 3. CO adsorption energy on Ni(111).

Adsorption site	Adsorption energy	
	VASP	GPAW
T	-144.3	-147.4
B	-158.8	-165.3
Tf	-178.1	-174.8

Values are given in kJ/mol.

[3] Mortensen, J. J.; Hansen, L. B.; Jacobsen, K. W. A Real-Space Grid Implementation of the Projector Augmented Wave Method. Phys. Rev. B - Condens. Matter Mater. Phys. **2004**, 71 (3), 1–11. https://doi.org/10.1103/PhysRevB.71.035109.
[4] Enkovaara, J.; Rostgaard, C.; Mortensen, J. J.; Chen, J.; Dułak, M.; Ferrighi, L.; Gavnholt, J.; Glinsvad, C.; Haikola, V.; Hansen, H. A.; Kristoffersen, H. H.; Kuisma, M.; Larsen, A. H.; Lehtovaara, L.; Ljungberg, M.; Lopez-Acevedo, O.; Moses, P. G.; Ojanen, J.; Olsen, T.; Petzold, V.; Romero, N. A.; Stausholm-Møller, J.; Strange, M.; Tritsaris, G. A.; Vanin, M.; Walter, M.; Hammer, B.; Häkkinen, H.; Madsen, G. K. H.; Nieminen, R. M.; Nørskov, J. K.; Puska, M.; Rantala, T. T.; Schiøtz, J.; Thygesen, K. S.; Jacobsen, K. W. Electronic Structure Calculations with GPAW: A Real-Space Implementation of the Projector Augmented-Wave Method. J. Phys. Condens. Matter **2010**, 22 (25), 253202. https://doi.org/10.1088/0953-8984/22/25/253202.
[5] Hjorth Larsen, A.; JØrgen Mortensen, J.; Blomqvist, J.; Castelli, I. E.; Christensen, R.; Dułak, M.; Friis, J.; Groves, M. N.; Hammer, B.; Hargus, C.; Hermes, E. D.; Jennings, P. C.; Bjerre Jensen, P.; Kermode, J.; Kitchin, J. R.; Leonhard Kolsbjerg, E.; Kubal, J.; Kaasbjerg, K.; Lysgaard, S.; Bergmann Maronsson, J.; Maxson, T.; Olsen, T.; Pastewka, L.; Peterson, A.; Rostgaard, C.; SchiØtz, J.; Schütt, O.; Strange, M.; Thygesen, K. S.; Vegge, T.; Vilhelmsen, L.; Walter, M.; Zeng, Z.; Jacobsen, K. W. The Atomic Simulation Environment - A Python Library for Working with Atoms. J. Phys. Condens. Matter **2017**, 29 (27). https://doi.org/10.1088/1361-648X/aa680e.

C References Adsorption Energies

Most stable calculated adsorption energies (in kJ/mol) of relevant reaction intermediates involved in CO_2 methanation on Ni(100), Ni(110), Ni(111) and Ni(211) are compared to values found in literature in the four tables below (Table S 4 to Table S 7). Corresponding adsorption sites are given within brackets.

Table S 4. References in adsorption energy on Ni(100).

	Ni(100)		
**Adsorbate**	E_{ads}	**Reference**	
CO_2	-16.3 (BTT)	-5.79 (T)	[1]
		-13.51 (BTT)	[2]
CO	-174.8 (F)	-189.11 (F)	[1]
		-196.83 (F)	[2]
		-219.02 (F)	[3]
CH_3	-233.8 (B)	-184.29 (B)	[3]
HCOO	-293.8 (TT over F)	-277.88 (TB)	[1]
HCO	-264.6 (BB)	-266.30 (BB)	[1]
		-303.93 (BB)	[3]
H_3CO	-251.9 (F)	-260.51 (F)	[3]
COOH	-232.9 (TT)	-260.51 (B)	[1]
H_3COH	-29.0 (T)	-25.48 (T)	[4]
OH	-388.4 (F)	-318.40 (F)	[1]
		-324.54 (F)	[3]
H_2O	-31.1 (T)	-26.05 (T)	[1]
H	-364.5 (F)	-260.51 (F)	[1]
		-273.05 (F)	[3]
O	-724.2 (F)	-542.25 (F)	[1]
		-525.84 (F)	[2]
H_2	-1 (horizontal above T)	-21.23 (T)	[1]

Table S 5. References in adsorption energy on Ni(110).

	Ni(110)		
**Adsorbate**	E_{ads}	**Reference**	
CO_2	-41.9 ($T_1T_1T_1$)	-24.12 (B_1)	[1]
		-40.52 ($T_1T_1T_1$)	[2]
CO	-174.5 (B_1)	-182.36 (B_1)	[1]
		-187.18 (B_1)	[2]
HCOO	-327.8 (T_1T_1 over lower edge)	-317.44 (TT)	[1]
HCO	-239.6 (T_fB_1)	-233.49 (B_1T_1)	[1]
COOH	-248.1 (T_1T_1 over lower edge)	-246.04 (B_2)	[1]
OH	-398.6 (B_1)	-335.77 (B_1)	[1]
H_2O	-38.5 (T_1)	-38.59 (T_f)	[1]
H	-351.6 (B_2)	-242.18 (T_f)	[1]
O	-676.4 (T_f)	-479.53 (B_2)	[1]
		-467.95 (T_f)	[2]
H_2	-34.9 (horizontal above T_1)	-31.84 (T)	[1]

Table S 6. References in adsorption energy on Ni(111).

	Ni(111)		
**Adsorbate**	E_{ads}	**Reference**	
CO_2	24.3 (T_fTT)	-8.68 (BTT)	[6]
		-2.89 (B)	[1]
		29.91 (TT)	[2]
		-1.93 (T)	[7]
		-11.58 (TT)	[8]
CO	-178.6 (T_h)	-127.36 (T_f)	[6]
		-182.36 (T_h)	[1]
		-184.29 (T_h)	[2]
		-190.08 (T_f)	[9]
		-171.74 (T_f)	[7]
		-201 (T_h)	[8]
C	-773.8 (T_h)	-530.67 (T_f)	[6]
		-637.77 (T_h)	[8]
CH	-603.3 (T_f)	-543.21 (T_f)	[6]
		-659.96 (T_f)	[8]
CH_2	-454.2 (T_f)	-340.59 (T_f)	[6]
CH_3	-238.3 (T_f)	-127.36 (T_f)	[6]
		-196.83 (T_f)	[9]
HCOO	-277.3 (TT)	-163.06 (B)	[6]
		-259.55 (TT)	[1]
		-291.39 (TT)	[8]
HCO	-201.2 (BT over T_f)	-211.30 (BT over T_h)	[1]
		-232.53 (B)	[9]
		-240.25 (B)	[8]
H_2CO	-57.7 (T_fT)	-70.434 (B)	[6]
		-99.38 (TT)	[9]
H_3CO	-218.0 (T_h)	-249 (T_f)	[9]
COOH	-210.9 (BT over T_f)	-209.37 (T)	[1]
		-245.07 (BT)	[8]
COH	-410.5 (T_h)	-426.47 (Tf)	[8]
H_2COH	-146.7 (BT over T_h)	-162.10 (T)	[9]
H_3COH	-23.6 (T)	-14.47 (T)	[9]
OH	-364.9 (T_f)	-230.60 (T_f)	[6]
		-299.10 (T_f)	[1]
		-307 (T_f)	[9]
		-304.89 (T_f)	[7]
		-322.26 (T_f)	[8]
H_2O	-22.5 (T)	-49.21 (T)	[6]
		-18.33 (T)	[1]
		-26 (T)	[7]
		-45.35 (T)	[8]
H	-361.7 (T_f)	-295.25 (T_f)	[6]
		-255.69 (T_f)	[1]
		-284.63 (T_f)	[3]
		-267.26 (T_f)	[8]
O	-692.4 (T_f)	-466.02 (T_f)	[6]
		-511.37 (T_f)	[1]
		-489.18 (T_f)	[2]
		-507.51 (T_f)	[7]
		-464.09 (T_f)	[8]
H_2	38.8 (above B)	-69.47 (T_f)	[6]
		-16.40 (T)	[1]

Table S 7. References in adsorption energy on Ni(211).

	Ni(211)		
**Adsorbate**	E_{ads}	**Reference**	
CO_2	-39.1 ($B_1T_1T_1$)	-40.52 (B_1)	[5]
		-29.91 (TT)	[8]
CO	-186.7 (T_h^2)	-190 (T_h^2)	[10]
		-201.65 (T_h^1)	[8]
C	-874.1(F)	-719.78 (T_h^2)	[5]
		-759.34 (F)	[10]
		718.82 (F)	[8]
CH	-634.2 (F)	-589.53 (F)	[5]
		-650.31 (F)	[10]
		-677.33 (F)	[8]
CH_2	-467.8 (T_h^2)	-389.8 (B_1)	[5]
		-398.48 (T_h^2)	[10]
CH_3	-265.6 (T_h^2)	-176.57 (B_1)	[5]
		-212.27 (B_1)	[10]
HCOO	-340.6 (T_1T_1)	-329.01 (TT)	[8]
HCO	-248.2 ($T_f^1B_1$)	-241.21 (B_1)	[10]
		-243.14 (F)	[8]
H_2CO	-90.3 ($T_{2\text{-}3}B_1$)	-109.03 (B_1)	[10]
H_3CO	-269.1(B_1)	-279.81 (B_1)	[10]
COOH	-257.0 (B_1T_1)	-244.11 (TT)	[8]
COH	-415.6 (F)	-426.47 (T_h^2)	[10]
		-419.71 (T_h^2)	[8]
HCOH	-314.4 (B_1 OH down)	-411.03 (B_1)	[10]
H_2COH	-192.6 (B_1)	-203.58 (B_1)	[10]
H_3COH	-44.3 (T_1)	-60.79 (T_1)	[10]
OH	-415.0 (B_1 H over lower edge)	-372.43 (B_1)	[10]
		-334.80 (B_1)	[8]
H_2O	-48.5 (T_1 H's over lower edge)	-50.17 (T_1)	[10]
		-69.47 (T_1)	[8]
H	-359.2 (T_f^2)	-259.55 (T_h^2)	[5]
		-258.58 (T_h^2)	[10]
		-263.40 (T_h^2)	[8]
O	-711.4 (T_h^2)	-565.40 (T_h^2)	[10]
		-464.09 (T_h^2)	[8]
H_2	-11.4 (horizontal above T_1)	-17.37 (T_1)	[10]

C. References Adsorption Energies

References

(1) Mohsenzadeh, A.; Richards, T.; Bolton, K. DFT Study of the Water Gas Shift Reaction on Ni(111), Ni(100) and Ni(110) Surfaces. *Surf. Sci.* **2016**, *644*, 53–63. https://doi.org/10.1016/j.susc.2015.09.014.

(2) Wang, S.; Cao, D.; Li, Y.; Wang, J.; Jiao, H. Chemisorption of CO2 on Nickel Surfaces. *J. Phys. Chem. B* **2005**, *109*, 18956–18963.

(3) Zhou, Y. H.; Lv, P. H.; Wang, G. C. DFT Studies of Methanol Decomposition on Ni(1 0 0) Surface: Compared with Ni(1 1 1) Surface. *J. Mol. Catal. A Chem.* **2006**, *258* (1–2), 203–215. https://doi.org/10.1016/j.molcata.2006.04.013.

(4) Kresse, G.; Hafner, J. Ab Initio Molecular-Dynamics Simulation of the Liquid-Metal–Amorphous-Semiconductor Transition in Germanium. *Phys. Rev. B* **1994**, *49* (20), 14251–14269. https://doi.org/10.1103/PhysRevB.49.14251.

(5) Yang, K.; Zhang, M.; Yu, Y. Methane Formation from Successive Hydrogenation of C over Stepped Ni and Ni3Fe Surfaces: Effect of Surface Composition. *Int. J. Hydrogen Energy* **2017**, *42* (2), 914–927. https://doi.org/10.1016/j.ijhydene.2016.09.172.

(6) Ren, J.; Guo, H.; Yang, J.; Qin, Z.; Lin, J.; Li, Z. Insights into the Mechanisms of CO 2 Methanation on Ni(111) Surfaces by Density Functional Theory. *Appl. Surf. Sci.* **2015**, *351*, 504–516. https://doi.org/10.1016/j.apsusc.2015.05.173.

(7) Zhang, M.; Zijlstra, B.; Filot, I. A. W.; Li, F.; Wang, H.; Li, J.; Hensen, E. J. M. A Theoretical Study of the Reverse Water-Gas Shift Reaction on Ni(111) and Ni(311) Surfaces. *Can. J. Chem. Eng.* **2019**, No. August, 1–9. https://doi.org/10.1002/cjce.23655.

(8) Catapan, R. C.; Oliveira, A. A. M.; Chen, Y.; Vlachos, D. G. DFT Study of the Water-Gas Shift Reaction and Coke Formation on Ni(111) and Ni(211) Surfaces. *J. Phys. Chem. C* **2012**, *116* (38), 20281–20291. https://doi.org/10.1021/jp302488f.

(9) Wang, G. C.; Zhou, Y. H.; Morikawa, Y.; Nakamura, J.; Cai, Z. S.; Zhao, X. Z. Kinetic Mechanism of Methanol Decomposition on Ni(111) Surface: A Theoretical Study. *J. Phys. Chem. B* **2005**, *109* (25), 12431–12442. https://doi.org/10.1021/jp0463969.

(10) Zhi, C.; Zhang, R.; Wang, B. Comparative Studies about CO Methanation over Ni(211) and Zr-Modified Ni(211) Surfaces: Qualitative Insight into the Effect of Surface Structure and Composition. *Mol. Catal.* **2017**, *438*, 1–14. https://doi.org/10.1016/j.mcat.2017.05.012.

D Stable Geometries of Reaction Intermediates

In Figure S 2 to Figure S 21 show top and side view of stable geometries on Ni(111), Ni(100), Ni(110) and Ni(211) calculated for each adsorbate of the carbide, formate, carboxylic pathway and water formation. The stable geometries are ordered from left to right in descending stability. Corresponding chemisorption energy (kJ/mol) and adsorption site are indicated above.

Ni(111)

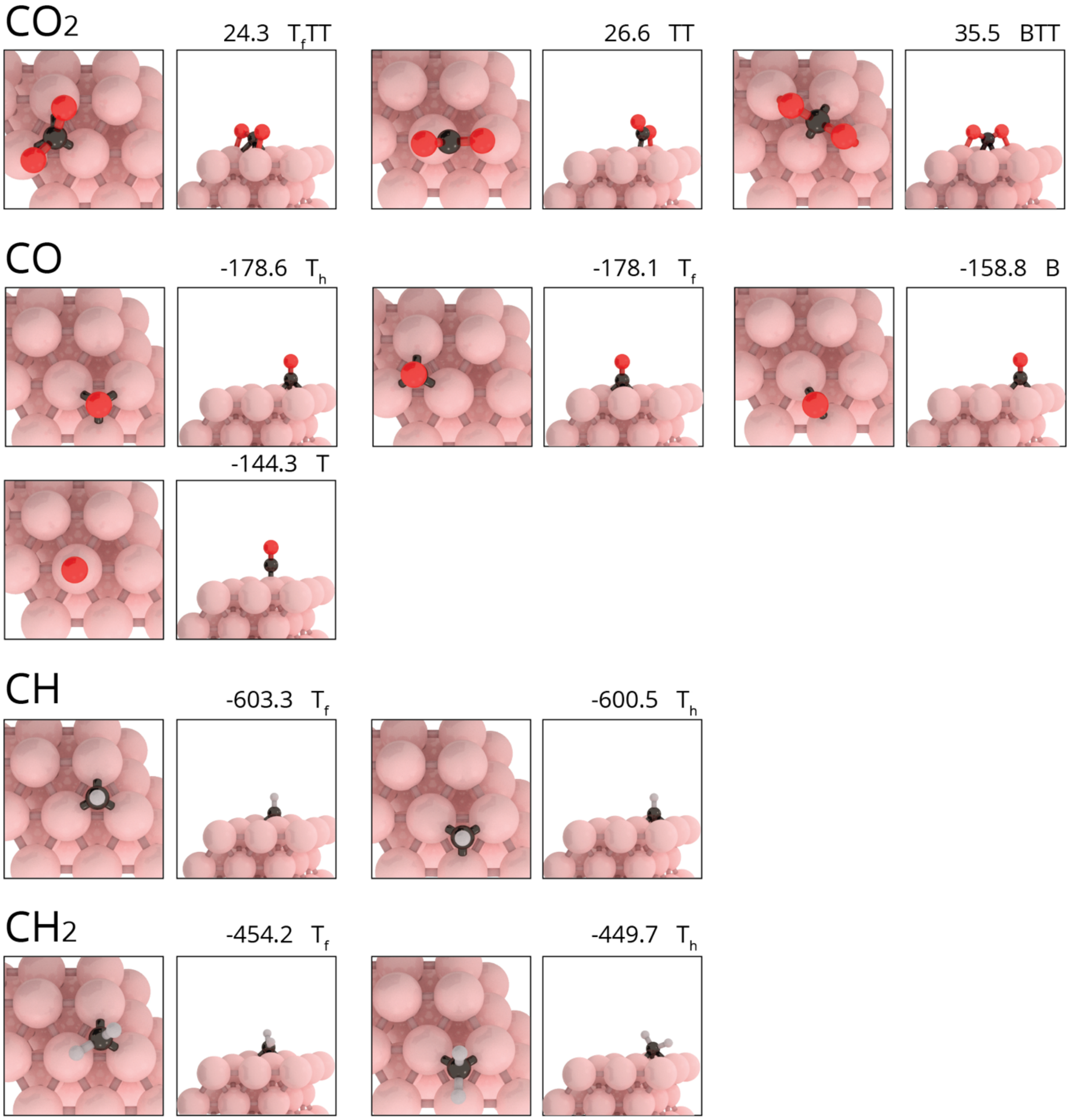

Figure S 1. Top and side view of stable geometries for CO_2, CO, CH and CH_2 on Ni(111). Corresponding chemisorption energy (kJ/mol) and adsorption site is indicated above each picture frame.

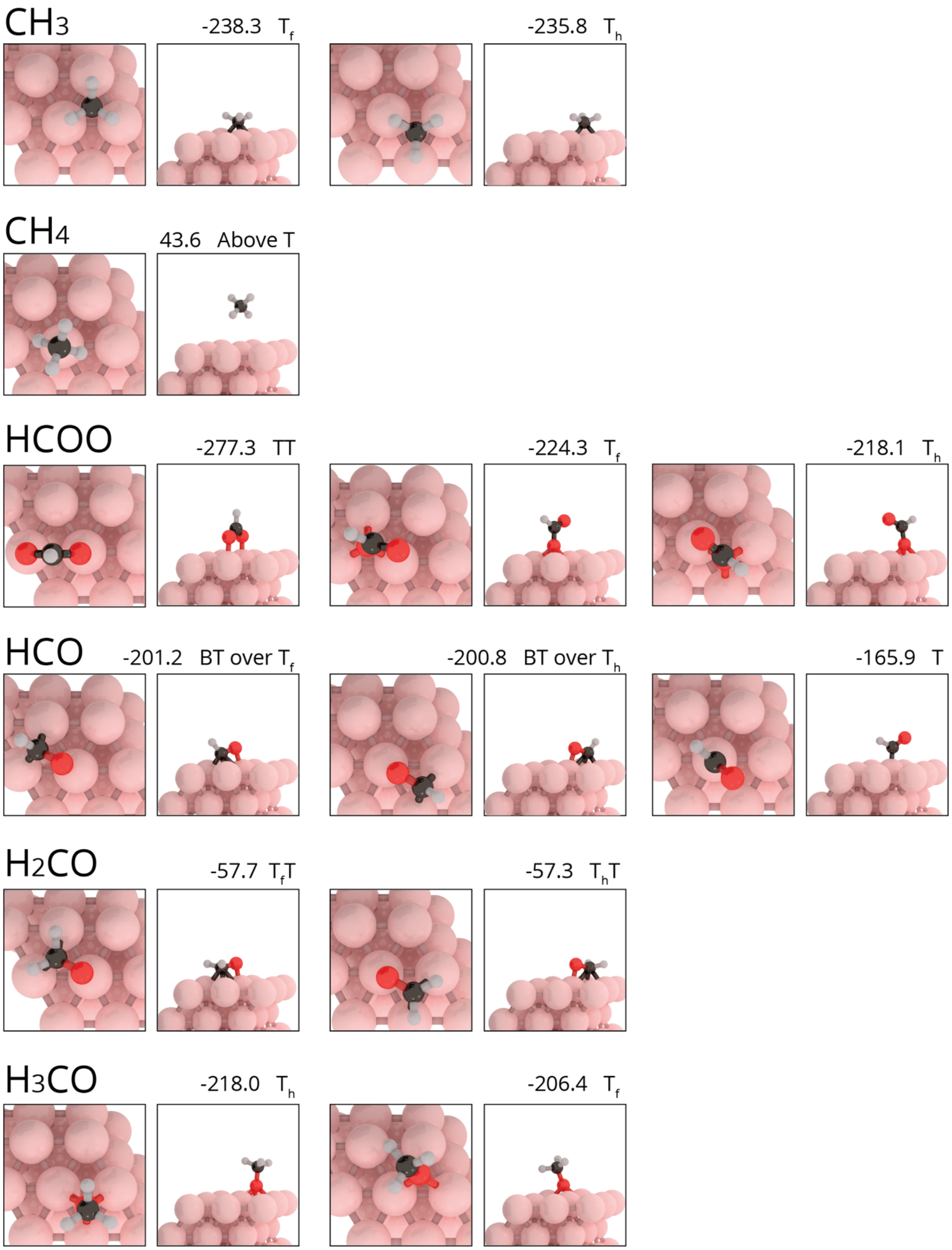

Figure S 2. Top and side view of stable geometries for CH_3, CH_4, HCOO, HCO, H_2CO and H_3CO on Ni(111). Corresponding chemisorption energy (kJ/mol) and adsorption site is indicated above each picture frame.

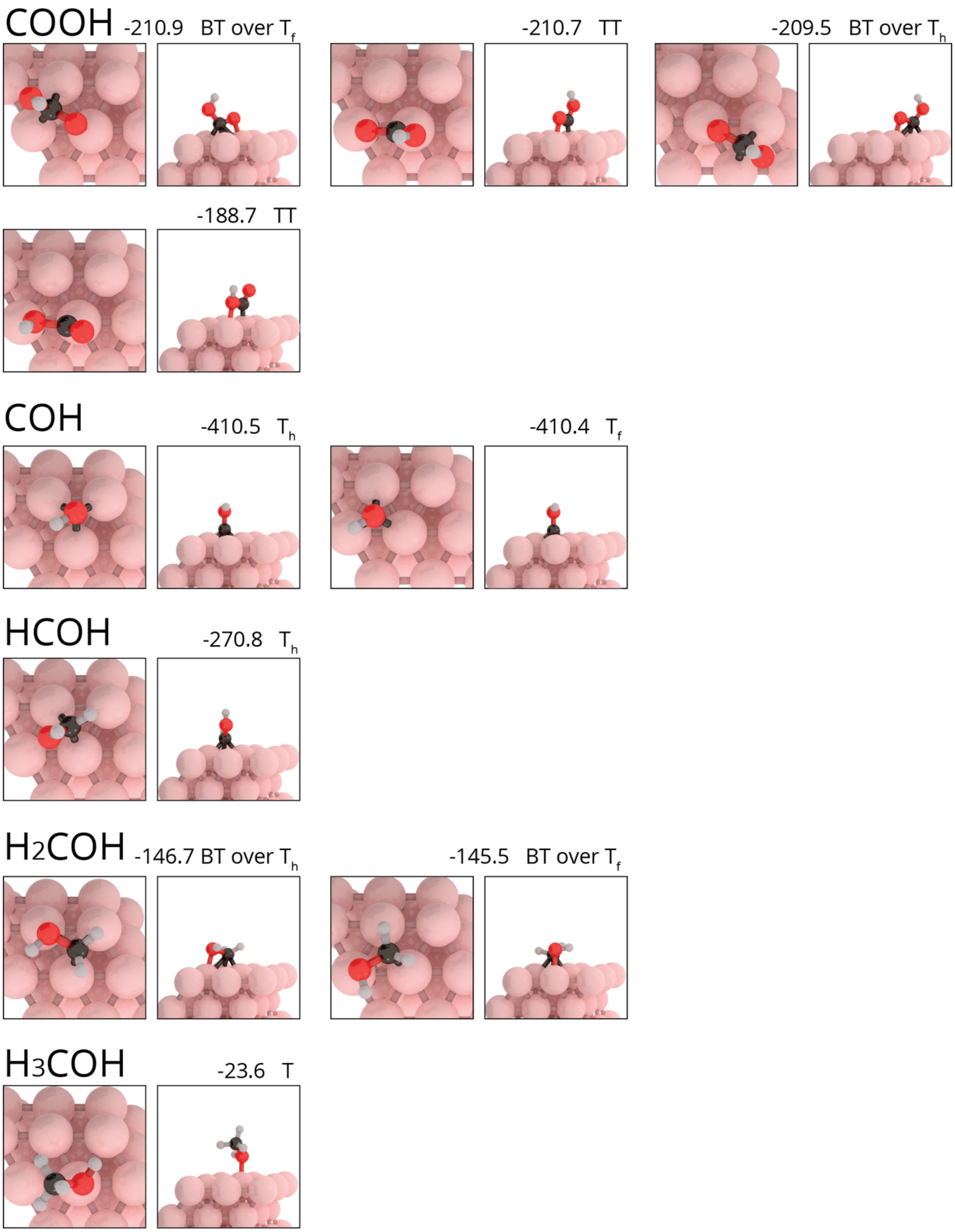

Figure S 3. Top and side view of stable geometries for COOH, COH, HCOH, H_2COH and H_3COH on Ni(111). Corresponding chemisorption energy (kJ/mol) and adsorption site is indicated above each picture frame.

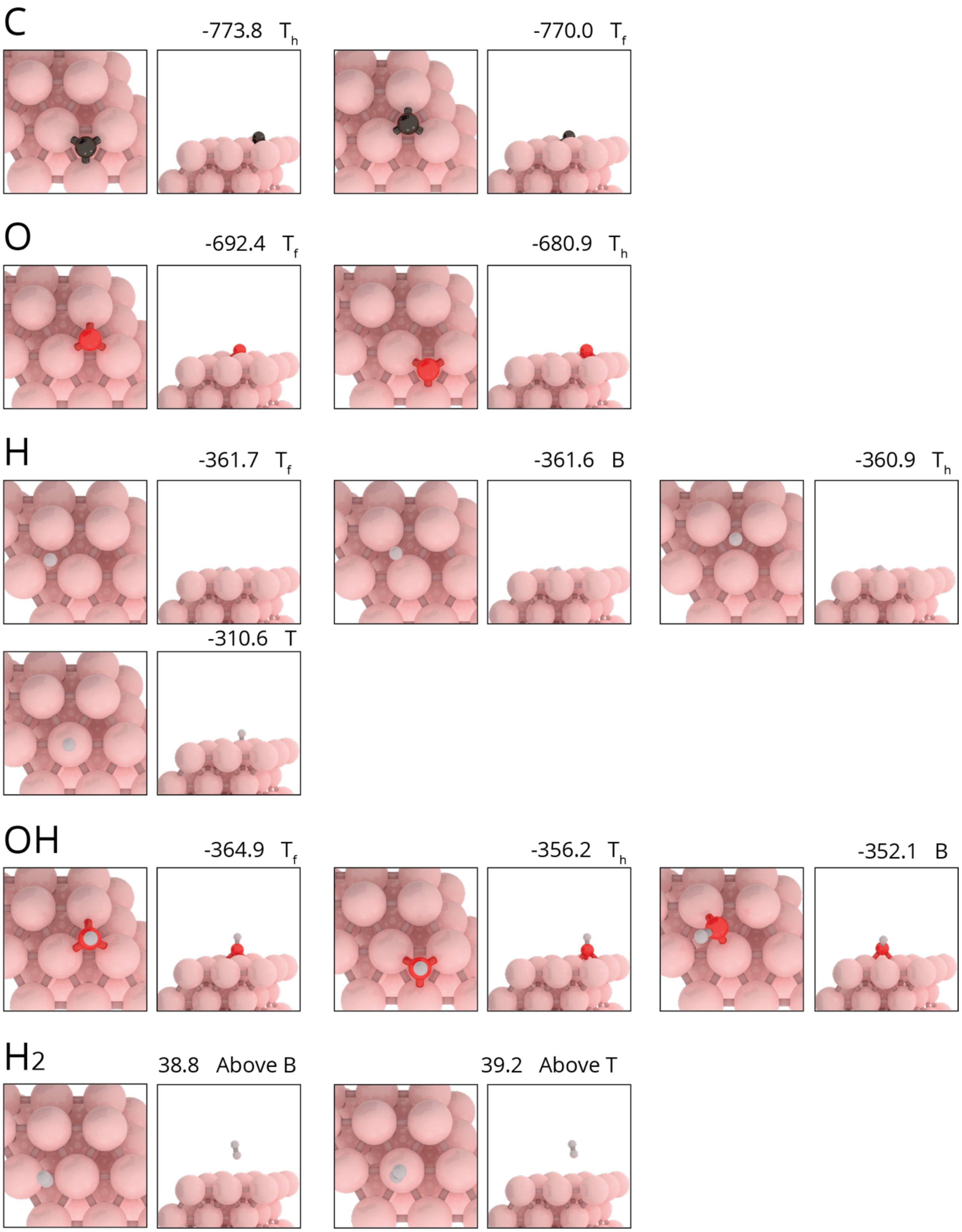

Figure S 4. Top and side view of stable geometries for C, O, H, OH and H_2 on Ni(111). Corresponding chemisorption energy (kJ/mol) and adsorption site is indicated above each picture frame.

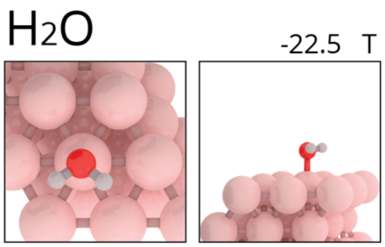

Figure S 5. Top and side view of stable geometry for H_2O Ni(111). Corresponding chemisorption energy (kJ/mol) and adsorption site is indicated above each picture frame.

Ni(100)

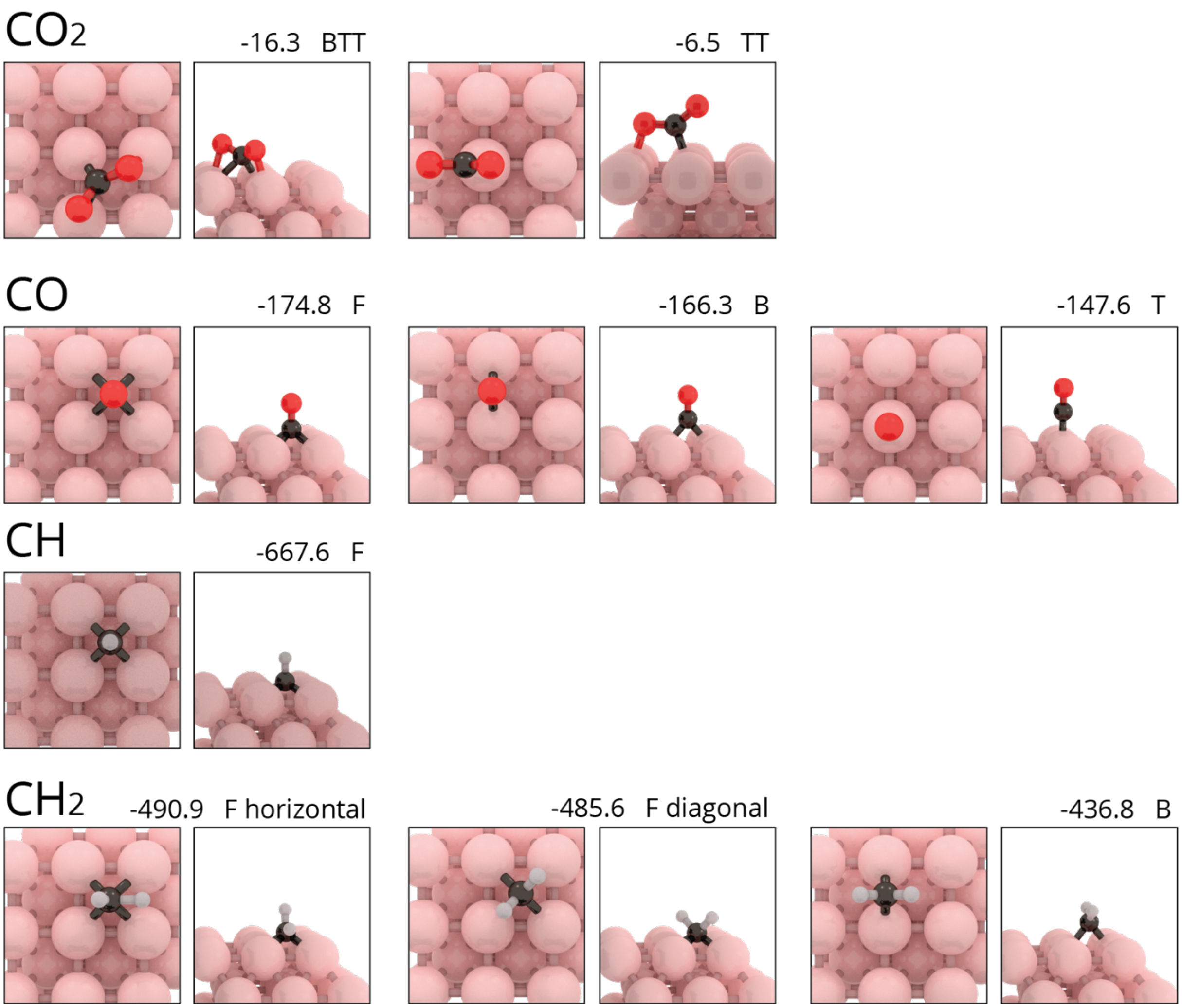

Figure S 6. Top and side view of stable geometries for CO_2, CO, CH and CH_2 on Ni(100). Corresponding chemisorption energy (kJ/mol) and adsorption site is indicated above each picture frame.

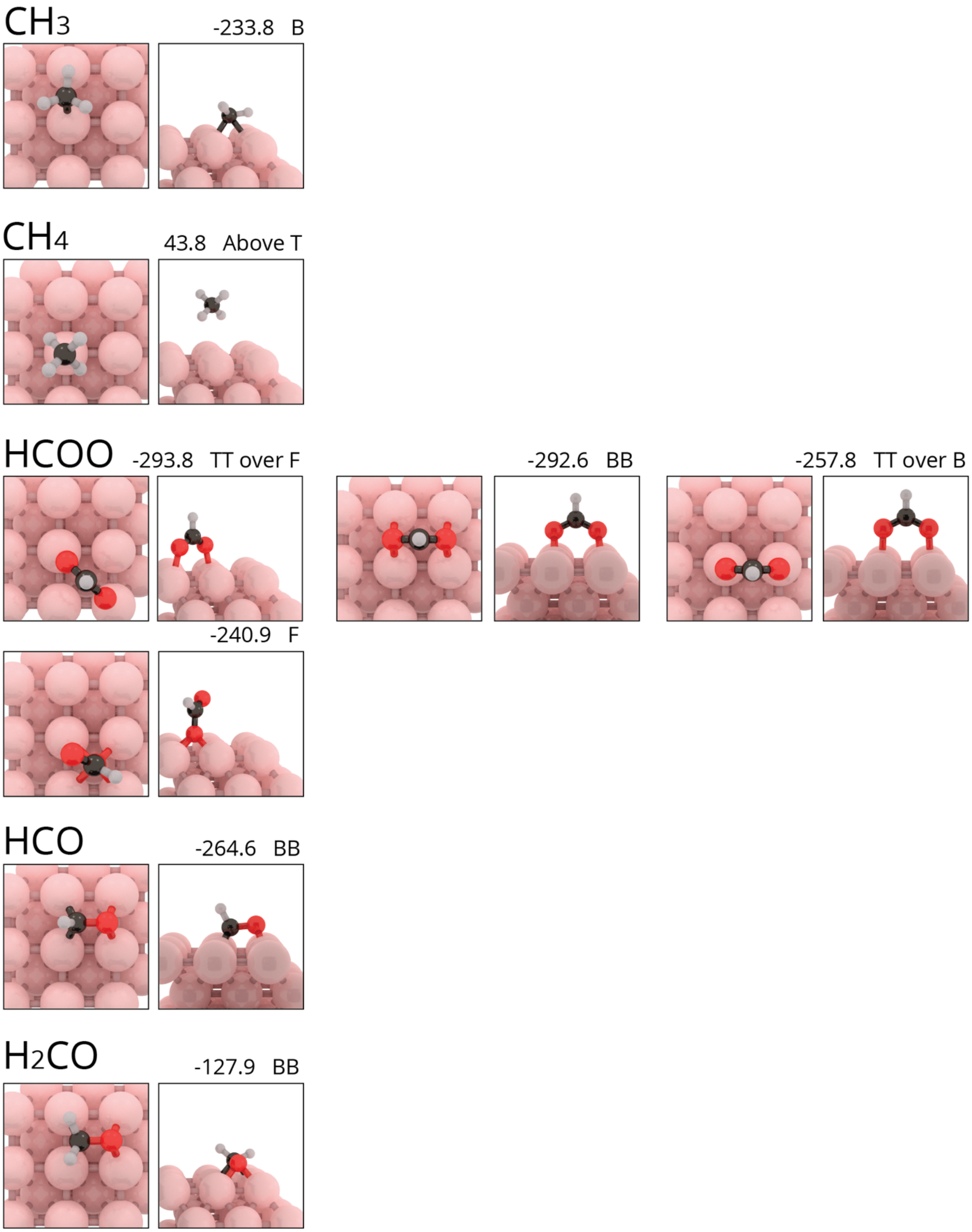

Figure S 7. Top and side view of stable geometries for CH_3, CH_4, HCOO, HCO and H_2CO on Ni(100). Corresponding chemisorption energy (kJ/mol) and adsorption site is indicated above each picture frame.

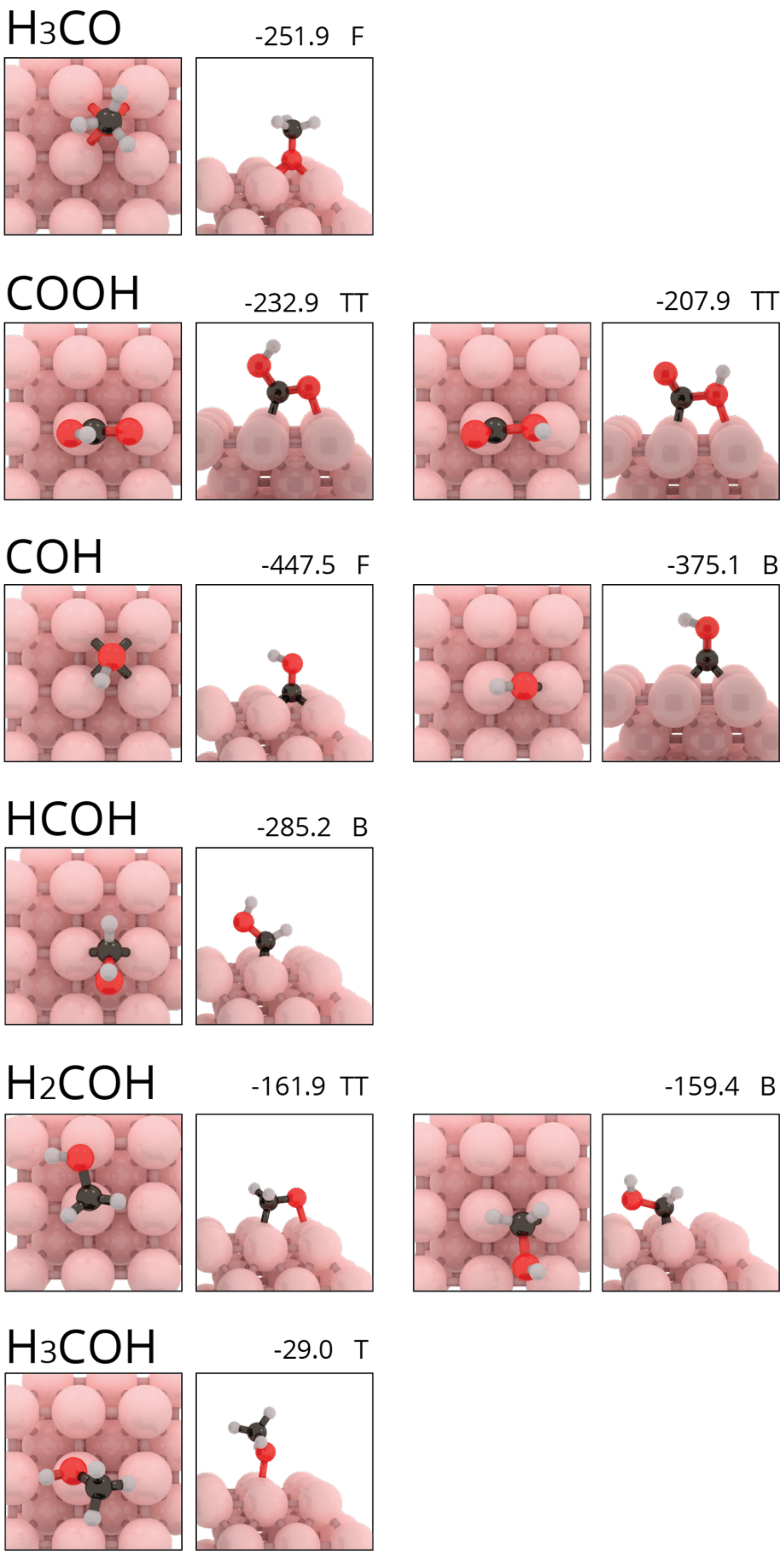

Figure S 8. Top and side view of stable geometries for H_3CO, COOH, COH, HCOH, H_2COH, and H_3COH on Ni(100). Corresponding chemisorption energy (kJ/mol) and adsorption site is indicated above each picture frame.

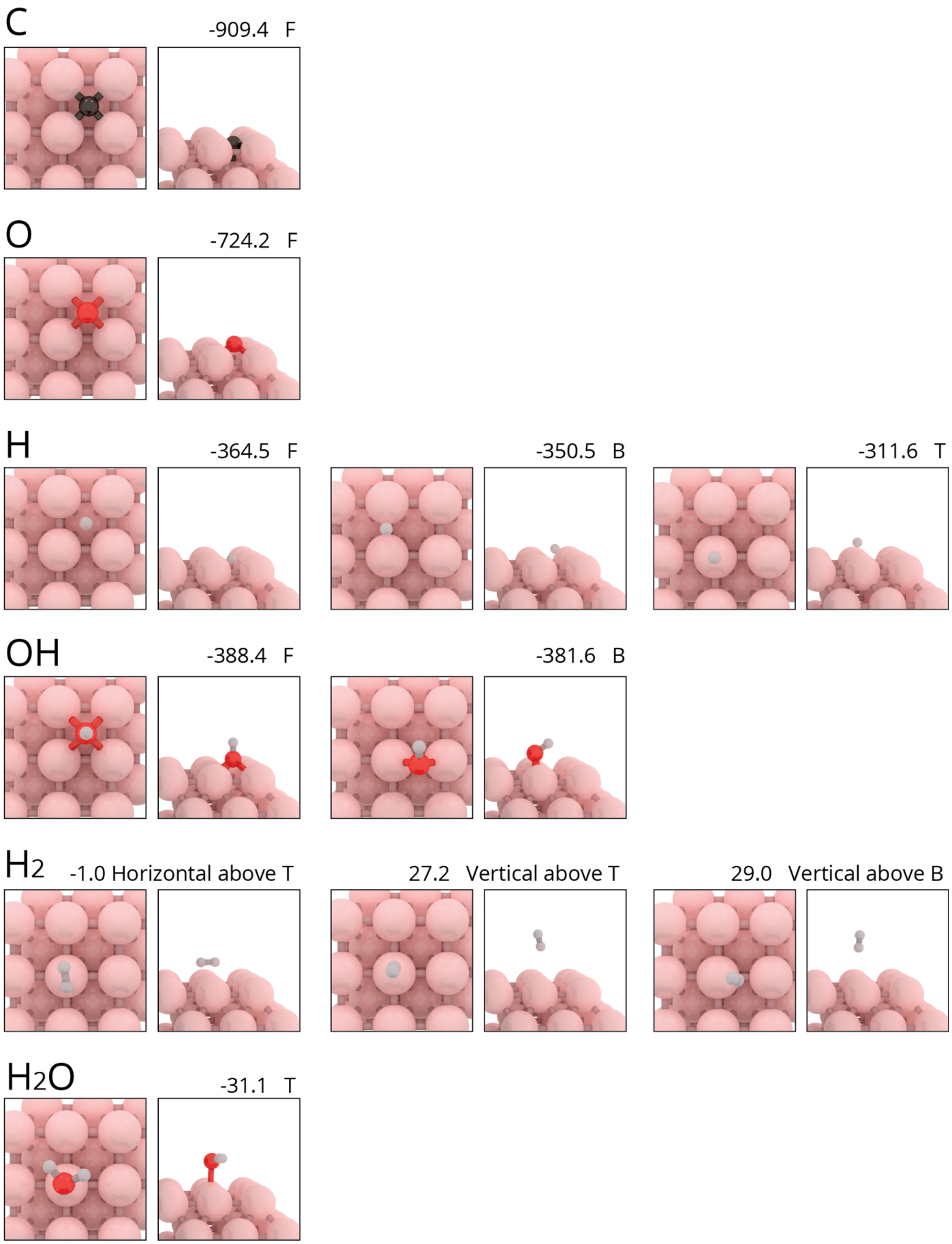

Figure S 9. Top and side view of stable geometries for C, O, H, OH, H_2 and H_2O on Ni(100). Corresponding chemisorption energy (kJ/mol) and adsorption site is indicated above each picture frame.

Ni(110)

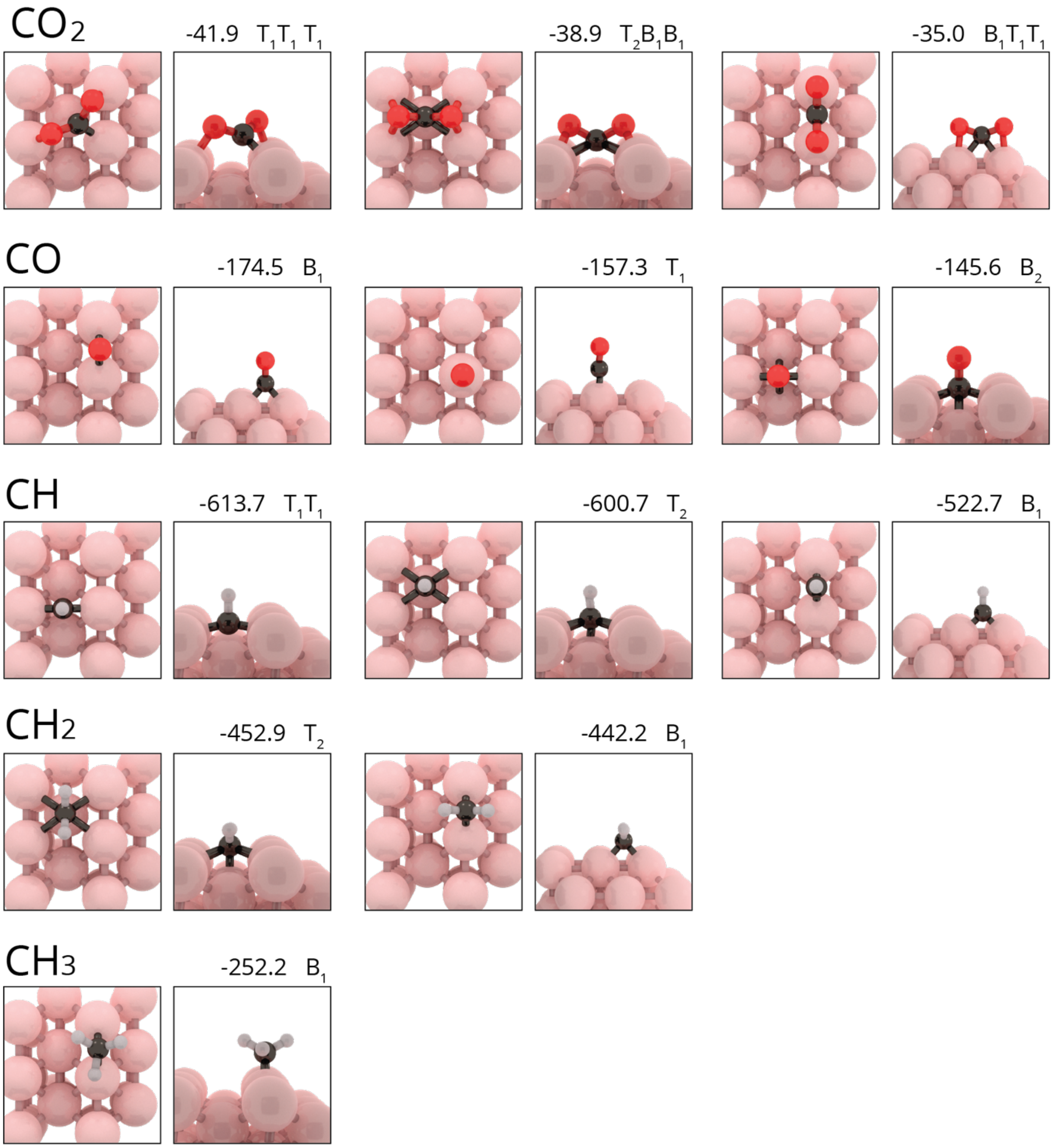

Figure S 10. Top and side view of stable geometries for CO_2, CO, CH, CH_2 and CH_3 on Ni(110). Corresponding chemisorption energy (kJ/mol) and adsorption site is indicated above each picture frame.

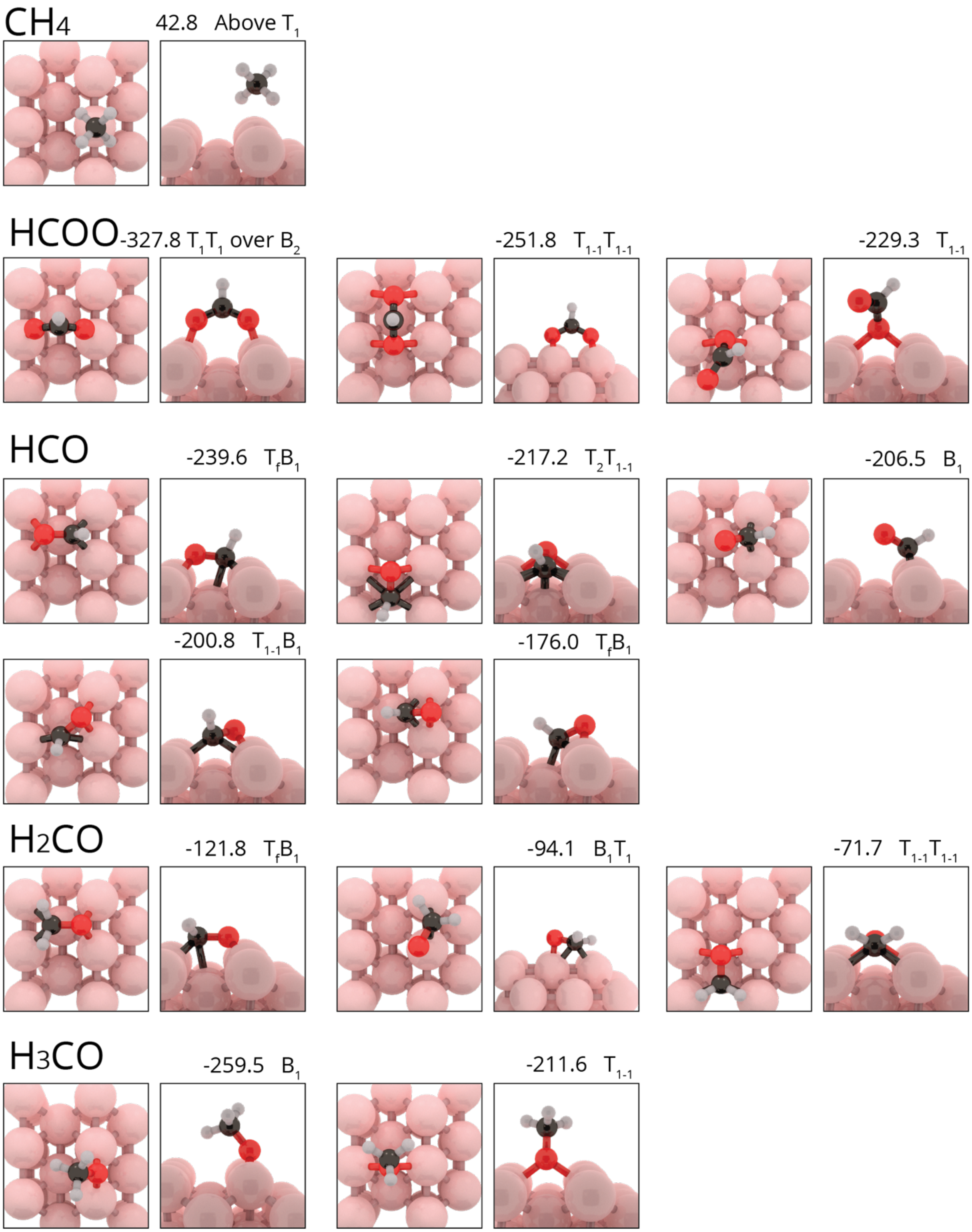

Figure S 11. Top and side view of stable geometries for CH_4, HCOO, HCO, H_2CO and H_3CO on Ni(110). Corresponding chemisorption energy (kJ/mol) and adsorption site is indicated above each picture frame.

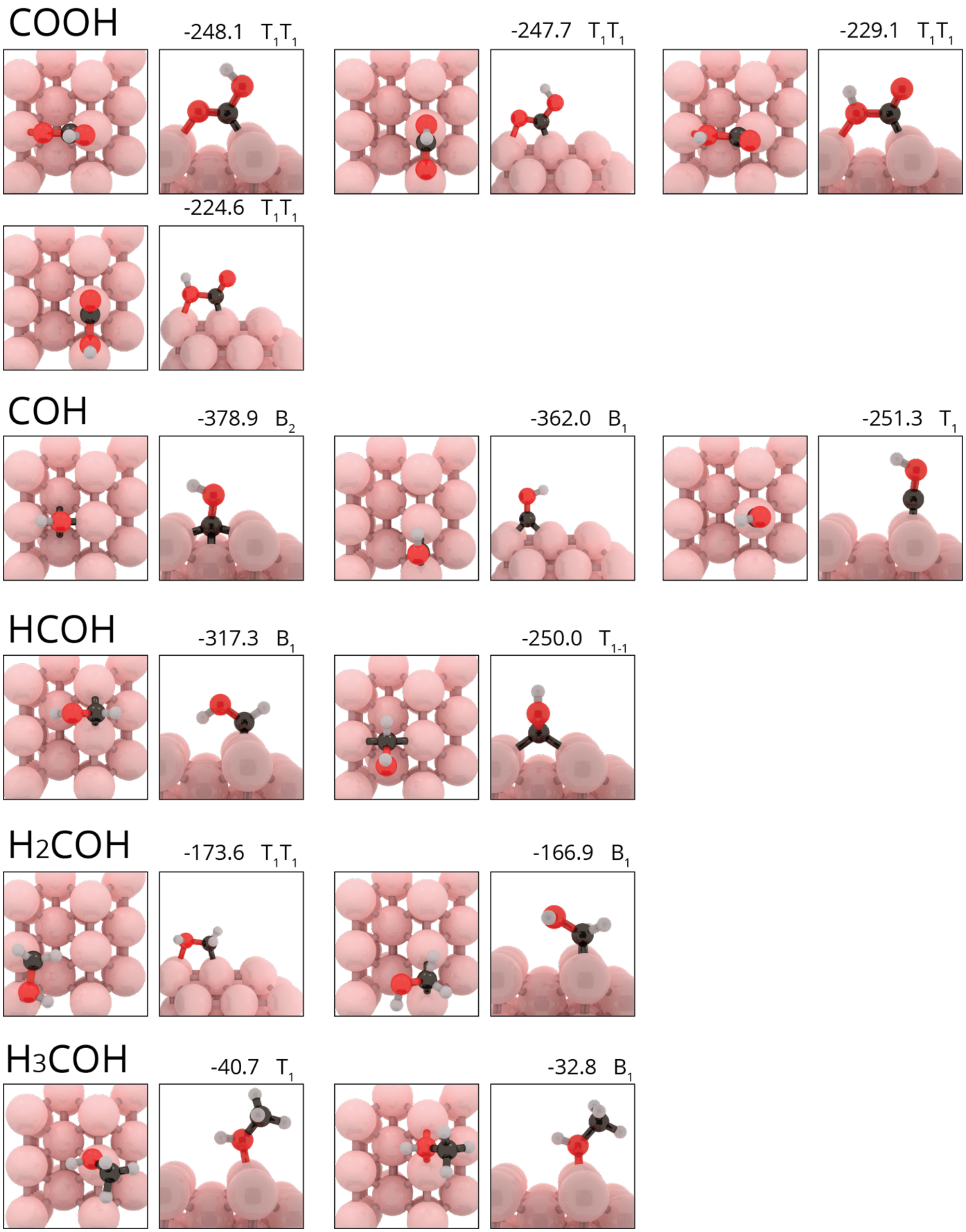

Figure S 12. Top and side view of stable geometries for COOH, COH, HCOH, H_2COH and H_3COH on Ni(110). Corresponding chemisorption energy (kJ/mol) and adsorption site is indicated above each picture frame.

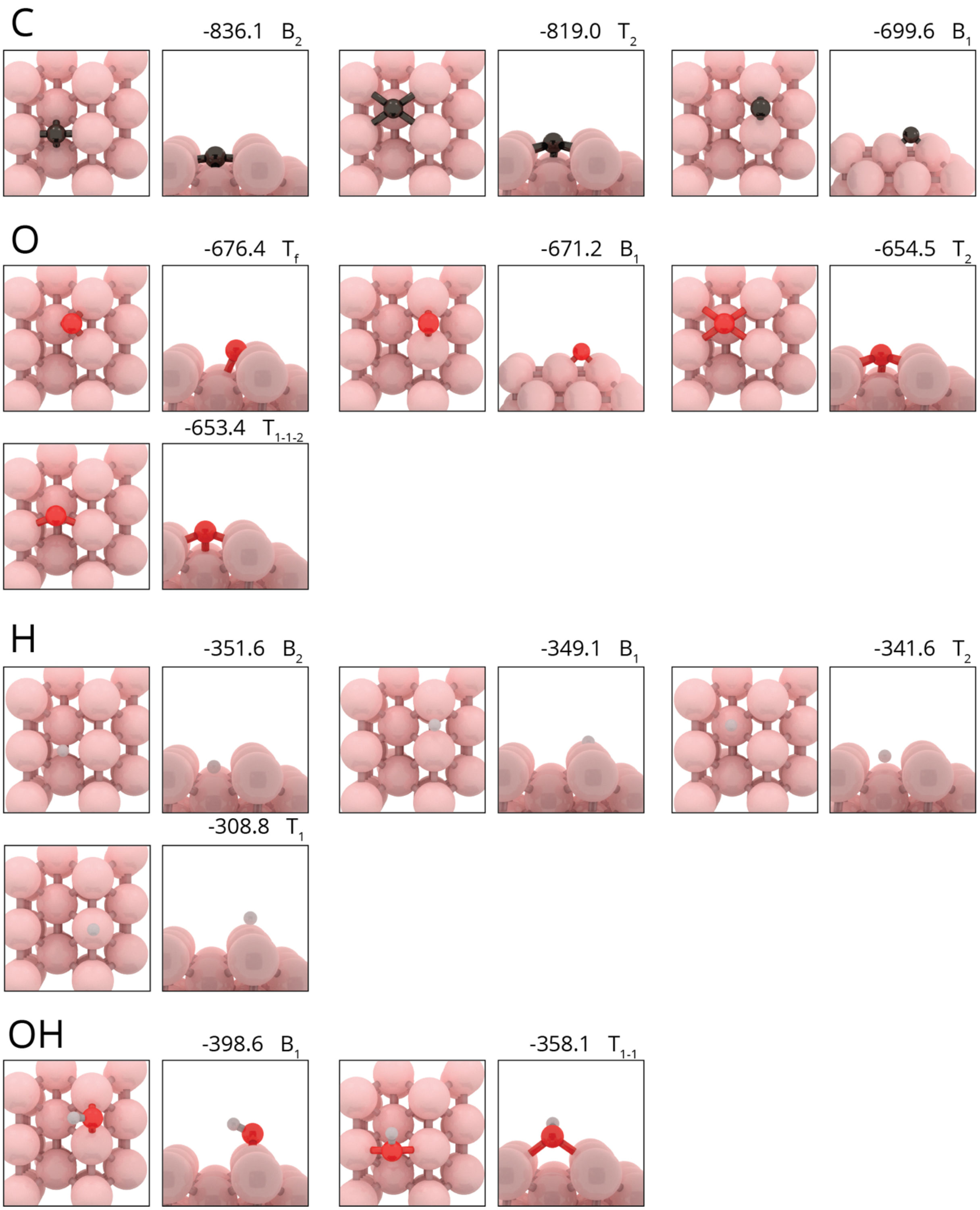

Figure S 13. Top and side view of stable geometries for C, O, H and OH on Ni(110). Corresponding chemisorption energy (kJ/mol) and adsorption site is indicated above each picture frame.

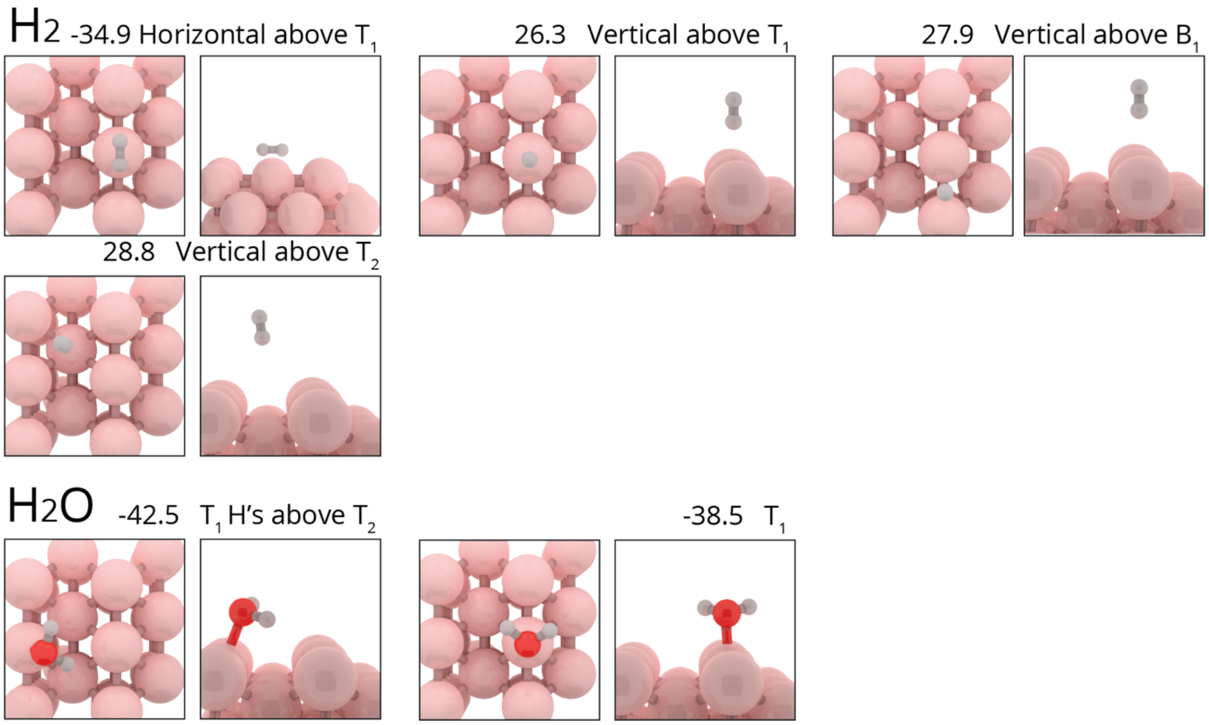

Figure S 14. Top and side view of stable geometries for H_2 and H_2O on Ni(110). Corresponding chemisorption energy (kJ/mol) and adsorption site is indicated above each picture frame.

Ni(211)

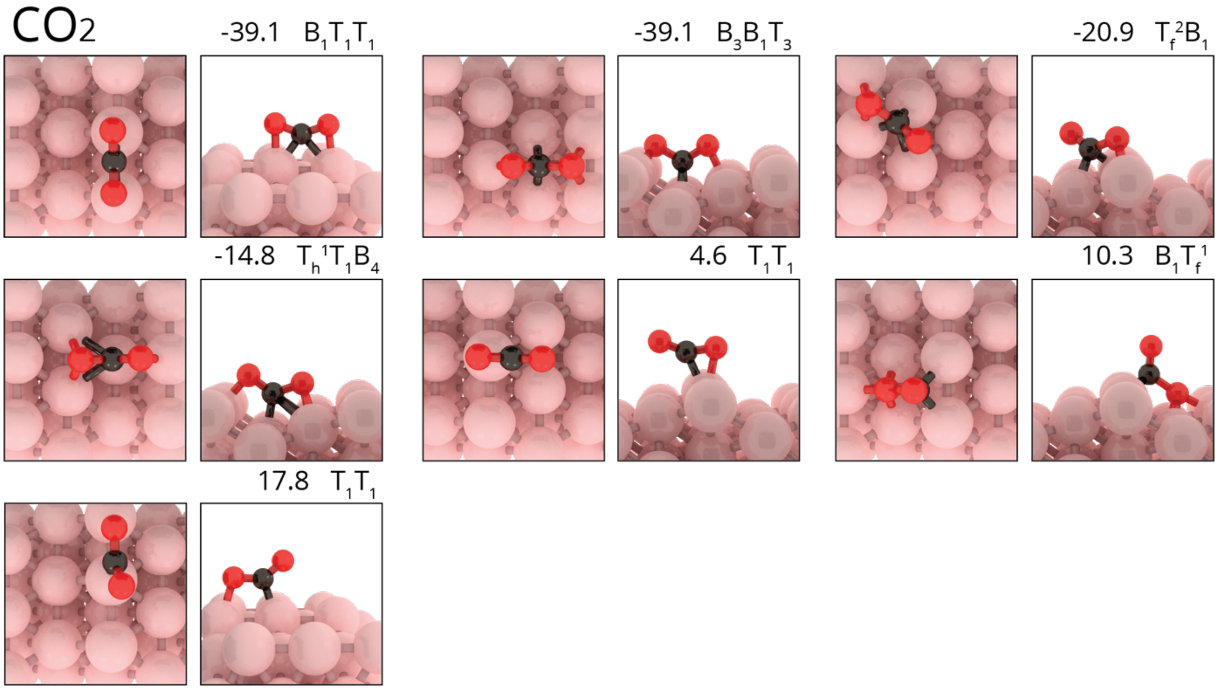

Figure S 15. Top and side view of stable geometries for CO_2 on Ni(211). Corresponding chemisorption energy (kJ/mol) and adsorption site is indicated above each picture frame.

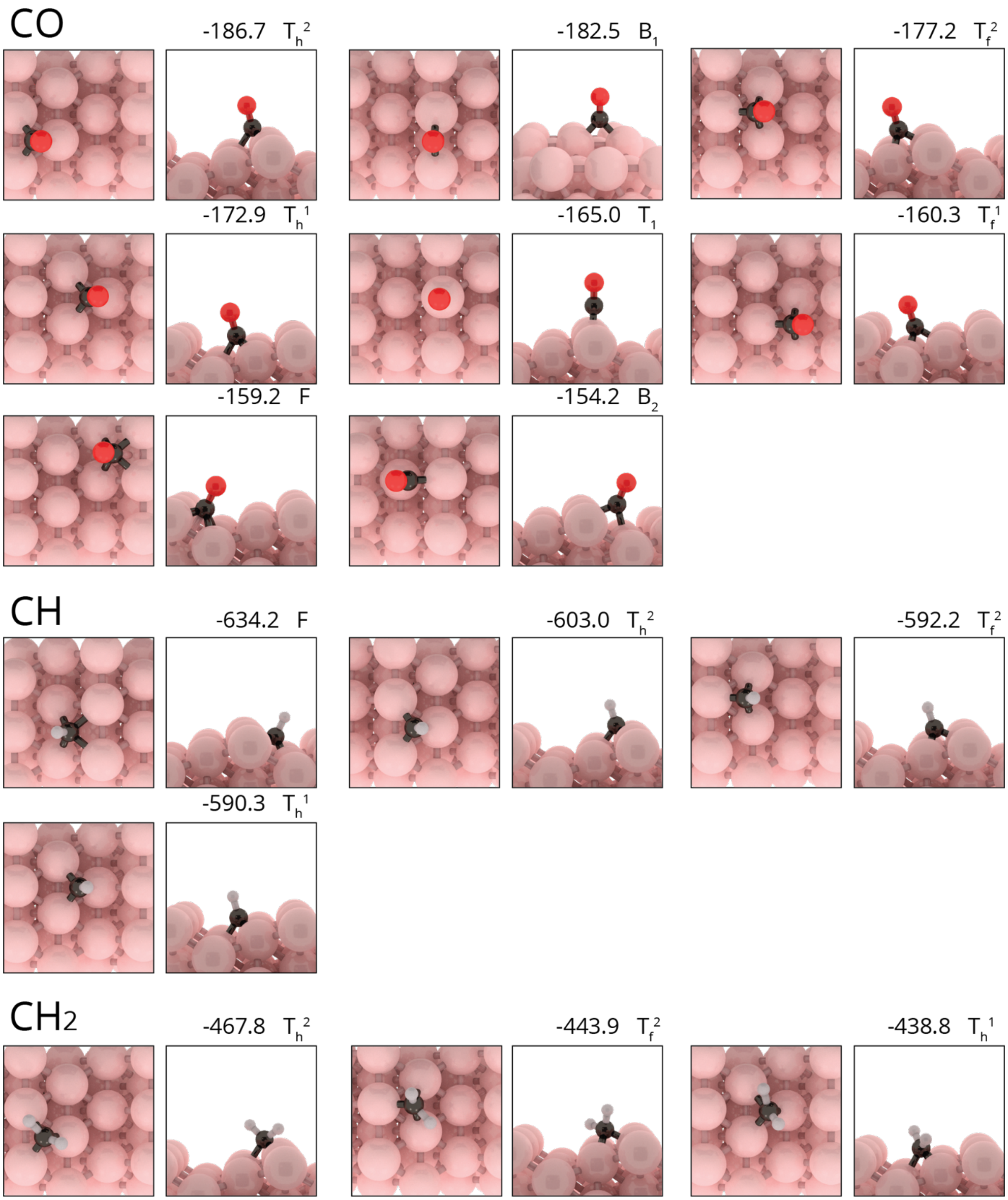

Figure S 16. Top and side view of stable geometries for CO, CH and CH_2 on Ni(211). Corresponding chemisorption energy (kJ/mol) and adsorption site is indicated above each picture frame.

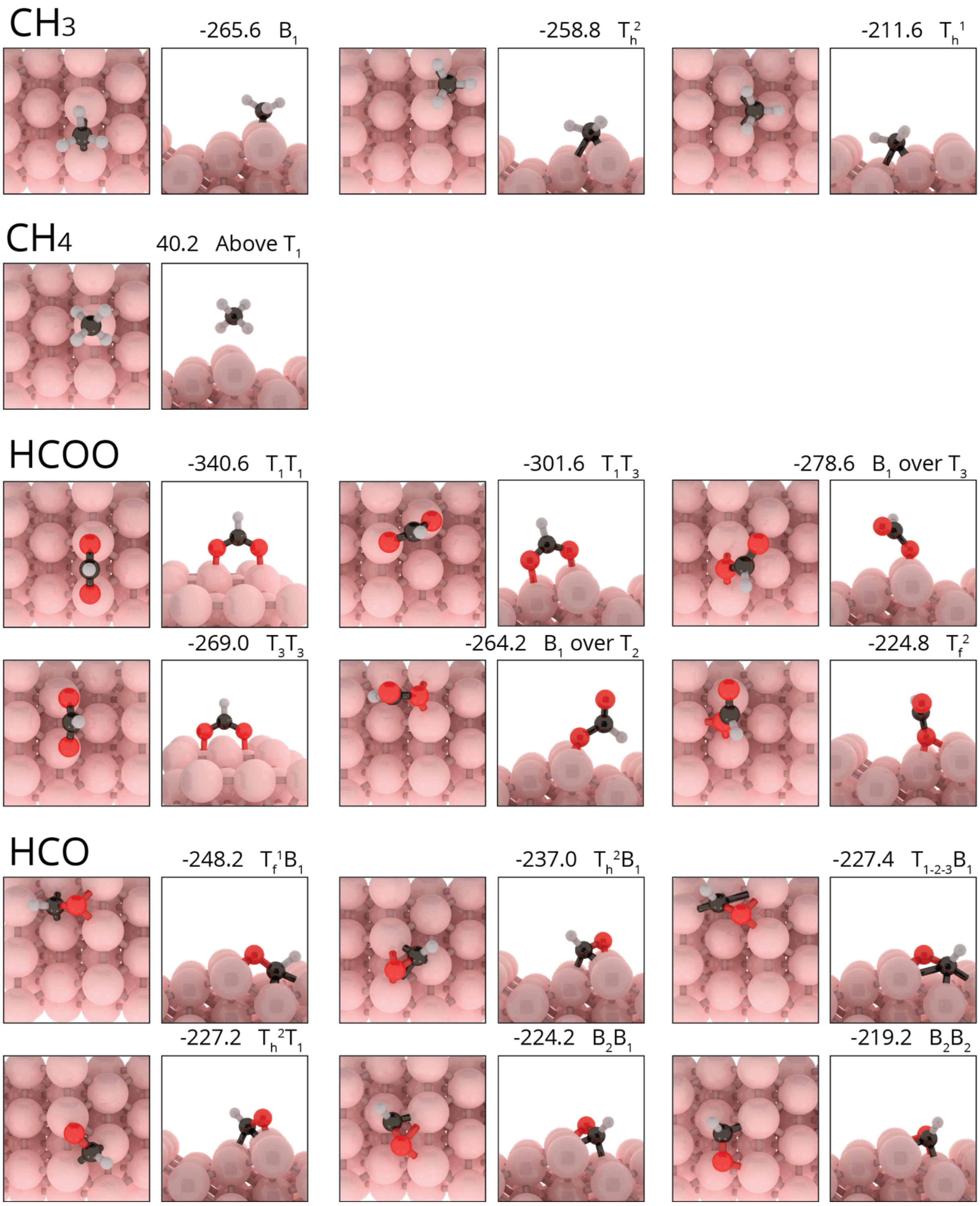

Figure S 17. Top and side view of stable geometries for CH_3, CH_4, HCOO and HCO on Ni(211). Corresponding chemisorption energy (kJ/mol) and adsorption site is indicated above each picture frame.

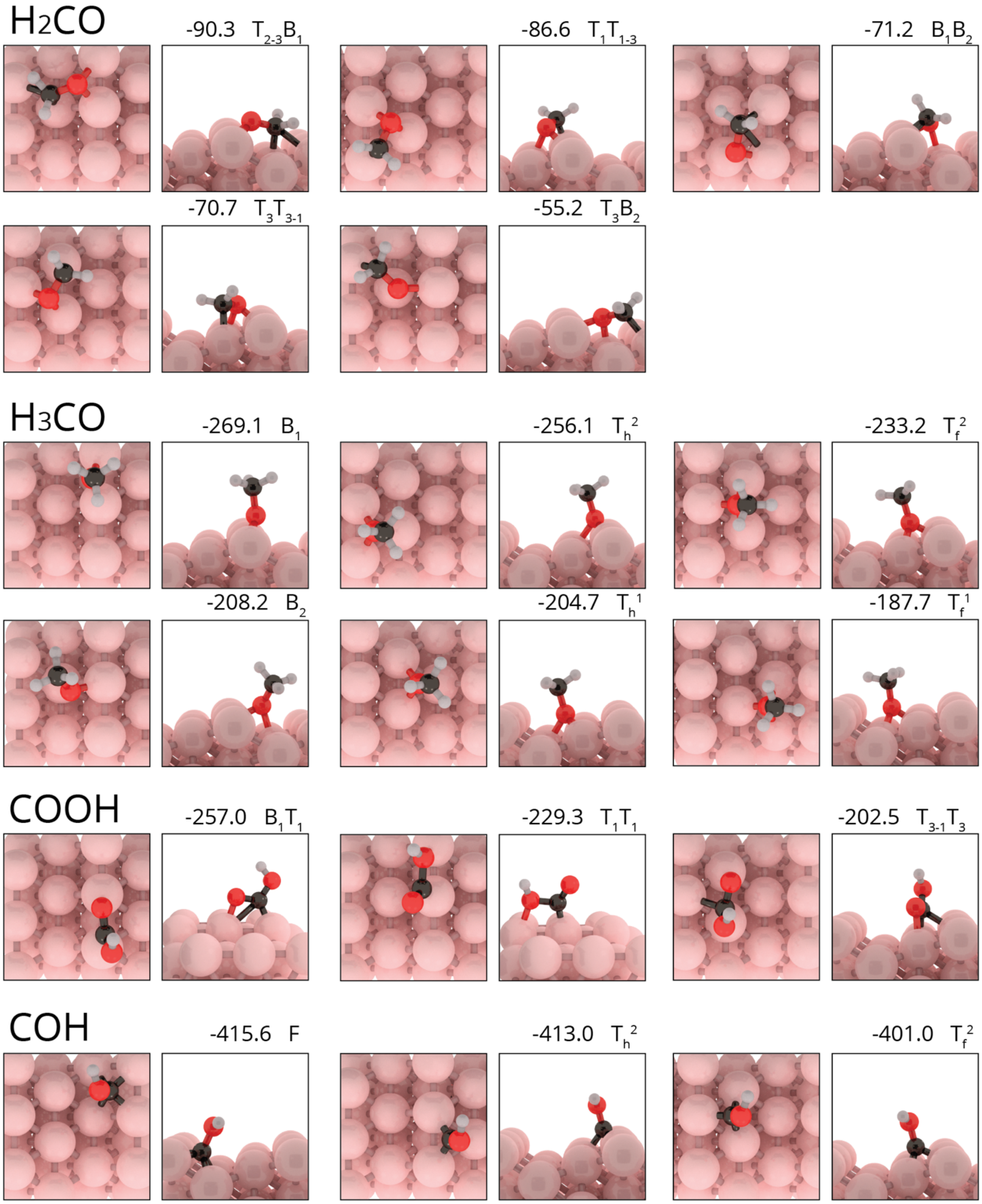

Figure S 18. Top and side view of stable geometries for H_2CO, H_3CO, COOH and COH on Ni(211). Corresponding chemisorption energy (kJ/mol) and adsorption site is indicated above each picture frame.

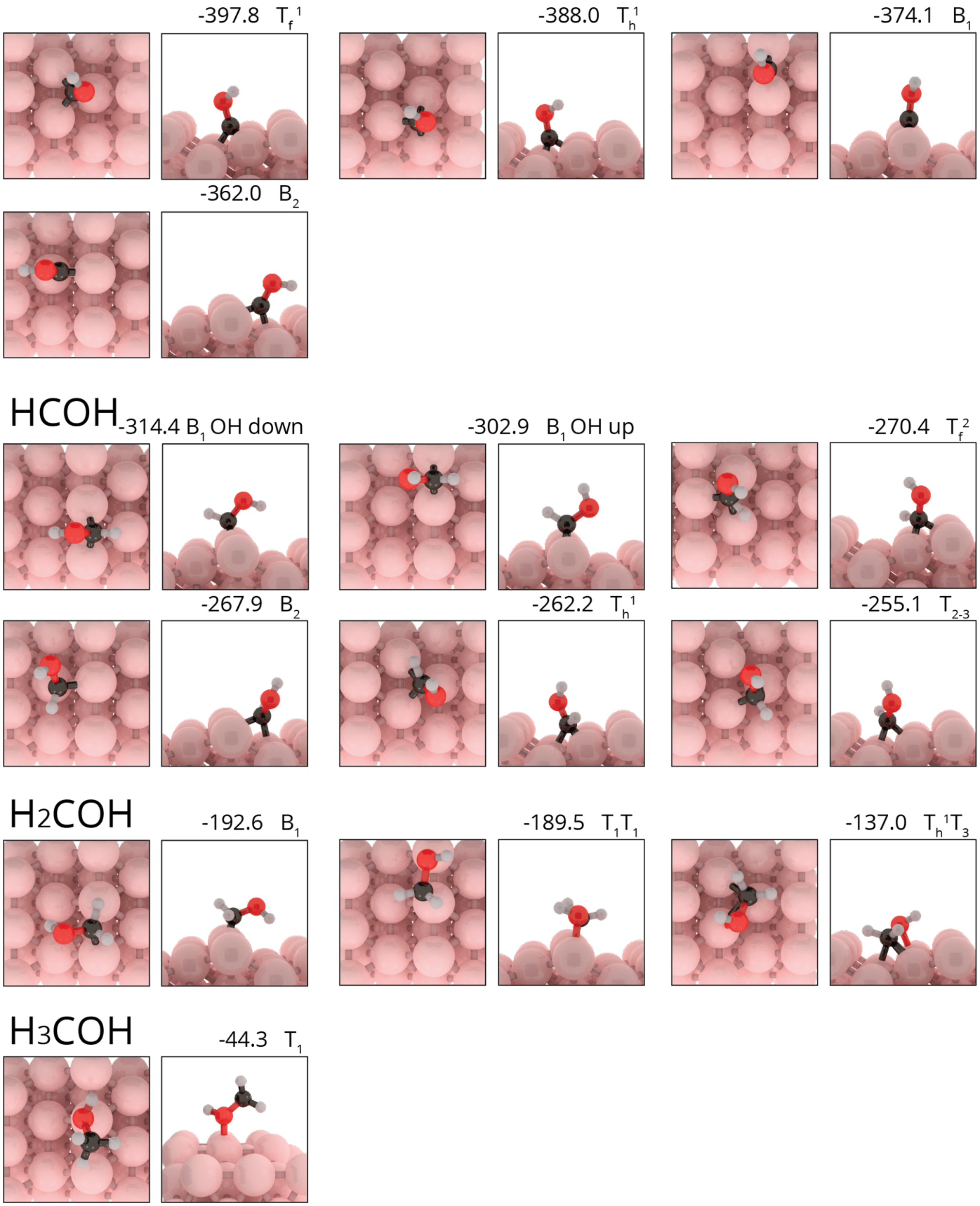

Figure S 19. Top and side view of stable geometries for COH, HCOH, H_2COH and H_3COH on Ni(211). Corresponding chemisorption energy (kJ/mol) and adsorption site is indicated above each picture frame.

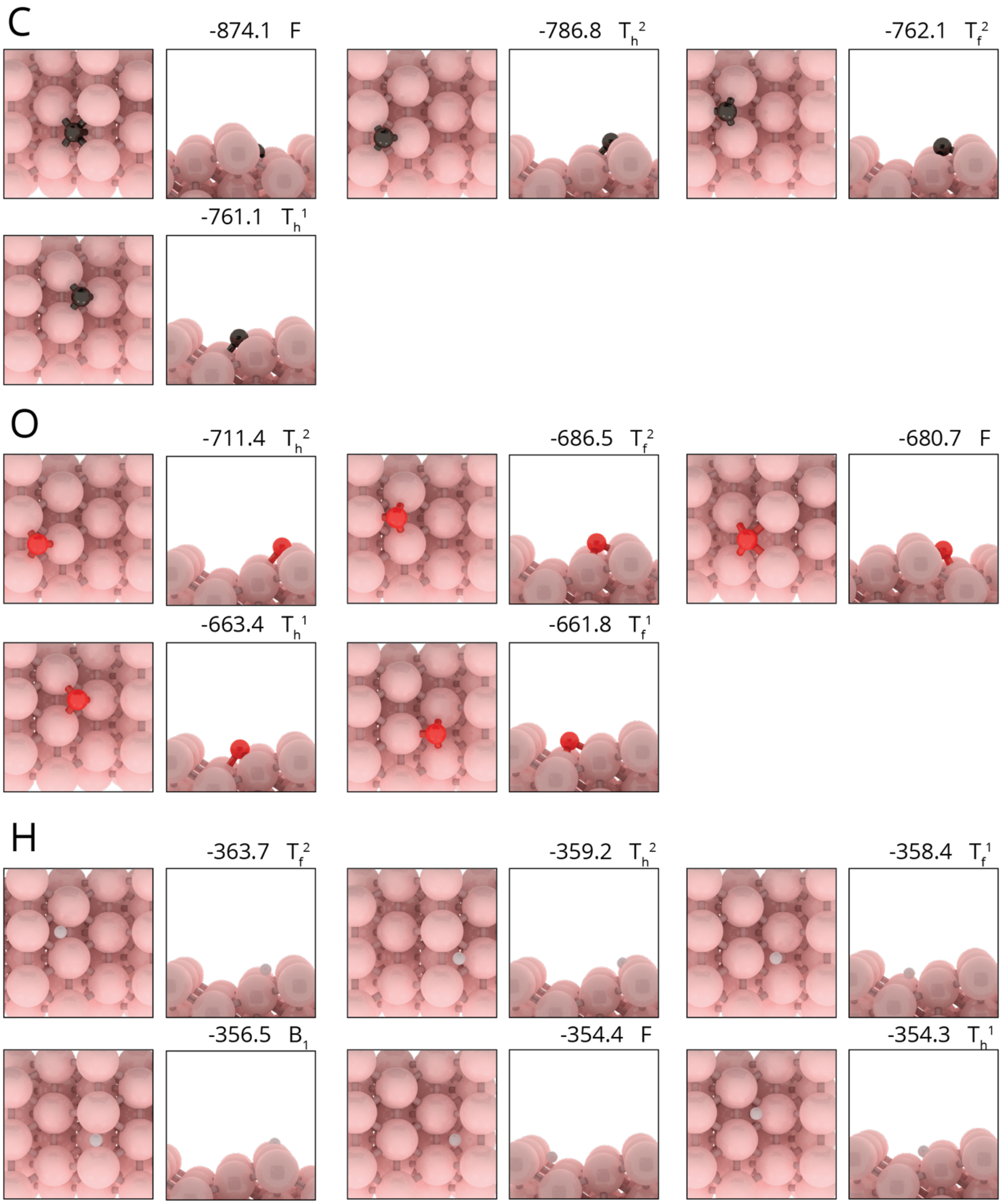

Figure S 20. Top and side view of stable geometries for C, O and H on Ni(211). Corresponding chemisorption energy (kJ/mol) and adsorption site is indicated above each picture frame.

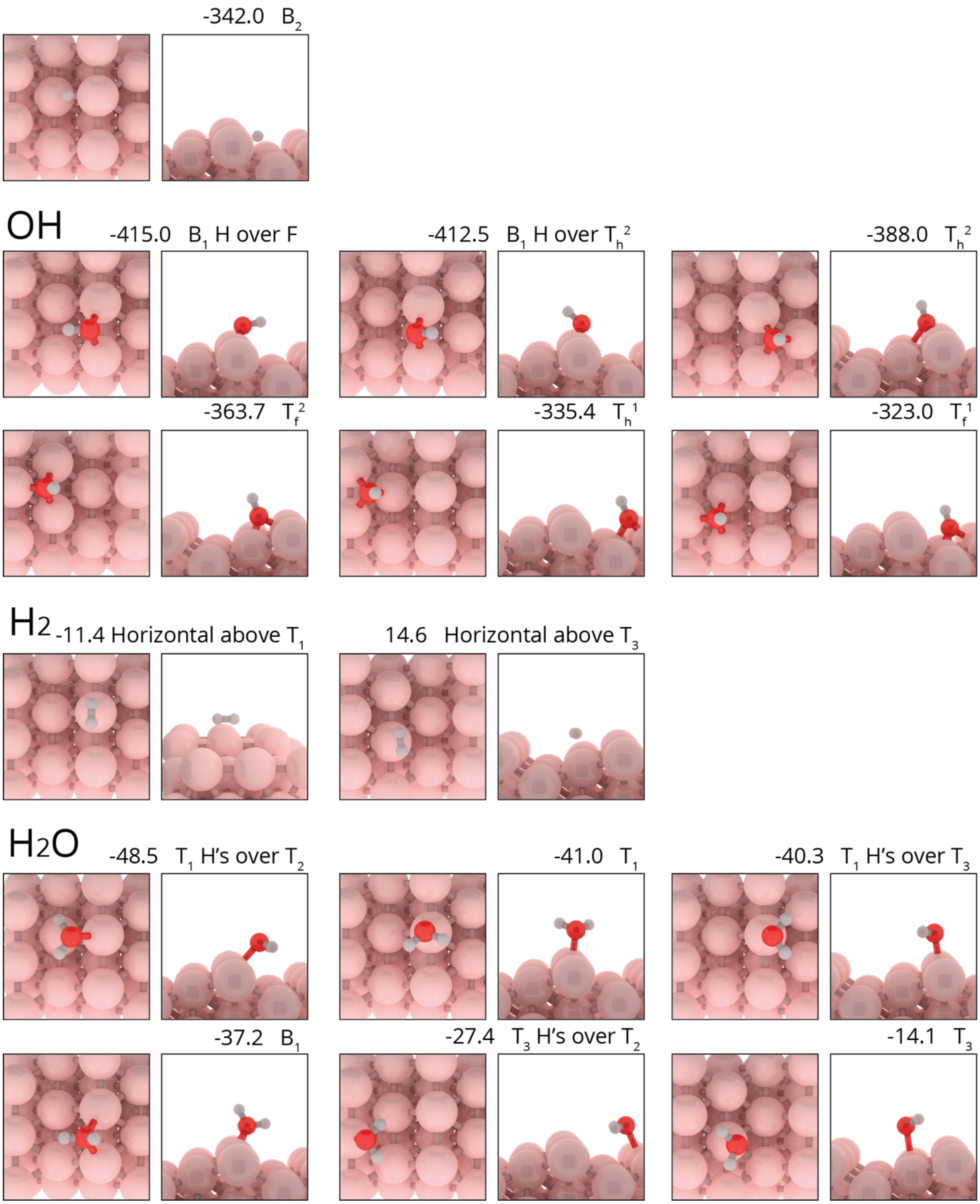

Figure S 21. Top and side view of stable geometries for H, OH, H_2 and H_2O on Ni(211). Corresponding chemisorption energy (kJ/mol) and adsorption site is indicated above each picture frame.

E Table Adsorption Energy of Stable Geometries

In Table S 8 to Table S 11 adsorption energies (kJ/mol) of stable geometries on Ni(111), Ni(100), Ni(110) and Ni(211).

Table S 8. Adsorption energies (kJ/mol) of stable geometries on Ni(111).

Ni(111)					
**Adsorbate**	**Adsorption Site**	$\mathbf{E}_{ads}$	**Adsorbate**	**Adsorption Site**	$\mathbf{E}_{ads}$
CO_2	T_fTT	24.3	C	T_h	-773.8
	TT	26.6		T_f	-770.0
	BTT	35.5	O	T_f	-692.4
CO	T_h	-178.6		T_h	-680.9
	T_f	-178.1	H	T_f	-361.7
	B	-158.8		B	-361.6
	T	-144.3		T_h	-360.9
CH	T_f	-603.3		T	-310.6
	T_h	-600.5	OH	T_f	-364.9
CH_2	T_f	-454.2		T_h	-356.2
	T_h	-449.7		B	-352.1
CH_3	T_f	-238.3	H_2	Above B	38.8
	T_h	-235.8		Above T	39.2
CH_4	Above T	43.6	H_2O	T	-22.5
HCOO	TT	-277.3			
	T_f	-224.3			
	T_h	-218.1			
HCO	BT over T_f	-201.2			
	BT over T_h	-200.8			
	T	-165.9			
H_2CO	T_fT	-57.7			
	T_hT	-57.3			
H_3CO	T_h	-218.0			
	T_f	-206.4			
COOH	BT over T_f	-210.9			
	TT	-210.7			
	BT over T_h	-209.5			
	TT	-188.7			
COH	T_h	-410.5			
	T_f	-410.4			
HCOH	T_h	-270.8			
H_2COH	BT over T_h	-146.7			
	BT over T_f	-145.5			
H_3COH	T	-23.6			

Table S 9. Adsorption energies (kJ/mol) of stable geometries on Ni(100).

Ni(100)		
**Adsorbate**	**Adsorption Site**	$\mathbf{E}_{ads}$
CO_2	BTT	-16.3
	TT	-6.5
CO	F	-174.8
	B	-166.3
	T	-147.6
CH	F	-667.6
CH_2	F horizontal	-490.9
	F diagonal	-485.6
	B	-436.8
CH_3	B	-233.8
CH_4	Above T	43.8
HCOO	TT over F	-293.8
	BB	-292.6
	TT over B	-257.8
	F	-240.9
HCO	BB	-264.6
H_2CO	BB	-127.9
H_3CO	F	-251.9
COOH	TT	-232.9
	TT	-207.9
COH	F	-447.5
	B	-375.1
HCOH	B	-285.2
H_2COH	TT	-161.9
	B	-159.4
H_3COH	T	-29.0
C	F	-909.4
O	F	-724.2
H	F	-364.5
	B	-350.5
	T	-311.6
OH	F	-388.4
	B	-381.6
H_2	Horizontal above T	-1.0
	Vertical above T	27.2
	Vertical above B	29.0
H_2O	T	-31.1

Table S 10. Adsorption energies (kJ/mol) of stable geometries on Ni(110).

Ni(110)					
**Adsorbate**	**Adsorption Site**	$\mathbf{E_{ads}}$	**Adsorbate**	**Adsorption Site**	$\mathbf{E_{ads}}$
CO_2	$T_1T_1T_1$	-41.9	C	B_2	-836.1
	$T_2B_1B_1$	-38.9		T_2	-819.0
	$B_1T_1T_1$	-35.0		B_1	-699.6
CO	B_1	-174.5	O	T_f	-676.4
	T_1	-157.3		B_1	-671.2
	B_2	-145.6		T_2	-654.5
CH	T_1T_1	-613.7		T_{1-1-2}	-653.4
	T_2	-600.7	H	B_2	-351.6
	B_1	-522.7		B_1	-349.1
CH_2	T_2	-452.9		T_2	-341.6
	B_1	-442.2		T_1	-308.8
CH_3	B_1	-252.2	OH	B_1	-398.6
CH_4	Above T_1	42.8		T_{1-1}	-358.1
HCOO	T_1T_1 over B_2	-327.8	H_2	Horizontal above T_1	-34.9
	$T_{1-1}T_{1-1}$	-251.8		Vertical above T_1	26.3
	T_{1-1}	-229.3		Vertical above B_1	27.9
HCO	T_fB_1	-239.6		Vertical above T_2	28.8
	T_2T_{1-1}	-217.2	H_2O	T_1 H's above T_2	-38.5
	B_1	-206.5		T_1	-38.5
	$T_{1-1}B_1$	-200.8			
	T_fB_1	-176.0			
H_2CO	T_fB_1	-121.8			
	B_1T_1	-94.1			
	$T_{1-1}T_{1-1}$	-71.7			
H_3CO	B_1	-259.5			
	T_{1-1}	-211.6			
COOH	T_{1-1}	-248.1			
	T_{1-1}	-247.7			
	T_{1-1}	-229.1			
	T_{1-1}	-224.6			
COH	B_2	-378.9			
	B_1	-362.0			
	T_1	-251.3			
HCOH	B_1	-317.3			
	T_{1-1}	-250.0			
H_2COH	T_1T_1	-173.6			
	B_1	-166.9			
H_3COH	T_1	-40.7			
	B_1	-32.8			

Table S 11. Adsorption energies (kJ/mol) of stable geometries on Ni(211). Table continues on next page.

Ni(211) part 1					
**Adsorbate**	**Adsorption Site**	$\mathbf{E_{ads}}$	**Adsorbate**	**Adsorption Site**	$\mathbf{E_{ads}}$
CO_2	$B_1T_1T_1$	-39.1		B_1B_2	-71.2
	$B_3B_1T_3$	-39.1		T_3T_{3-1}	-70.7
	$T_f^2B_1$	-20.9		T_3B_2	-55.2
	$T_h^1T_1B_4$	-14.8	H_3CO	B_1	-269.1
	T_1T_1	4.6		T_h^2	-256.1
	$B_1T_f^1$	10.3		T_f^2	-233.2
	T_1T_1	17.8		B_2	-208.2
CO	T_h^2	-186.7		T_h^1	-204.7
	B_1	-182.5		T_f^1	-187.7
	T_f^2	-177.2	COOH	B_1T_1	-257.0
	T_h^1	-172.9		T_1T_1	-229.3
	T_1	-165.0		$T_{3-1}T_3$	-202.5
	T_f^1	-160.3	COH	F	-415.6
	F	-159.2		T_h^2	-413.0
	B_2	-154.2		T_f^2	-401.0
CH	F	-634.2		T_h^1	-397.8
	T_h^2	-603.0		T_f^1	-388.0
	T_f^2	-592.2		B_1	-374.1
	T_h^1	-590.3		B_2	-362.0
CH_2	T_h^2	-467.8	HCOH	B_1 OH down	-314.4
	T_f^2	-443.9		B_1 OH up	-302.9
	T_h^1	-438.8		T_f^2	-270.4
CH_3	B_1	-265.6		B_2	-267.9
	T_h^2	-258.8		T_h^1	-262.2
	T_h^1	-211.6		T_{2-3}	-255.1
CH_4	Above T_1	40.2	H_2COH	B_1	-192.6
HCOO	T_1T_1	-340.6		T_1T_1	-189.5
	T_1T_2	-301.6		$T_h^1T_3$	-137.0
	B_1 over upper edge	-278.6	C	F	-874.1
	T_3T_3	-269.0		T_h^2	-786.8
	B_1 over lower edge	-264.2		T_f^1	-762.1
	T_F^2	-224.8		T_h^1	-761.1
HCO	$T_f^1B_1$	-248.2	O	T_h^2	-711.4
	$T_h^2B_1$	-237.0		T_f^2	-686.5
	$T_{1-2-3}B_1$	-227.4		F	-680.7
	$T_h^2T_1$	-227.2		T_h^1	-663.4
	B_2B_1	-224.2		T_f^1	-661.8
	B_2B_2	-219.2	H	T_f^2	-363.7
H_2CO	$T_{2-3}B_1$	-90.3		T_h^2	-359.2
	T_1T_{1-3}	-86.6		T_f^1	-358.4

Ni(211) part 2					
**Adsorbate**	**Adsorption Site**	$\mathbf{E}_{ads}$	**Adsorbate**	**Adsorption Site**	$\mathbf{E}_{ads}$
H	B_1	-356.5	H_2	horizontal above T_1	-38.9
	F	-354.4		horizontal above T_3	-12.9
	T_h^1	-354.3	H_2O	T_1 H's over lower edge	-48.5
	B_2	-342.0		T_1 H's over step edge	-41.0
OH	B_1 H over lower edge	-415.0	T_1 H's over upper edge	-40.3	
	B_1 H over upper egde	-412.5	B_1	-37.2	
	T_h^2	-388.0	T_3 H's over lower edge	-27.4	
	T_f^2	-363.7	T_3 H's over upper edge	-14.1	
	T_h^1	-335.4			
	T_f^1	-323.0			

F Geometries of Elementary Reaction Steps

The geometries of the initial-, transition and final states of relevant elementary reaction steps in CO_2 methanation over four nickel facets shown in Figure S 22 to Figure S 30.

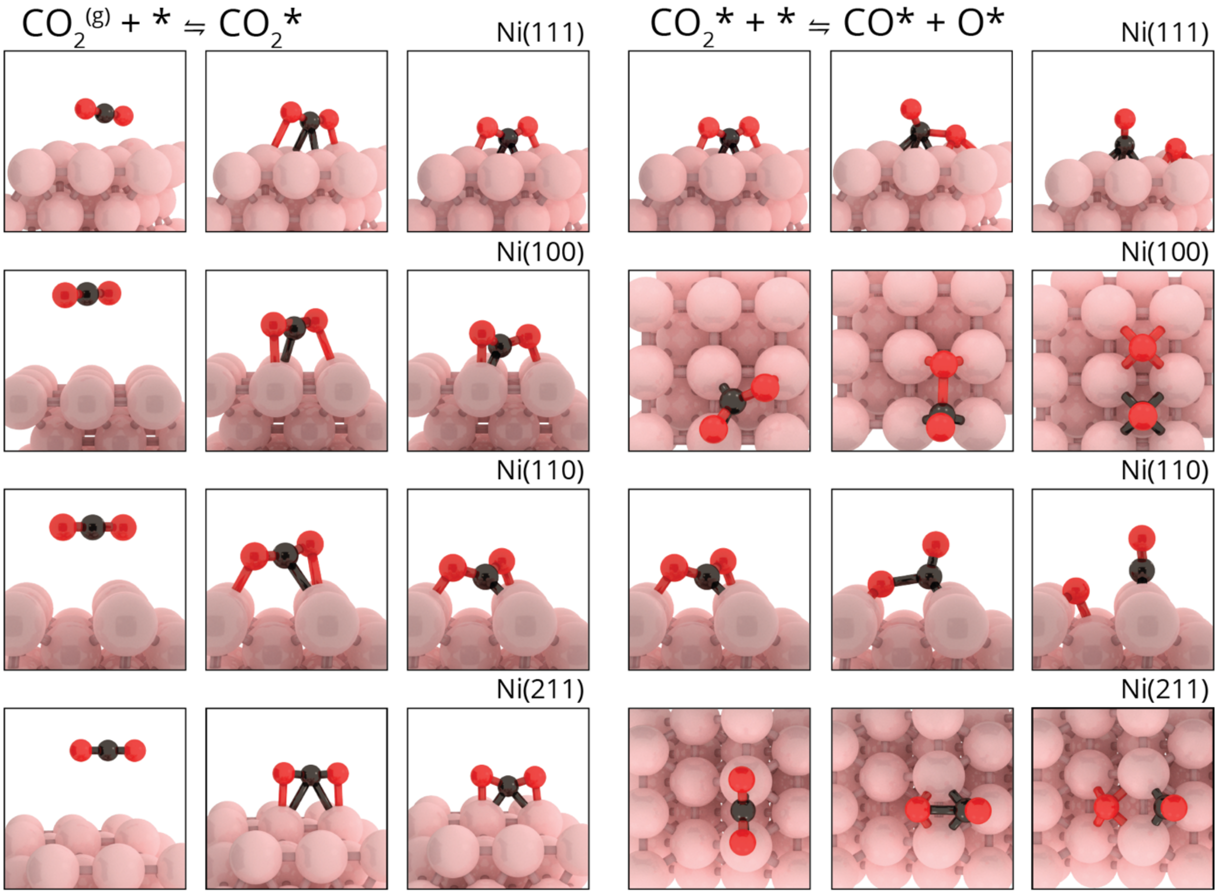

Figure S 22. Geometries of the initial, transition and final states for the elementary reactions $CO_2^{(g)} + {}^* \rightleftharpoons CO_2^*$ and $CO_2^* + {}^* \rightleftharpoons CO^* + O^*$.

F. Geometries of Elementary Reaction Steps

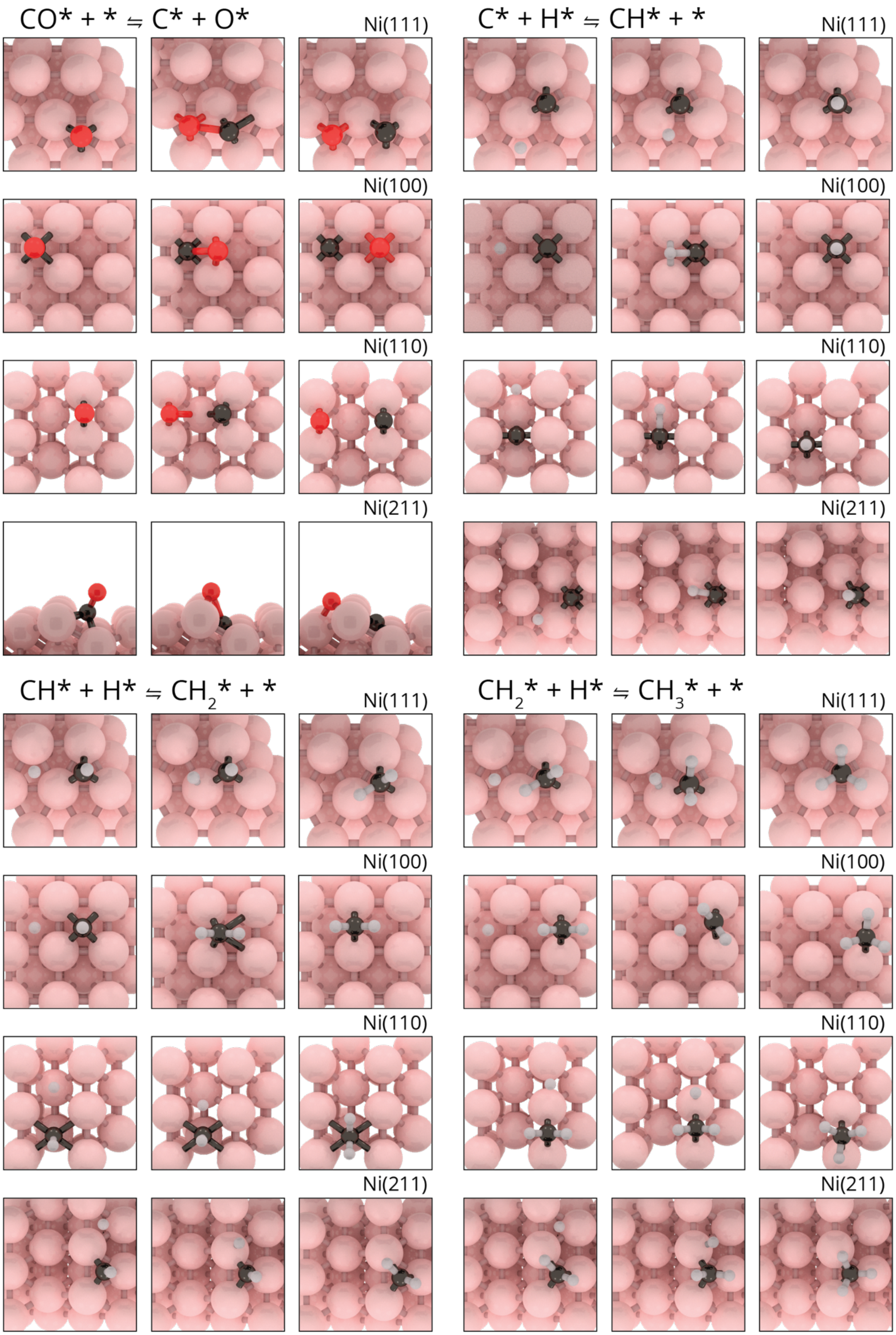

Figure S 23. Geometries of the initial, transition and final states for the elementary reactions CO + * ⇌ C* + O*, C* + H* ⇌ CH* + *, CH* + H* ⇌ CH_2* + * and CH_2* + H* ⇌ CH_3* + *.

F. Geometries of Elementary Reaction Steps

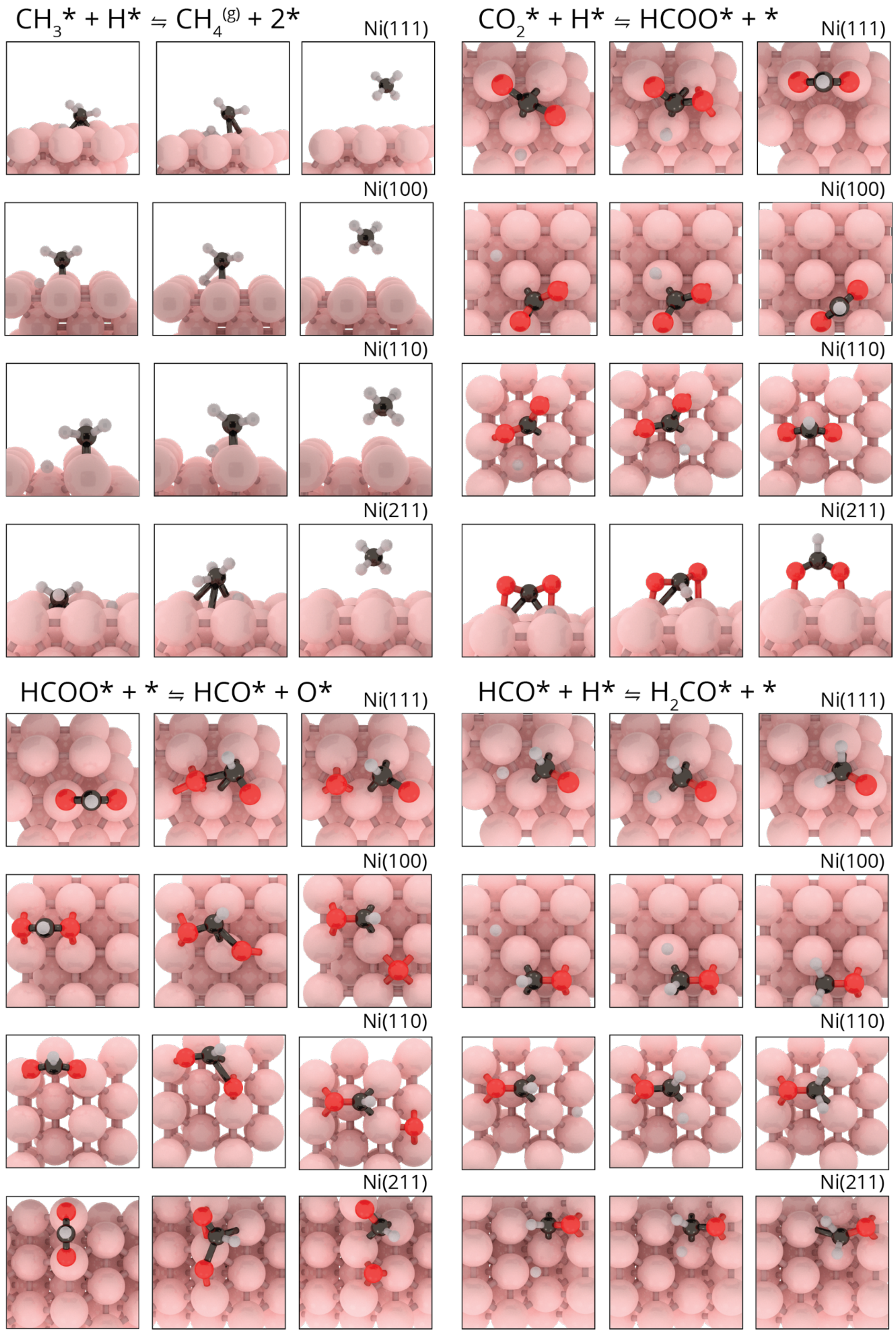

Figure S 24. Geometries of the initial, transition and final states for the elementary reactions $CH_3^* + H^* \rightleftharpoons CH_4^{(g)} + 2^*$, $CO_2^* + H^* \rightleftharpoons HCOO^* + ^*$, $HCOO^* + ^* \rightleftharpoons HCO^* + O^*$ and $HCO^* + H^* \rightleftharpoons H_2CO^* + ^*$.

F. Geometries of Elementary Reaction Steps

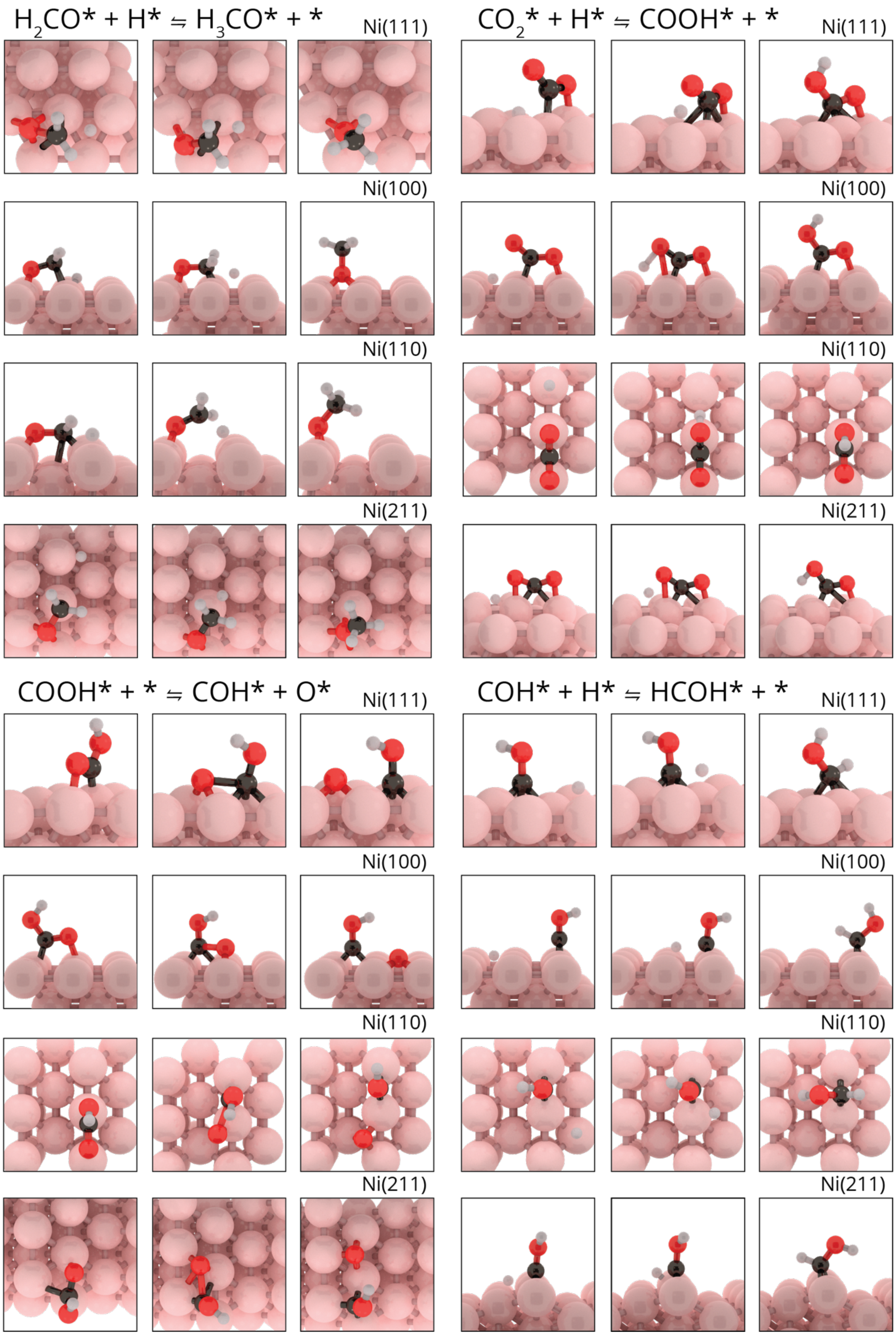

Figure S 25. Geometries of the initial, transition and final states for the elementary reactions $H_2CO^* + H^* \rightleftharpoons H_3CO^* + {}^*$, $CO_2^* + H^* \rightleftharpoons COOH^* + {}^*$, $COOH^* + {}^* \rightleftharpoons COH^* + O^*$ and $COH^* + H^* \rightleftharpoons HCOH^* + {}^*$.

F. Geometries of Elementary Reaction Steps

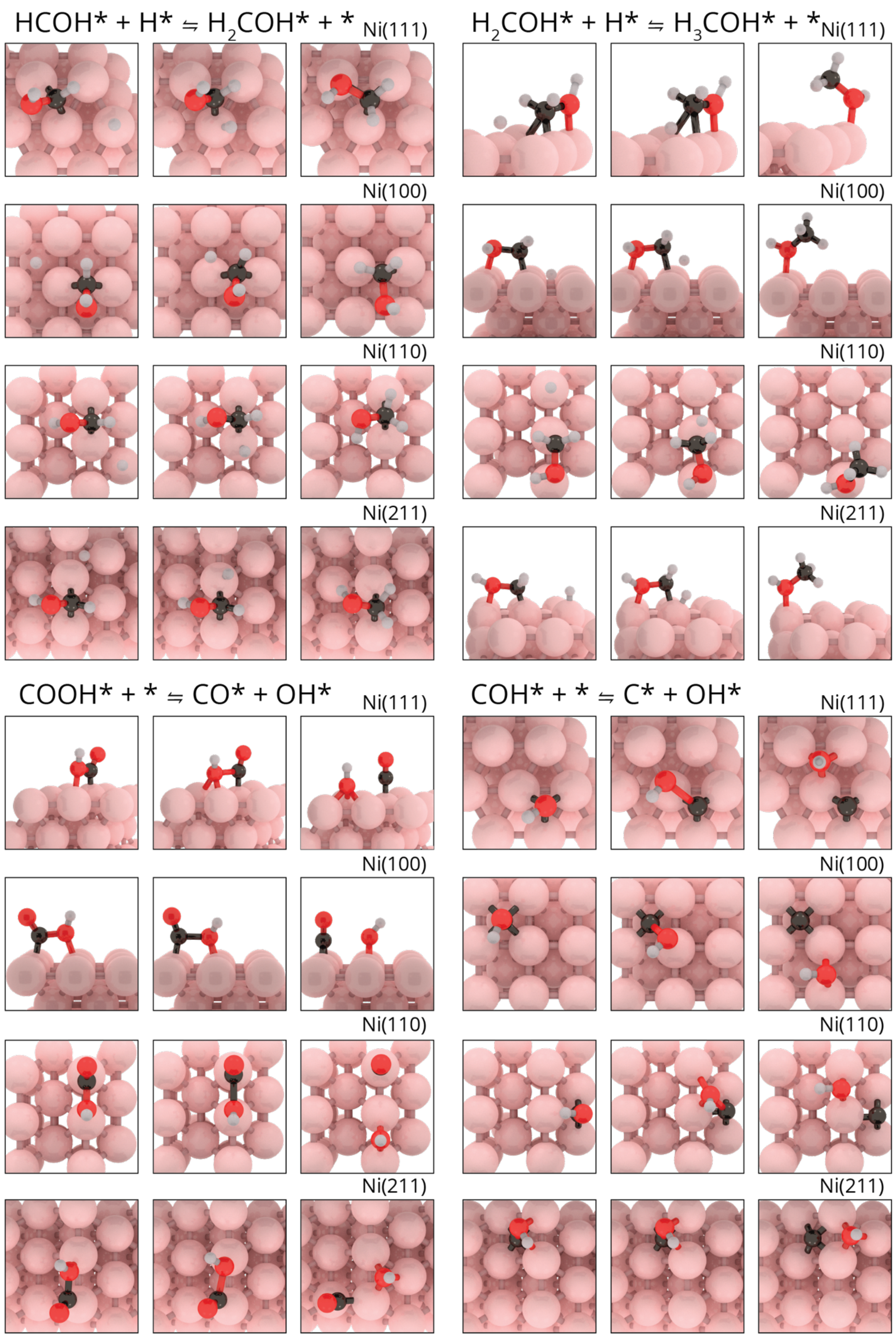

Figure S 26. Geometries of the initial, transition and final states for the elementary reactions $HCOH^* + H^* \rightleftharpoons H_2COH^* + {}^*$, $H_2COH^* + H^* \rightleftharpoons H_3COH^* + {}^*$, $COOH^* + {}^* \rightleftharpoons CO^* + OH^*$ and $COH^* + {}^* \rightleftharpoons C^* + OH^*$.

F. Geometries of Elementary Reaction Steps

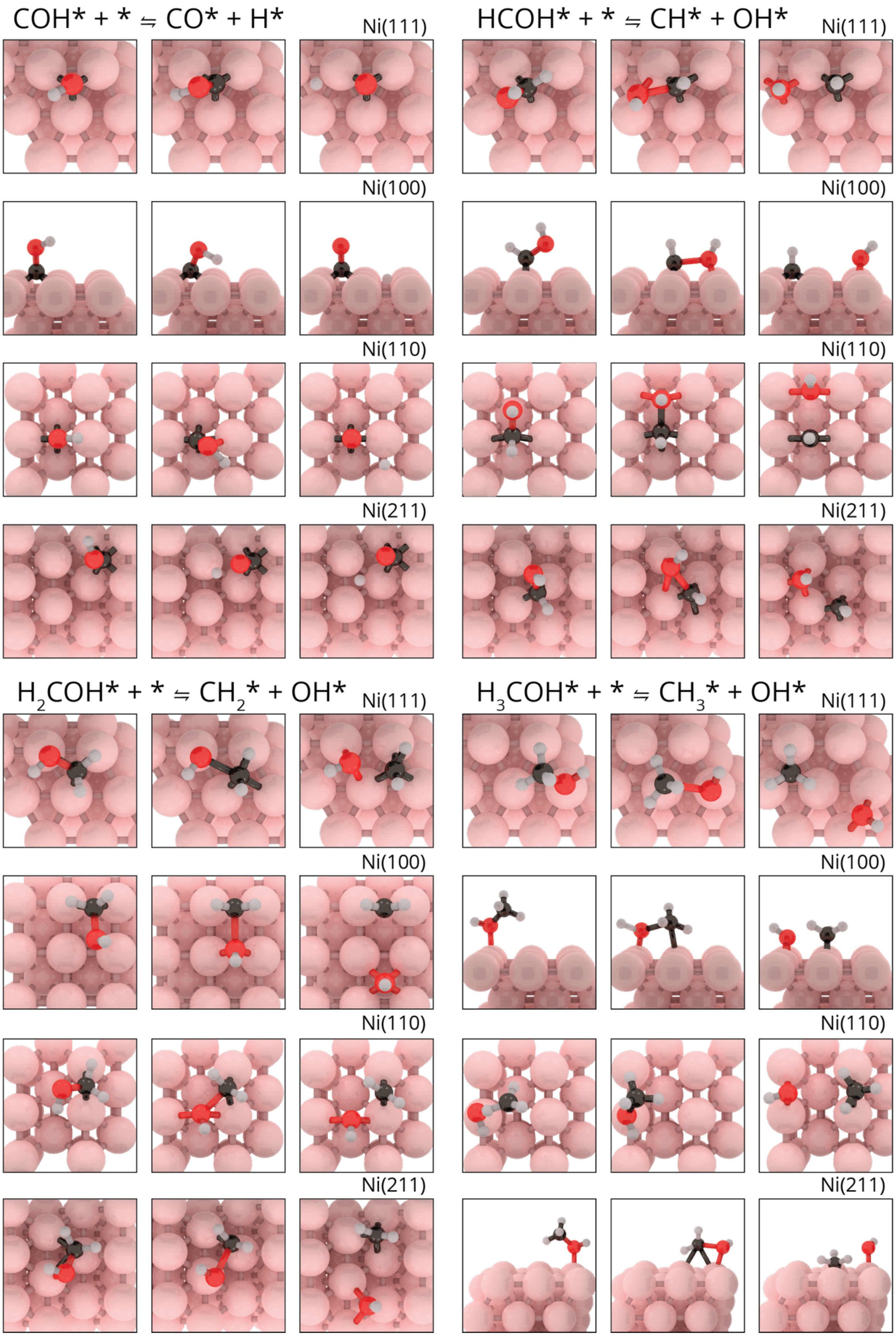

Figure S 27. Geometries of the initial, transition and final states for the elementary reactions $COH^* + ^* \rightleftharpoons CO^* + H^*$, $HCOH^* + ^* \rightleftharpoons CH^* + OH^*$, $H_2COH^* + ^* \rightleftharpoons CH_2^* + OH^*$ and $H_3COH^* + ^* \rightleftharpoons CH_3^* + OH^*$.

F. Geometries of Elementary Reaction Steps

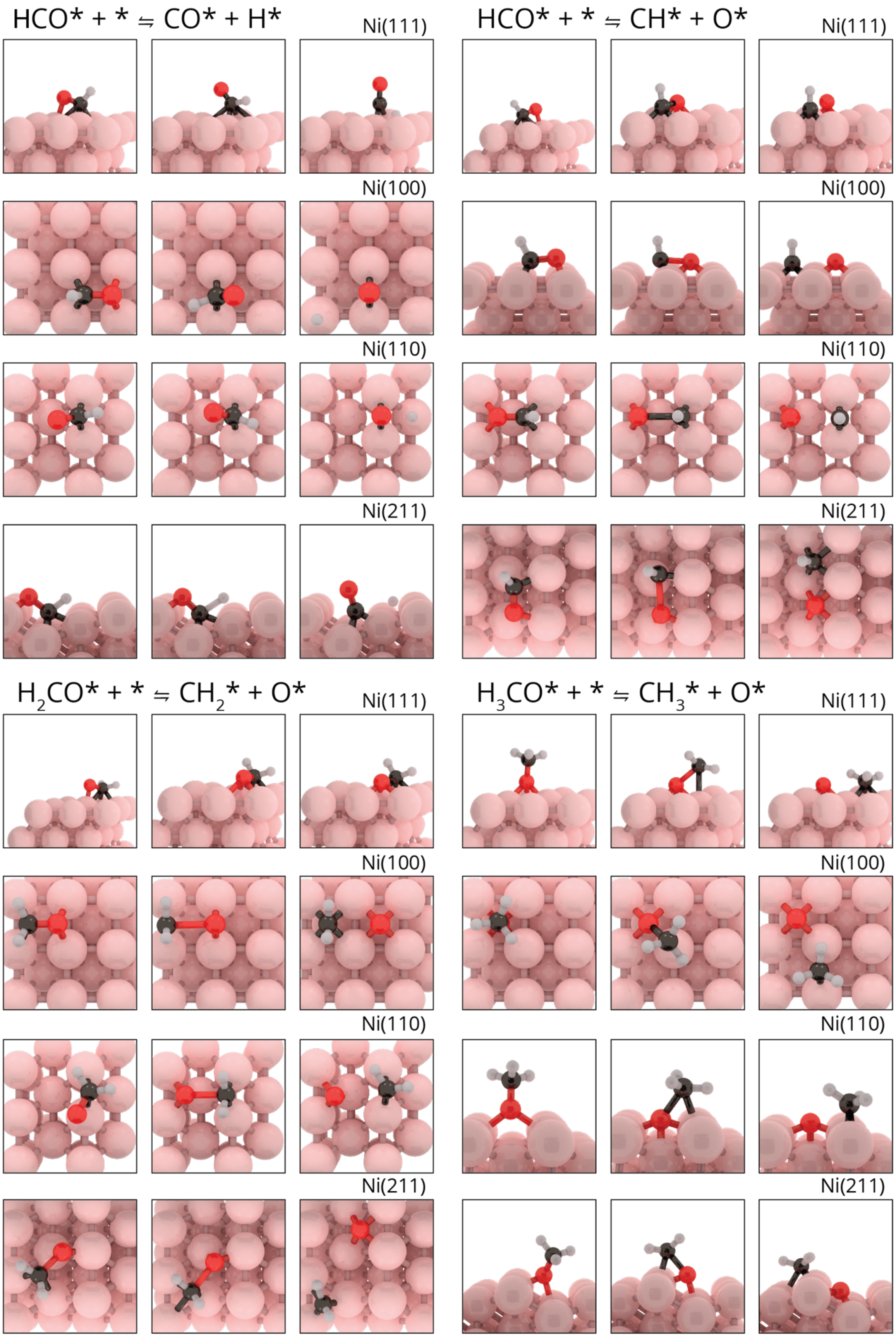

Figure S 28. Geometries of the initial, transition and final states for the elementary reactions $HCO^* + {}^* \rightleftharpoons CO^* + H^*$, $HCO^* + {}^* \rightleftharpoons CH^* + O^*$, $H_2CO^* + {}^* \rightleftharpoons CH_2^* + O^*$ and $H_3CO^* + {}^* \rightleftharpoons CH_3^* + O^*$.

F. Geometries of Elementary Reaction Steps

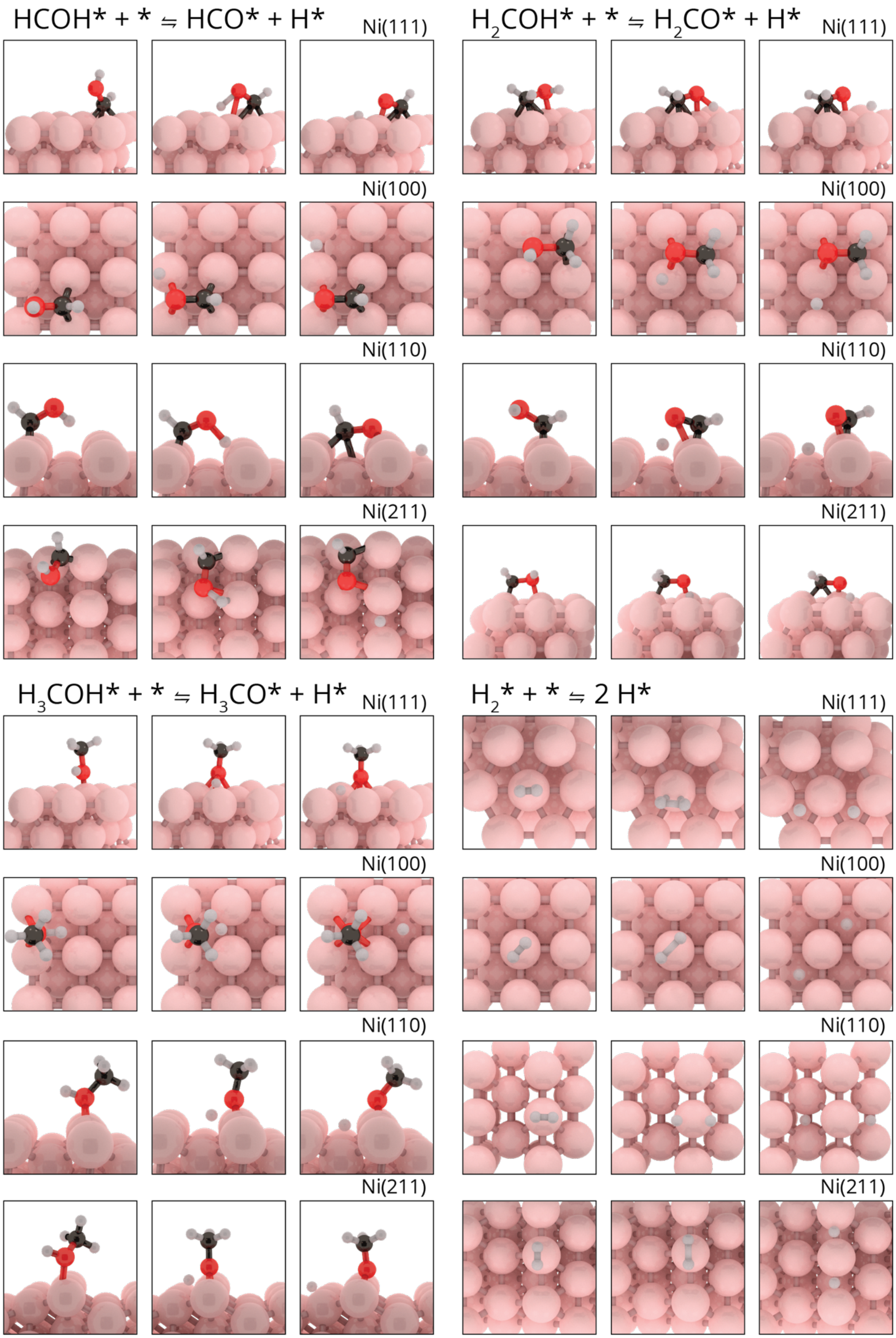

Figure S 29. Geometries of the initial, transition and final states for the elementary reactions $HCOH^* + {}^* \rightleftharpoons HCO^* + H^*$, $H_2COH^* + {}^* \rightleftharpoons H_2CO^* + H^*$, $H_3COH^* + {}^* \rightleftharpoons H_3CO^* + H^*$ and $H_2^* + {}^* \rightleftharpoons 2\,H^*$.

F. Geometries of Elementary Reaction Steps

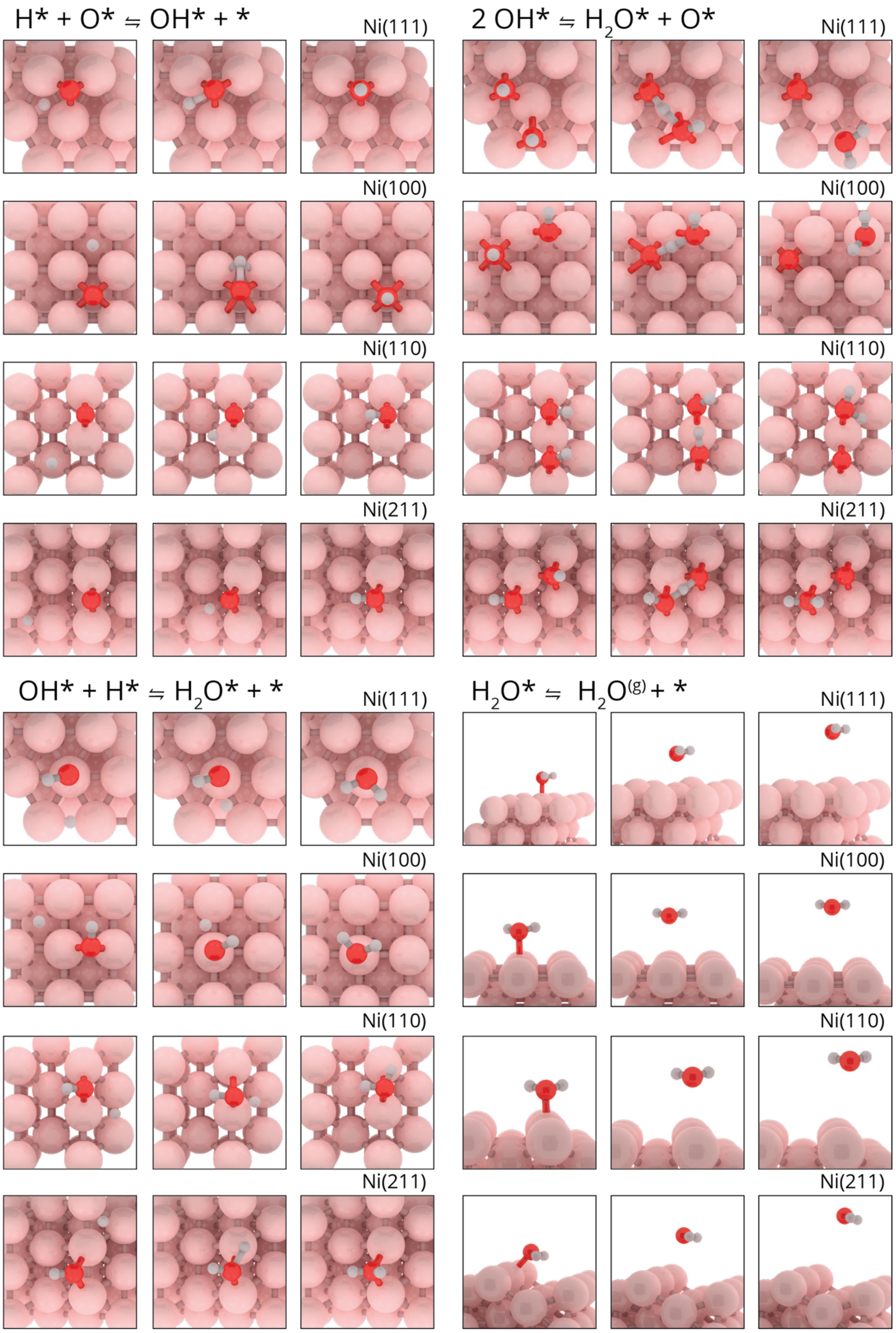

Figure S 30. Geometries of the initial, transition and final states for the elementary reactions $H^* + O^* \rightleftharpoons OH^* + {}^*$, $2\ OH^* \rightleftharpoons H_2O^* + O^*$, $OH^* + H^* \rightleftharpoons H_2O^* + {}^*$ and $H_2O^* \rightleftharpoons H_2O^{(g)} + {}^*$.

G Potential Energy Diagrams

Potential energy diagrams of the carbide-, carboxylic- and formate-pathways are given in Figure S 31 to Figure S 33. In opaque, the PED with a destabilization of 40 kJ/mol for CO^* and 20 kJ/mol for H^* intermediates. This case represents the energy landscape that has been used for the microkinetics models, as presented in the manuscript. As a comparison, a transparent line is used for the PED with zero-point-energy corrected adsorption energies and each adsorbate in their *most stable* geometries.

PED primary reaction pathways

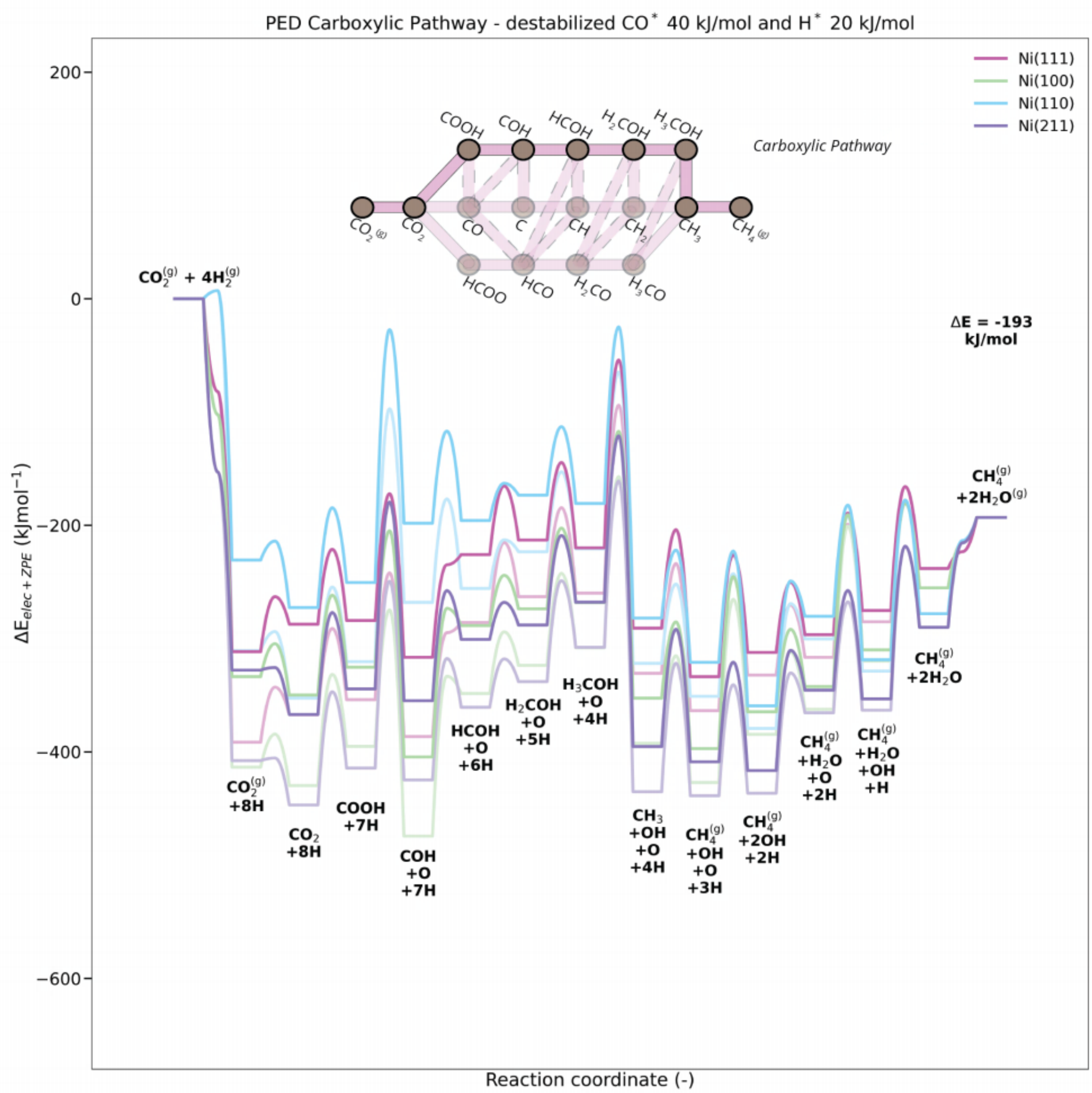

Figure S 31. Potential energy diagram (PED) of the carboxylic pathway. Opaque lines represent the PED where CO^* was destabilized with 40 kJ/mol and H^* was destabilized with 20 kJ/mol. Transparent lines represent the PED where no intermediate was destabilized.

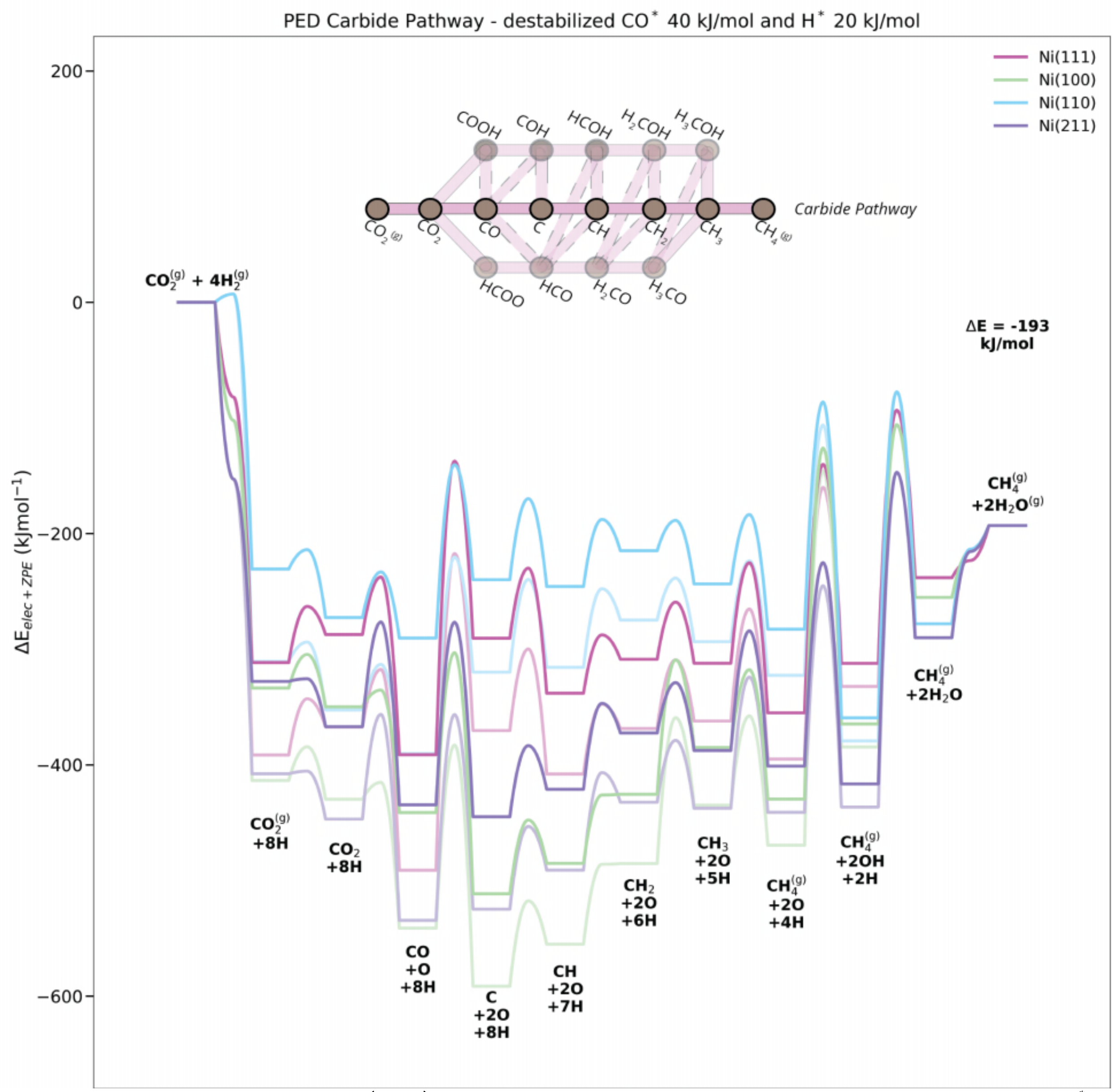

Figure S 32. Potential energy diagram (PED) of the carbide pathway. Opaque lines represent the PED where CO* was destabilized with 40 kJ/mol and H* was destabilized with 20 kJ/mol. Transparent lines represent the PED where no intermediate was destabilized.

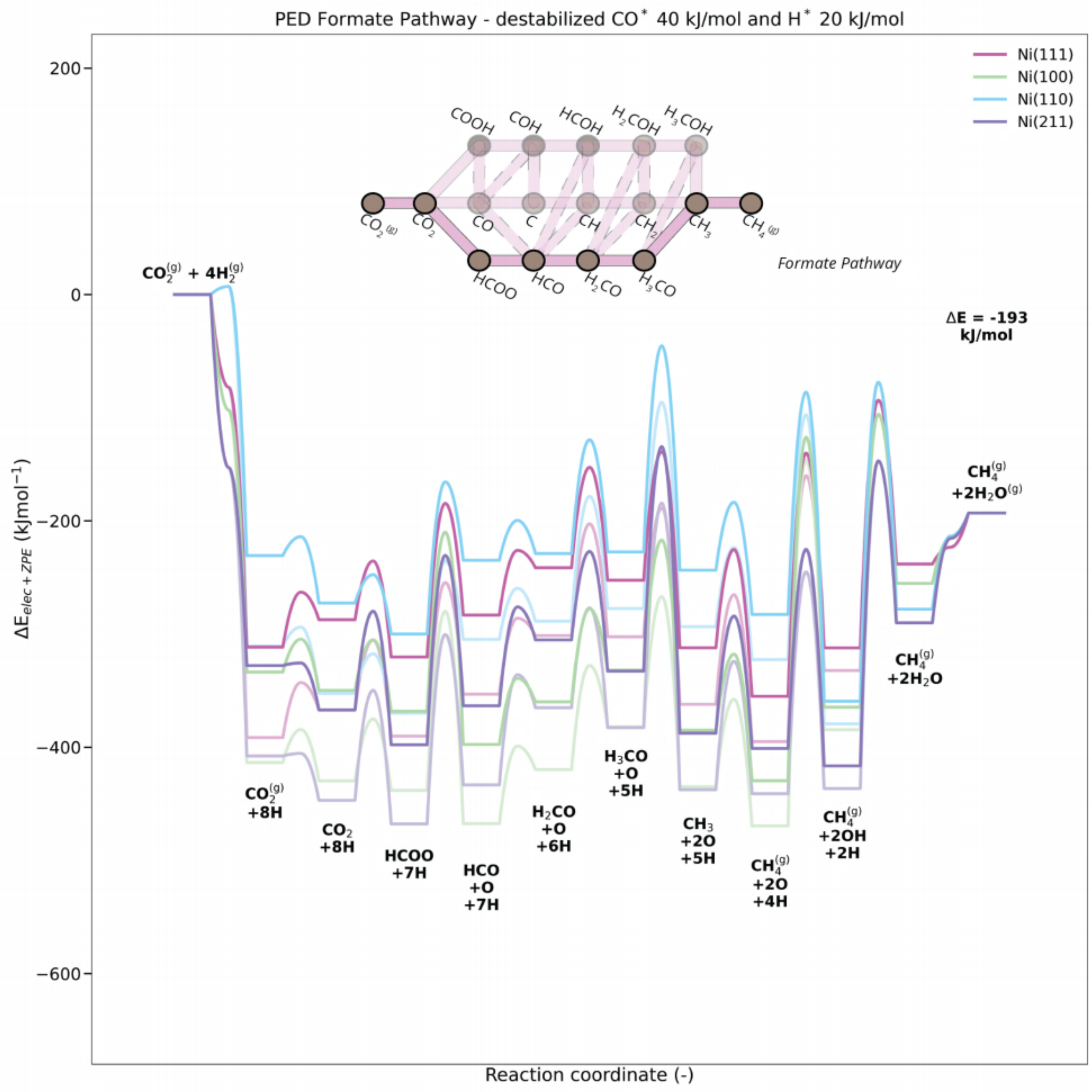

Figure S 33. Potential energy diagram (PED) of the formate pathway. Opaque lines represent the PED where CO^* was destabilized with 40 kJ/mol and H^* was destabilized with 20 kJ/mol. Transparent lines represent the PED where no intermediate was destabilized.

PED reaction flux

Potential energy diagrams of pathways with significant flux are plotted for each studied nickel facet, together with the corresponding flux diagram and a visualization of reaction intermediates in Figure S 34 to Figure S 43. In opaque, the PED with a destabilization of 40 kJ/mol for CO^* and 20 kJ/mol for H^* intermediate. This case represents the energy landscape that has been used for the microkinetics models as presented in the manuscript. As a comparison, a transparent line is used for the PED with zero-point-energy corrected adsorption energies with each adsorbate in their *most stable* geometries.

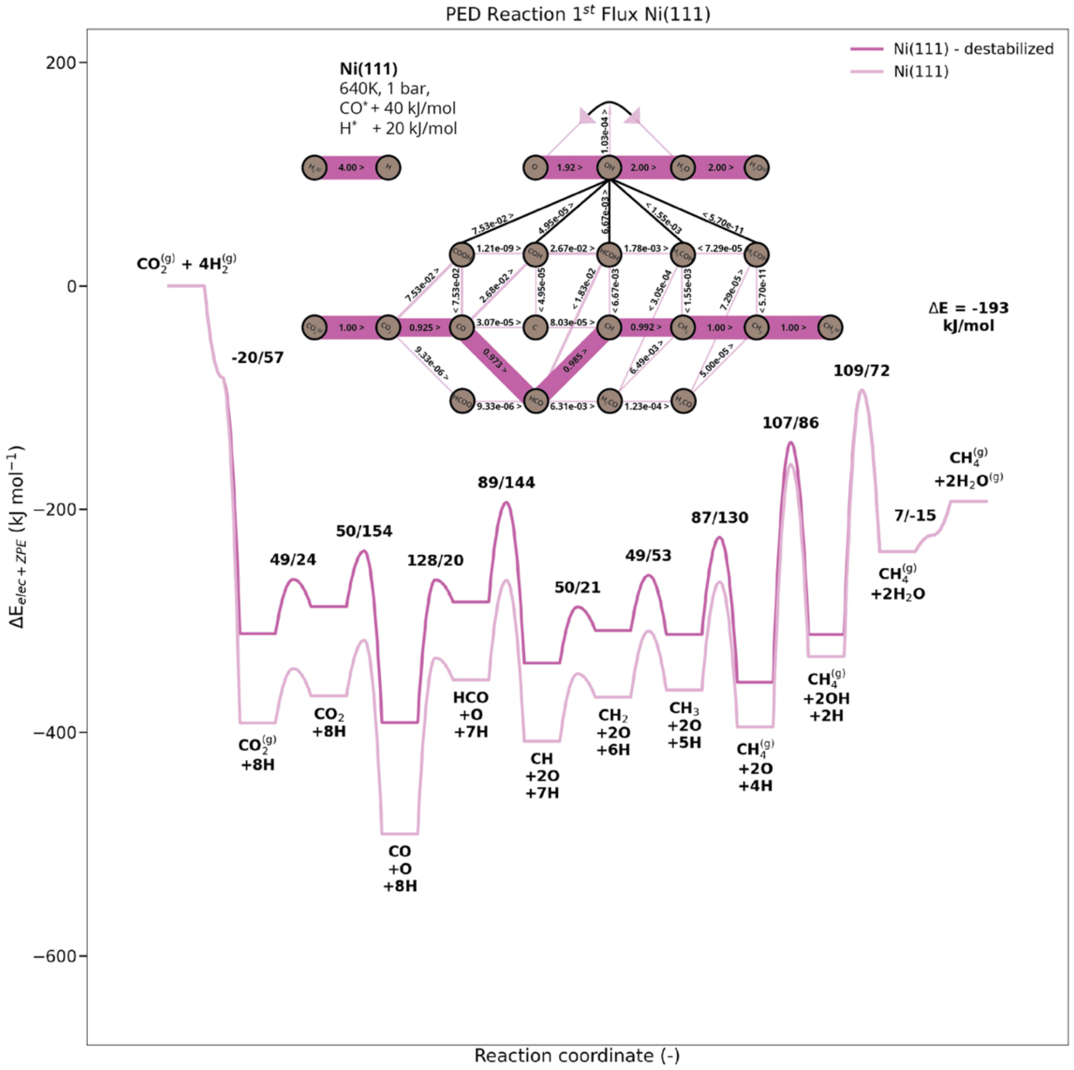

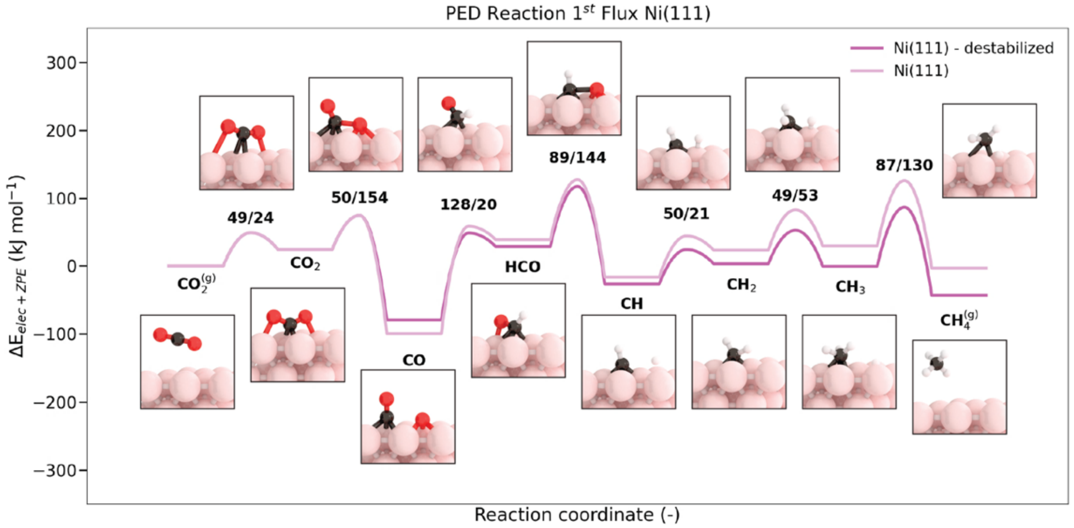

Figure S 34. Above: The complete potential energy diagram (PED) for Ni(111) corresponding to the significant flux depicted in the flux diagram. Below: PED focused on the carbonaceous part of the reaction, together with a visualization of reaction intermediates. Opaque lines represent the PED where CO^* and H^* were destabilized with 40 and 20 kJ/mol, respectively. Transparent lines represent the PED where no intermediate was destabilized.

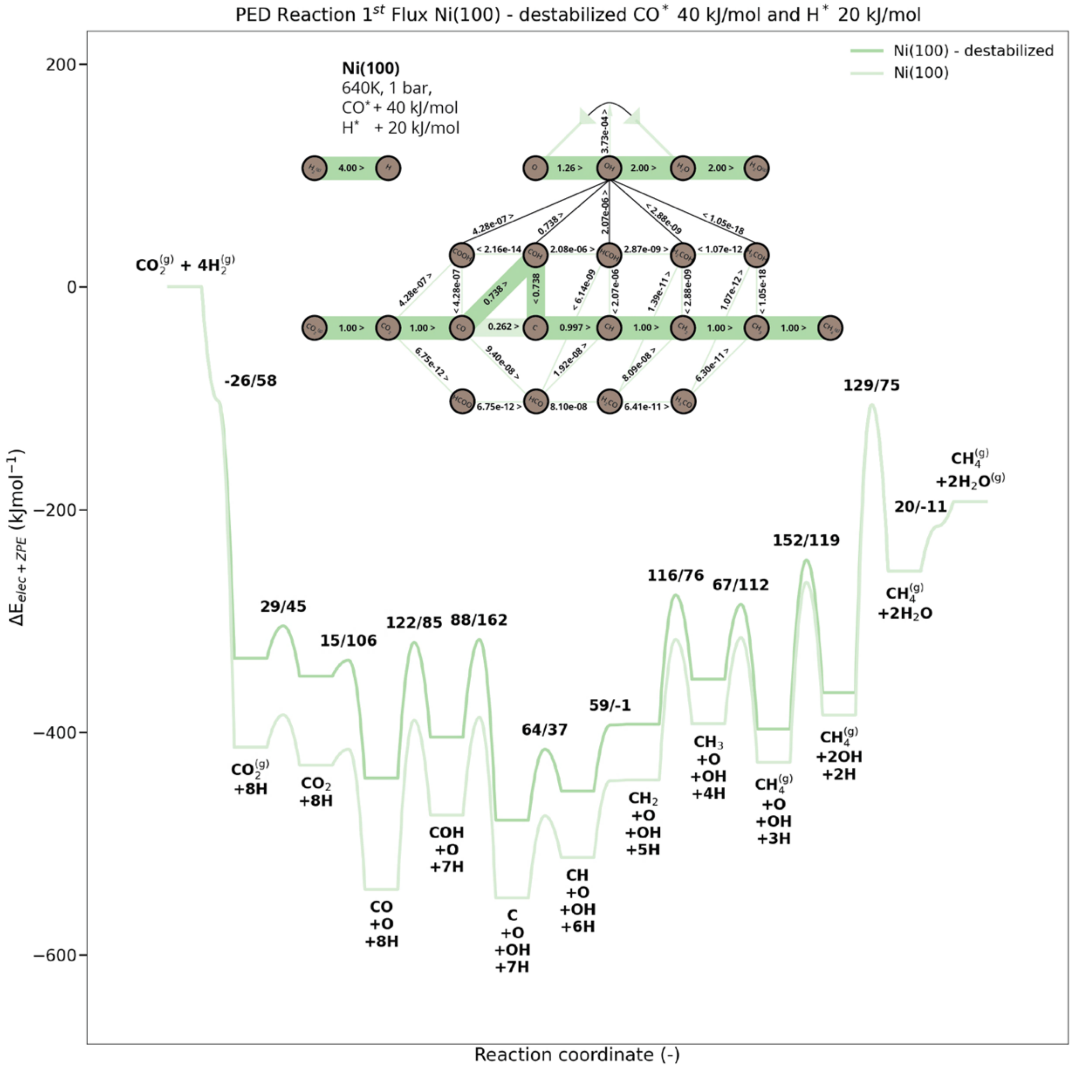

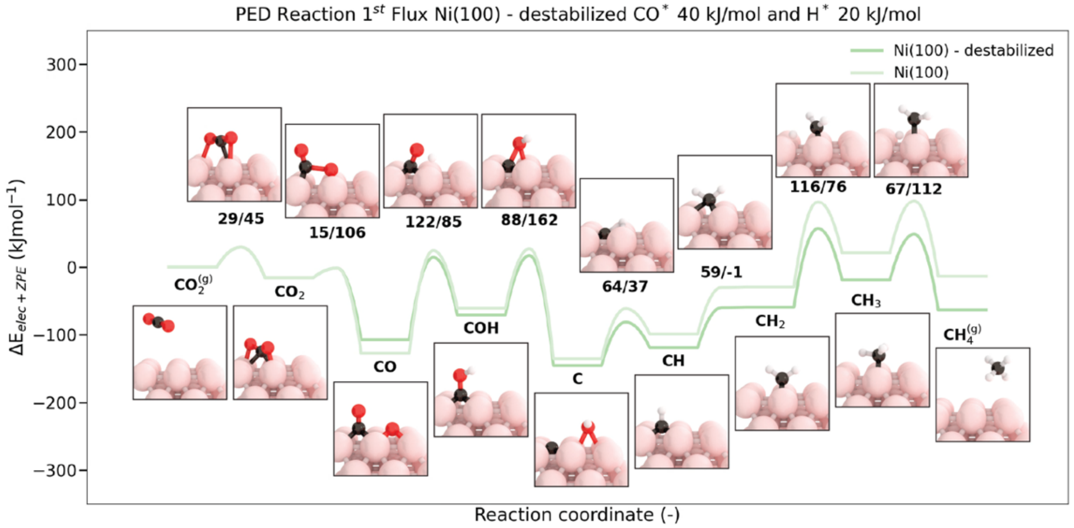

Figure S 35. Above: The complete potential energy diagram (PED) for Ni(100) corresponding to the significant flux (opaque) depicted in the flux diagram. Below: PED focused on the carbonaceous part of the reaction, together with a visualization of reaction intermediates. Opaque lines represent the PED where CO^* and H^* were destabilized with 40 and 20 kJ/mol, respectively. Transparent lines represent the PED where no intermediate was destabilized.

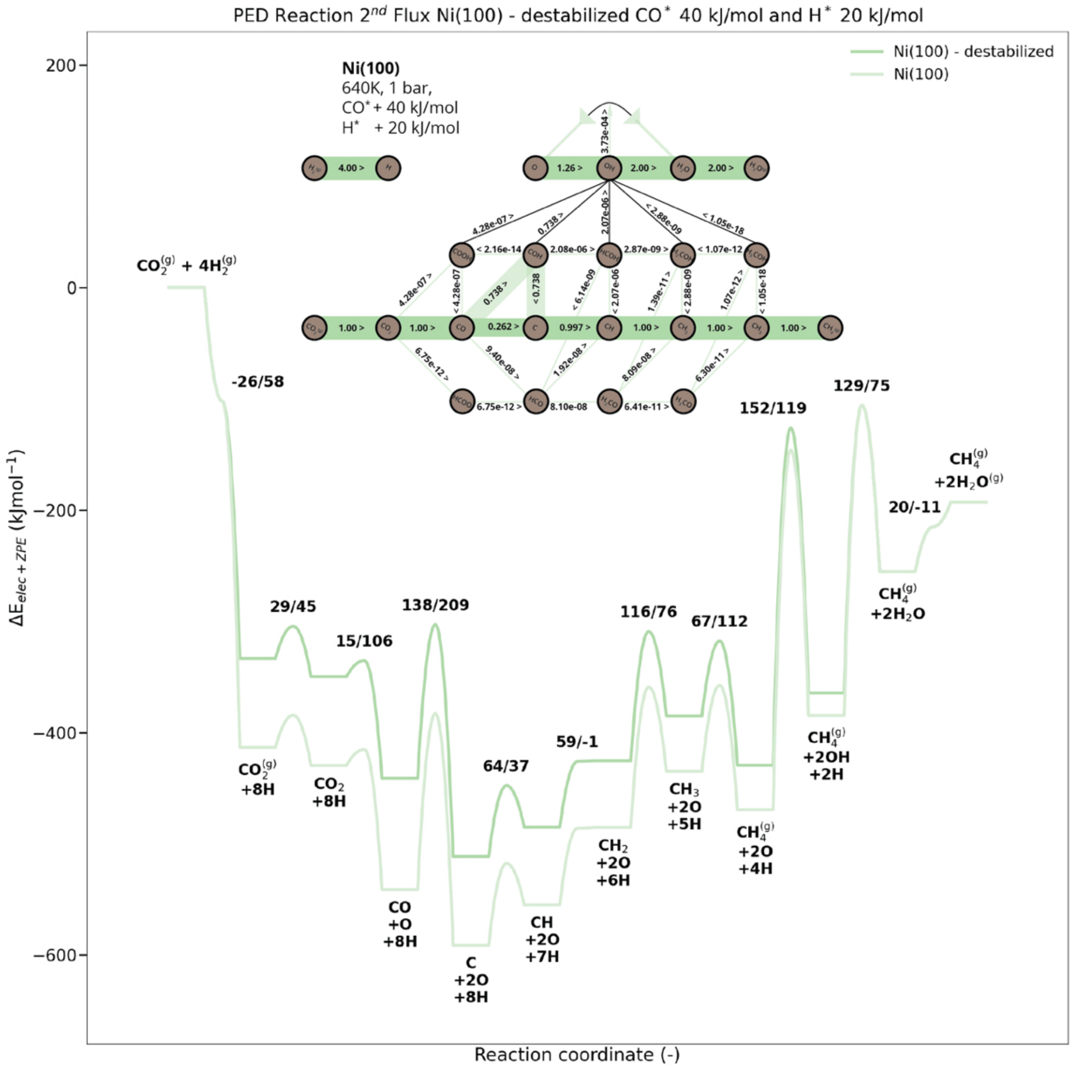

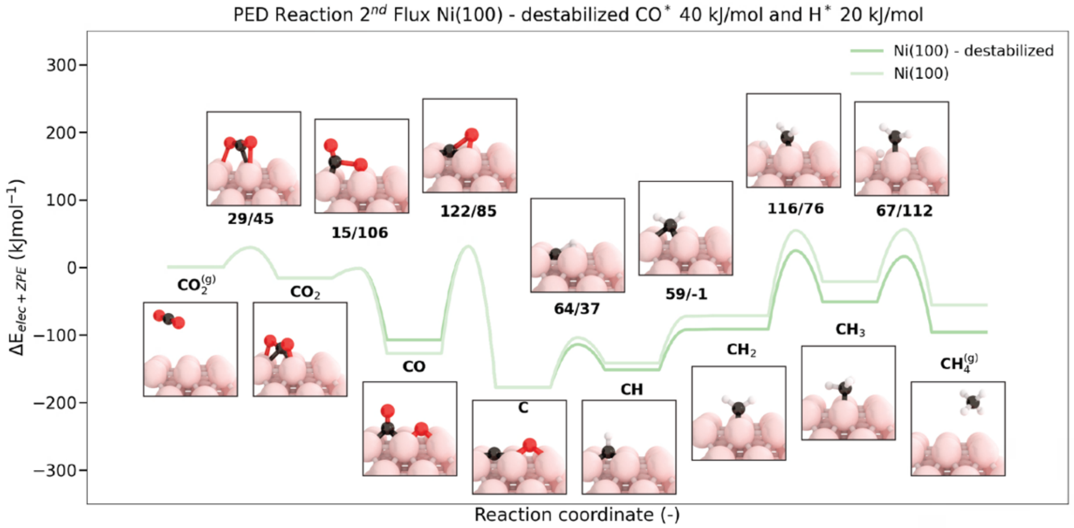

Figure S 36. Above: The complete potential energy diagram (PED) for Ni(100) corresponding to the significant flux (opaque) depicted in the flux diagram. Below: PED focused on the carbonaceous part of the reaction, together with a visualization of reaction intermediates. Opaque lines represent the PED where CO^* and H^* were destabilized with 40 and 20 kJ/mol, respectively. Transparent lines represent the PED where no intermediate was destabilized.

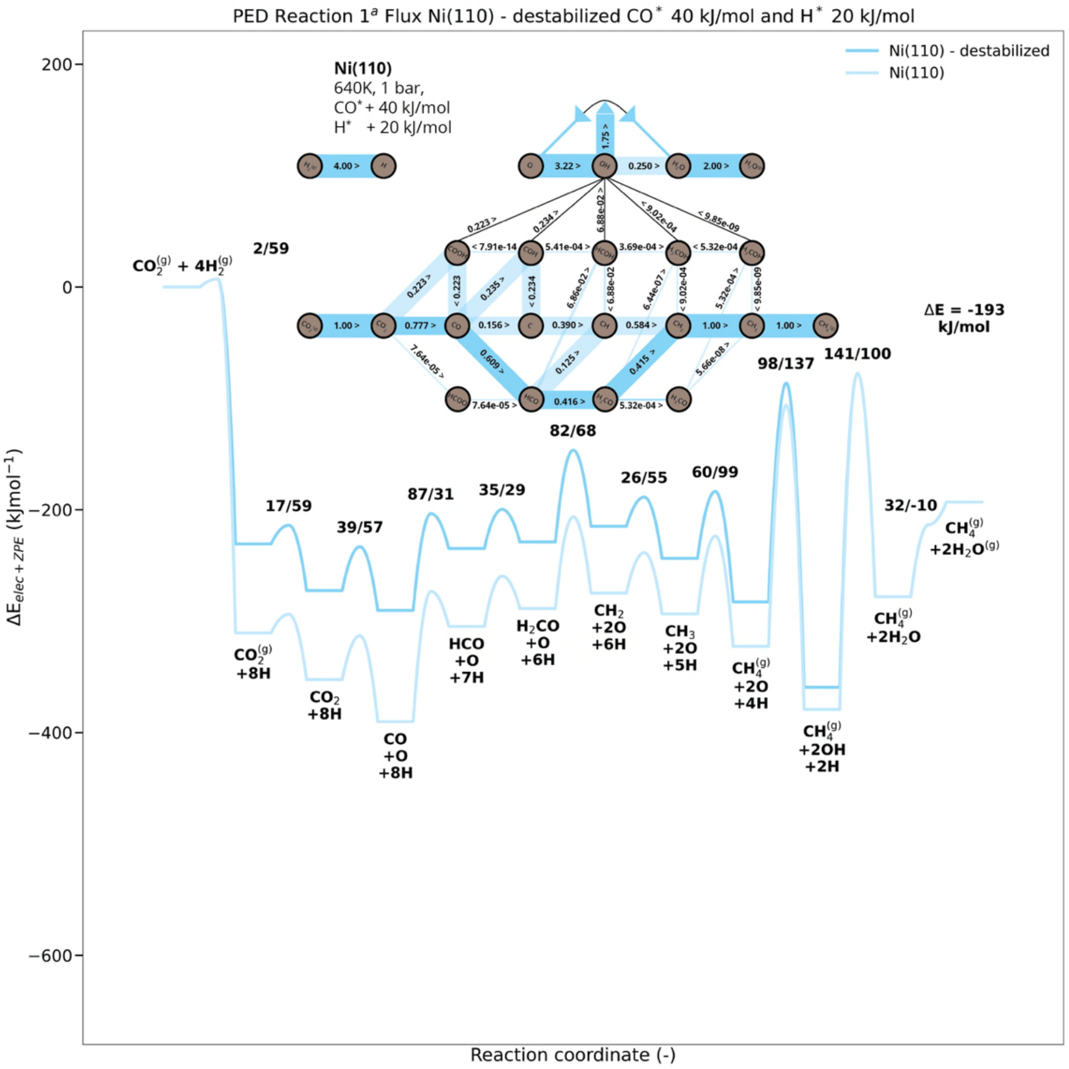

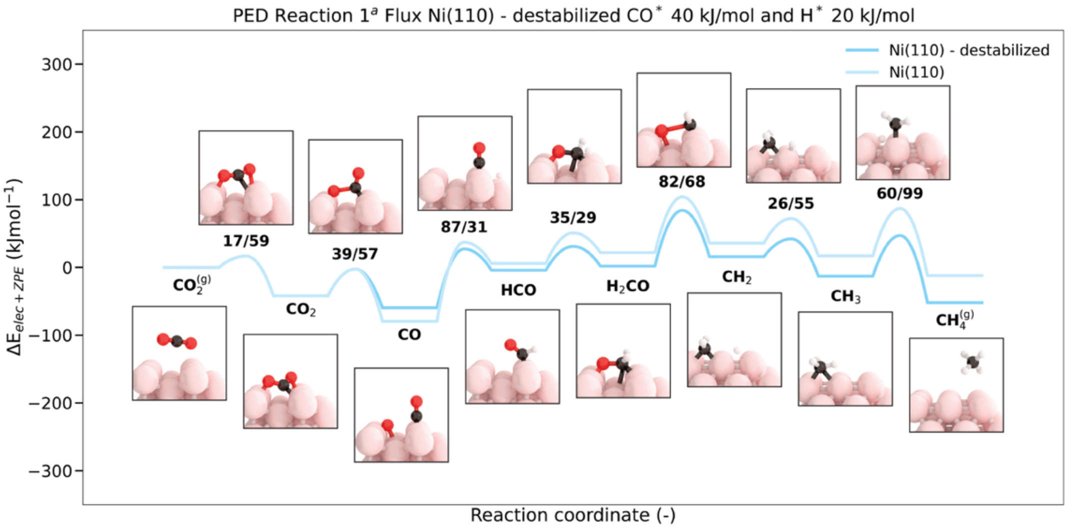

Figure S 37. Above: The complete potential energy diagram (PED) for Ni(110) corresponding to the significant flux (opaque) depicted in the flux diagram. Below: PED focused on the carbonaceous part of the reaction, together with a visualization of reaction intermediates. Opaque lines represent the PED where CO^* and H^* were destabilized with 40 and 20 kJ/mol, respectively. Transparent lines represent the PED where no intermediate was destabilized.

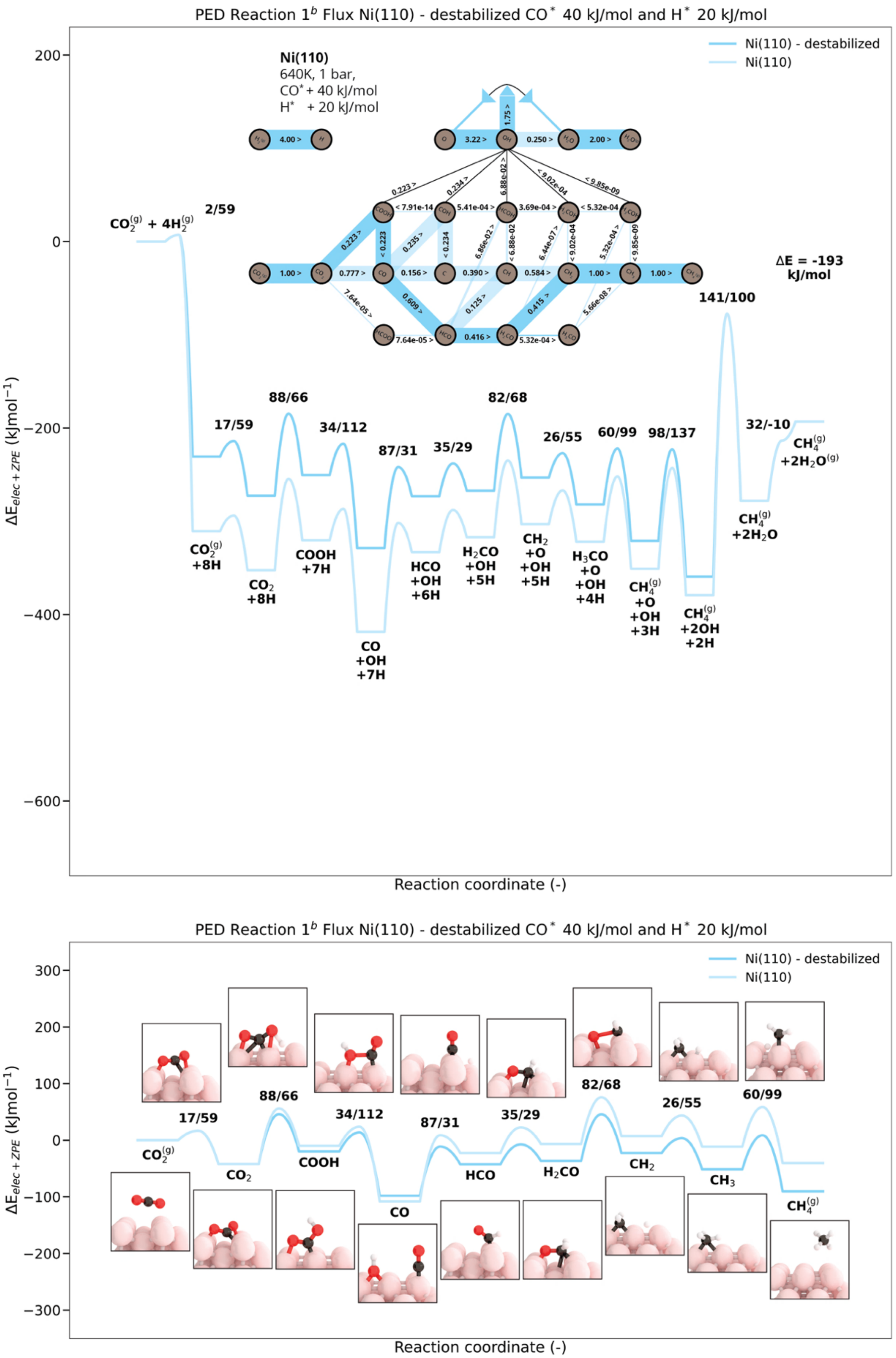

Figure S 38. Above: The complete potential energy diagram (PED) for Ni(110) corresponding to the significant flux (opaque) depicted in the flux diagram. Below: PED focused on the carbonaceous part of the reaction, together with a visualization of reaction intermediates. Opaque lines represent the PED where CO^* and H^* were destabilized with 40 and 20 kJ/mol, respectively. Transparent lines represent the PED where no intermediate was destabilized.

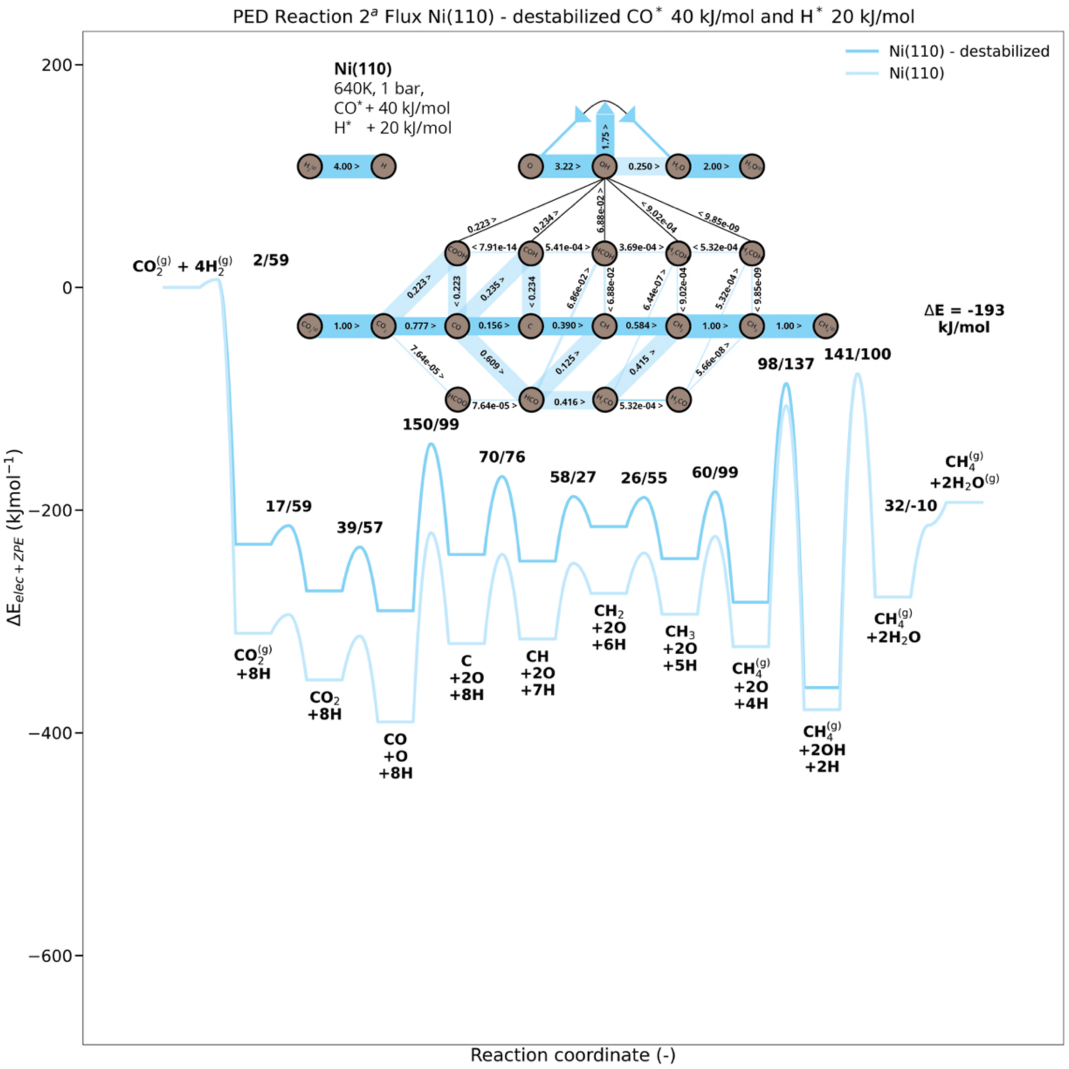

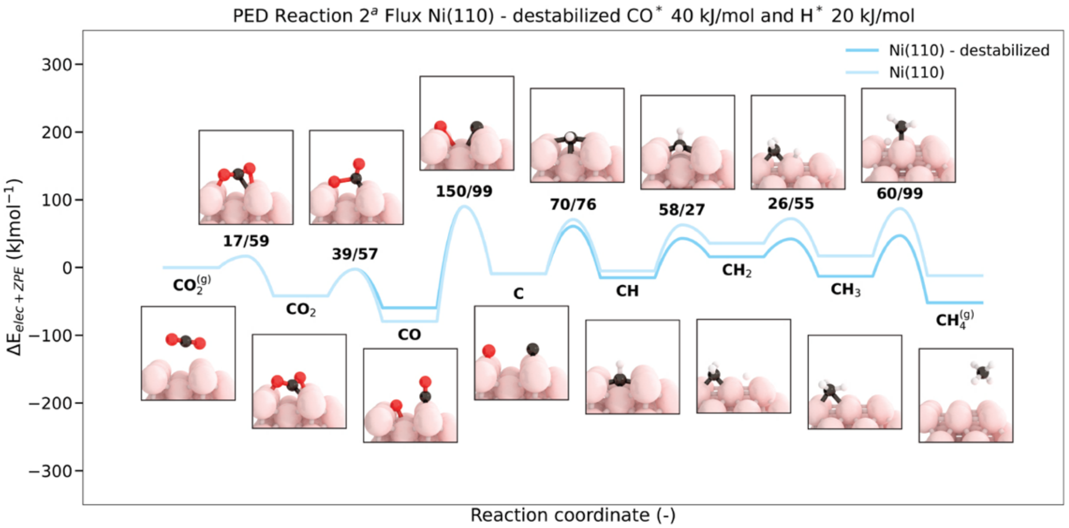

Figure S 39. Above: The complete potential energy diagram (PED) for Ni(110) corresponding to the significant flux (opaque) depicted in the flux diagram. Below: PED focused on the carbonaceous part of the reaction, together with a visualization of reaction intermediates. Opaque lines represent the PED where CO^* and H^* were destabilized with 40 and 20 kJ/mol, respectively. Transparent lines represent the PED where no intermediate was destabilized.

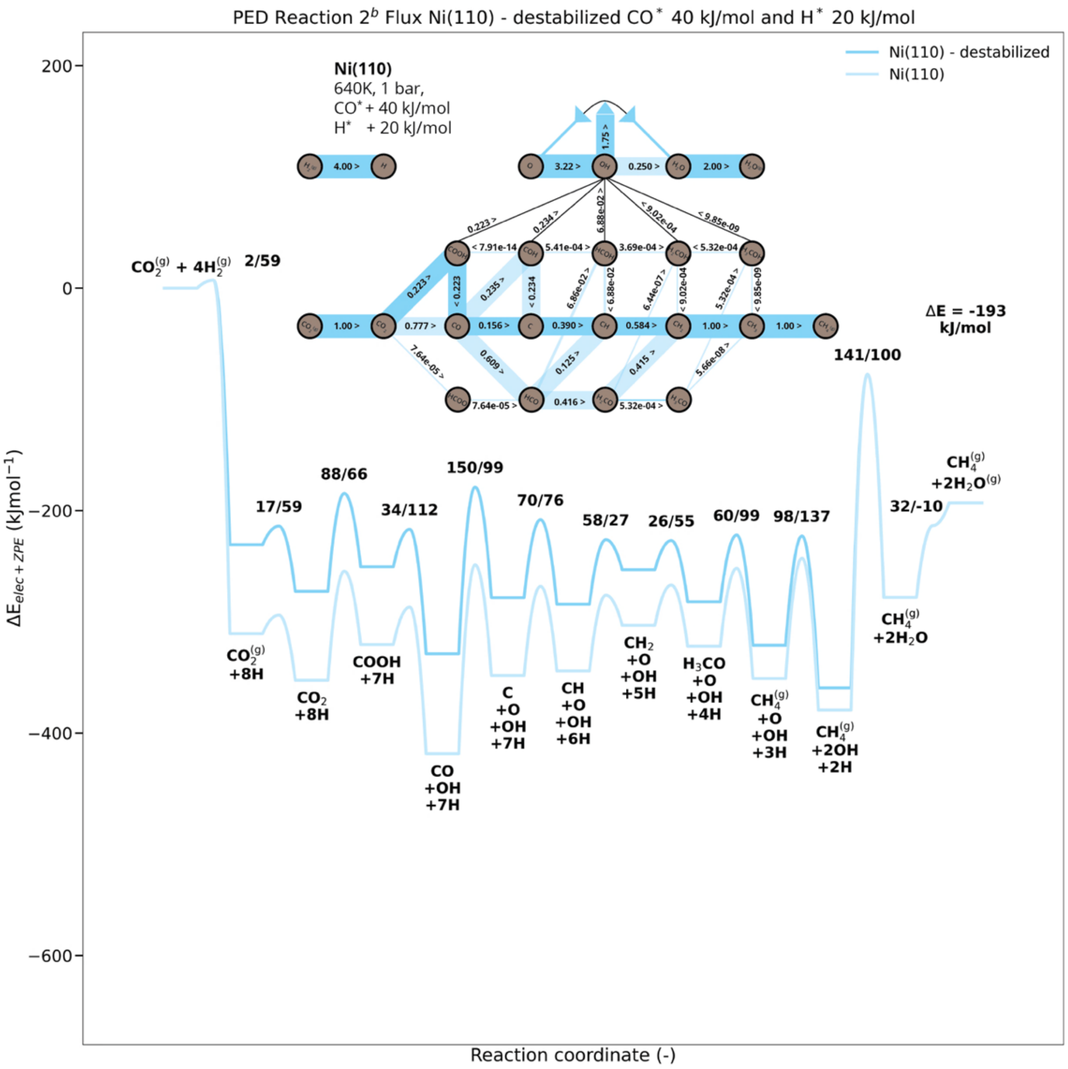

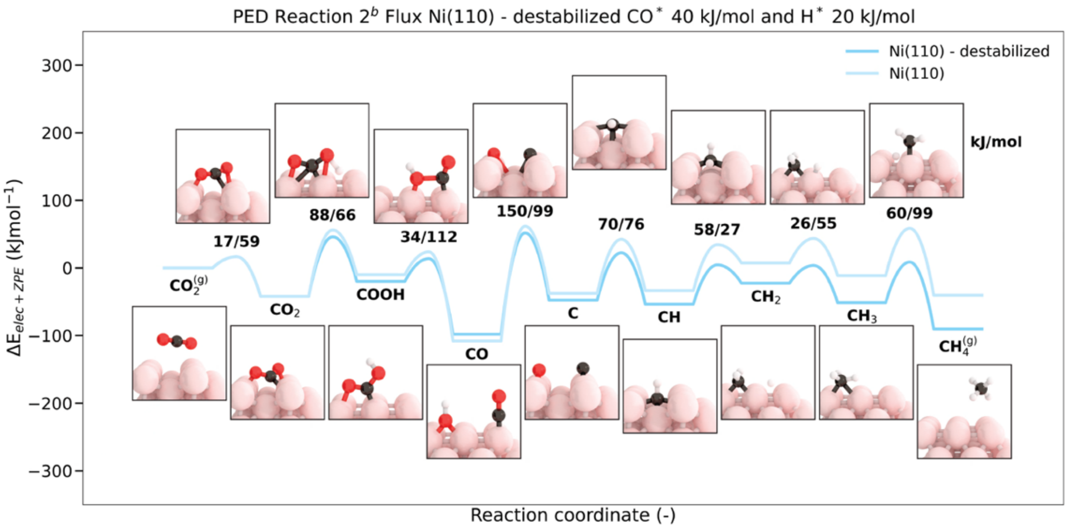

Figure S 40. Above: The complete potential energy diagram (PED) for Ni(110) corresponding to the significant flux (opaque) depicted in the flux diagram. Below: PED focused on the carbonaceous part of the reaction, together with a visualization of reaction intermediates. Opaque lines represent the PED where CO* and H* were destabilized with 40 and 20 kJ/mol, respectively. Transparent lines represent the PED where no intermediate was destabilized.

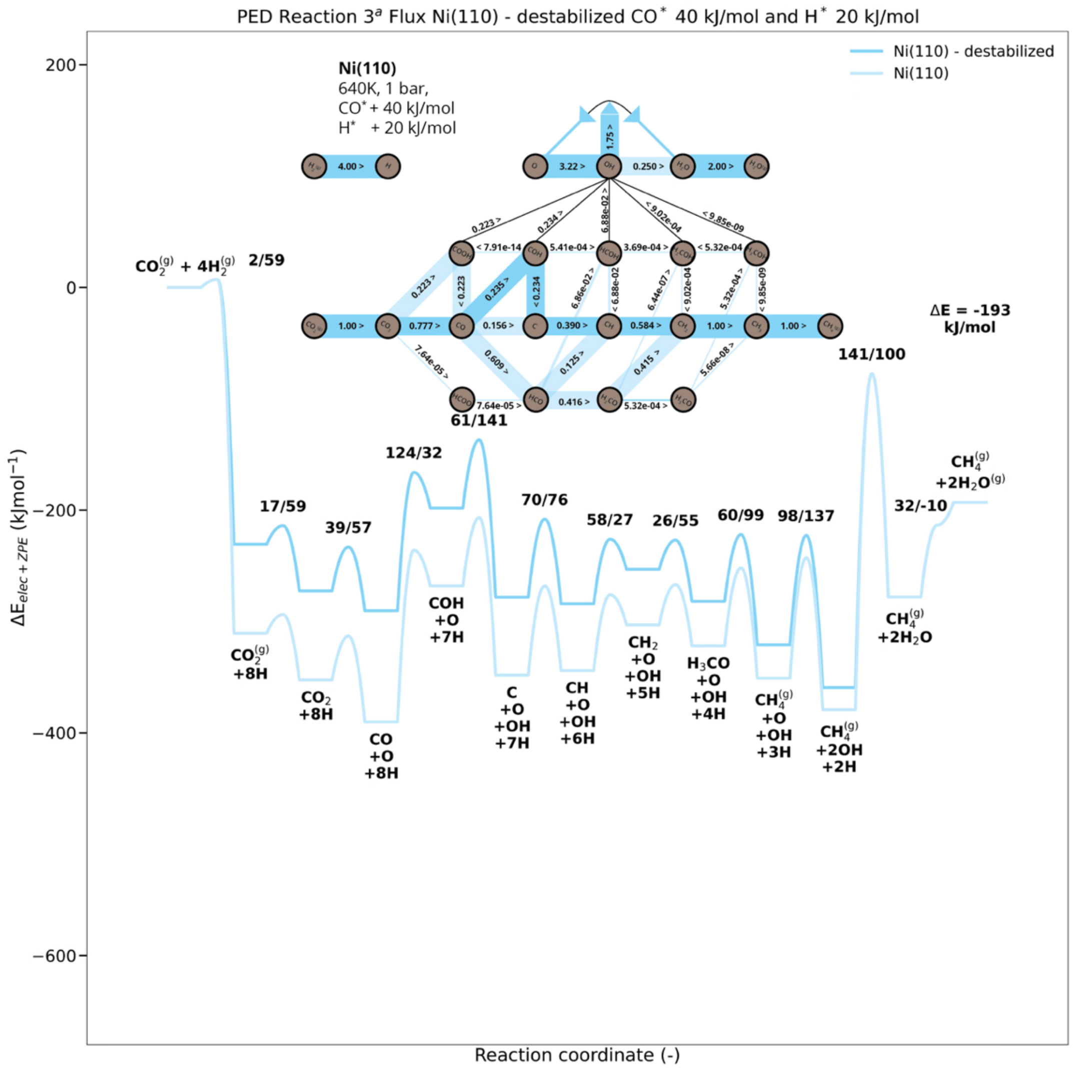

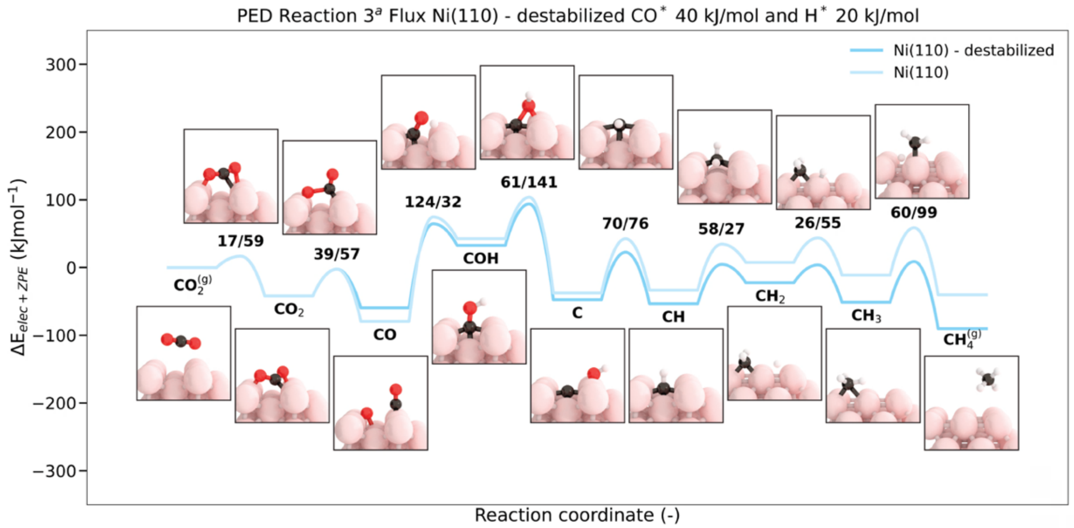

Figure S 41. Above: The complete potential energy diagram (PED) for Ni(110) corresponding to the significant flux (opaque) depicted in the flux diagram. Below: PED focused on the carbonaceous part of the reaction, together with a visualization of reaction intermediates. Opaque lines represent the PED where CO^* and H^* were destabilized with 40 and 20 kJ/mol, respectively. Transparent lines represent the PED where no intermediate was destabilized.

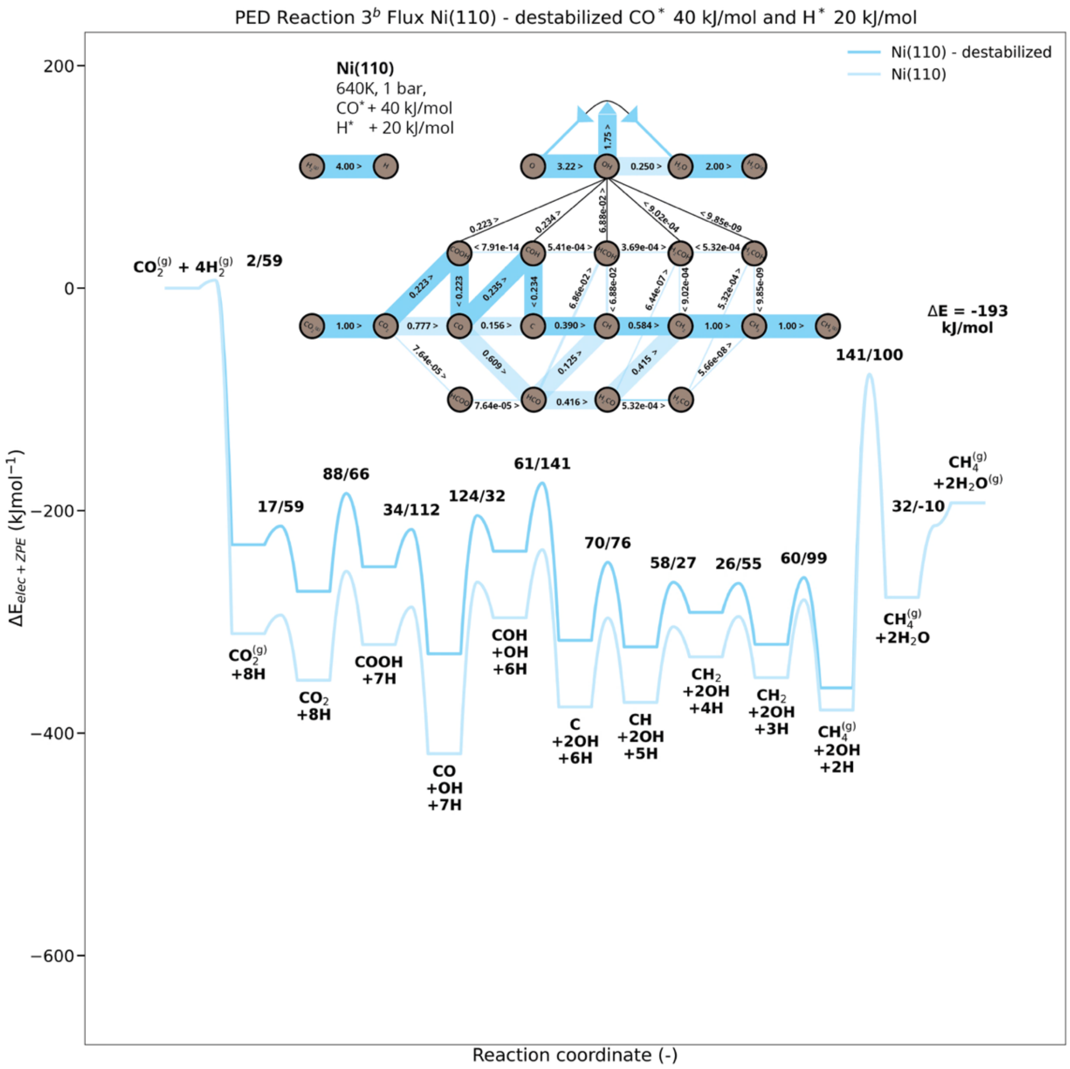

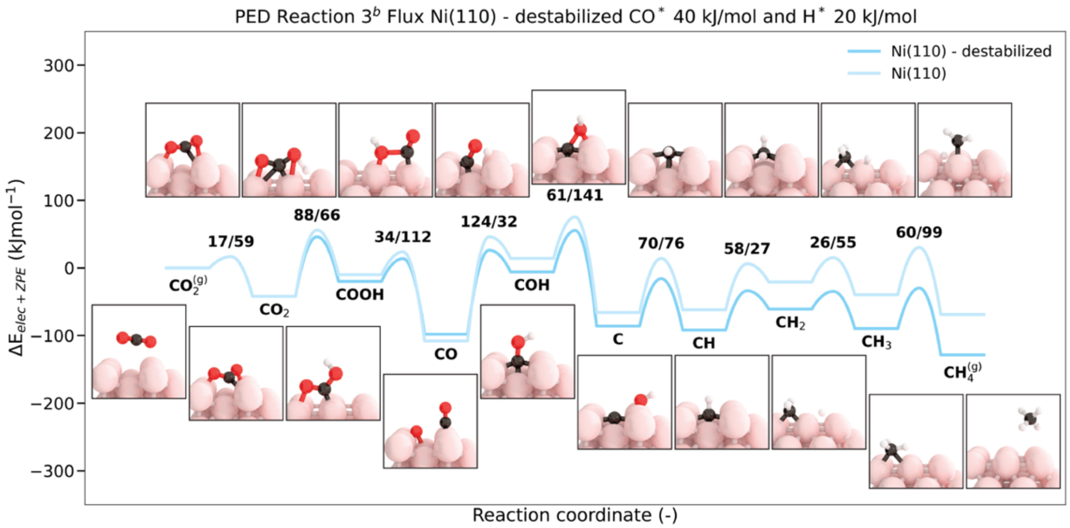

Figure S 42. Above: The complete potential energy diagram (PED) for Ni(110) corresponding to the significant flux (opaque) depicted in the flux diagram. Below: PED focused on the carbonaceous part of the reaction, together with a visualization of reaction intermediates. Opaque lines represent the PED where CO^* and H^* were destabilized with 40 and 20 kJ/mol, respectively. Transparent lines represent the PED where no intermediate was destabilized.

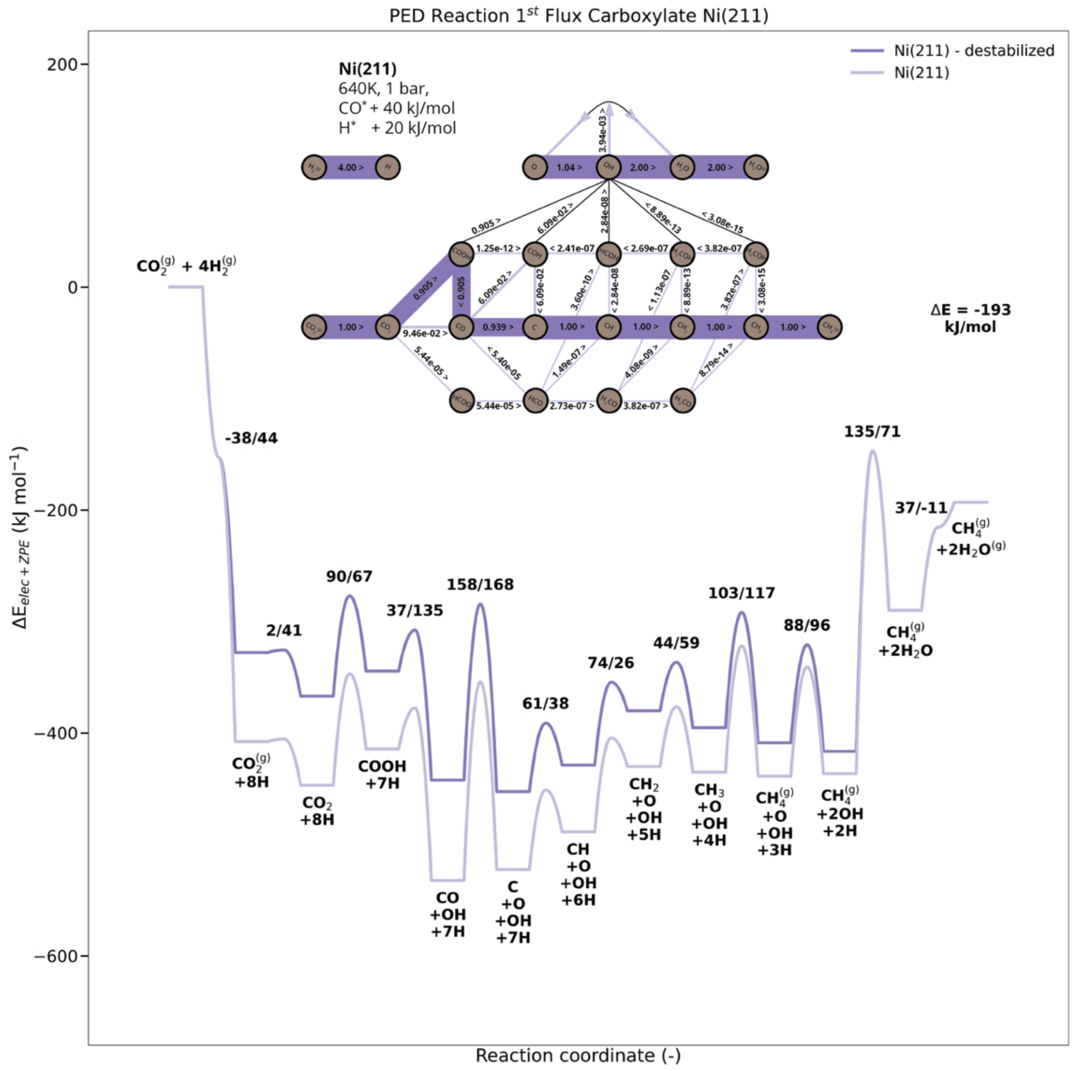

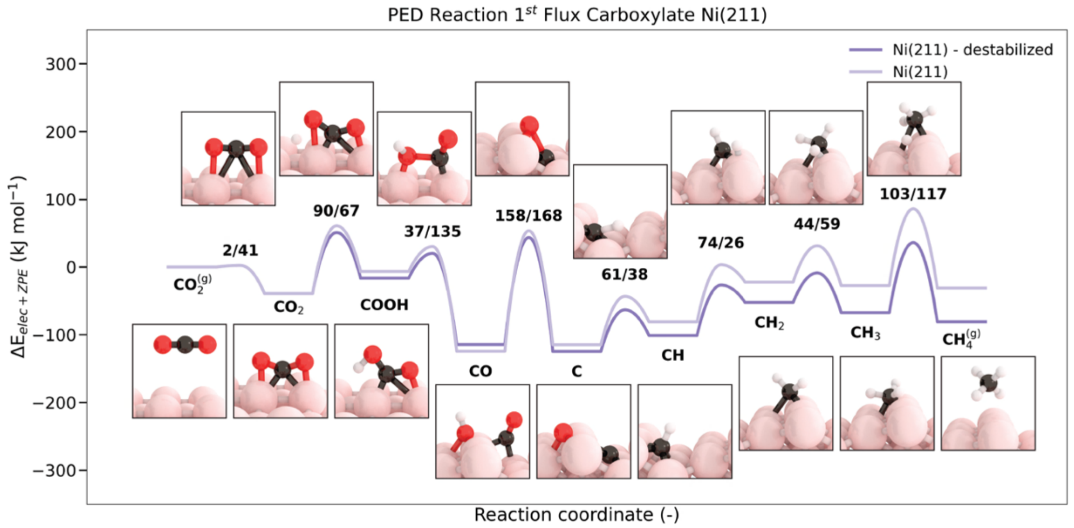

Figure S 43. Above: The complete potential energy diagram (PED) for Ni(211) corresponding to the significant flux depicted in the flux diagram. Below: PED focused on the carbonaceous part of the reaction, together with a visualization of reaction intermediates. Opaque lines represent the PED where CO^* and H^* were destabilized with 40 and 20 kJ/mol, respectively. Transparent lines represent the PED where no intermediate was destabilized.

H Microkinetics Simulations

Input

Input files for MKM simulations of CO_2 methanation over 4 nickel facets. Simulations were performed with an initial CO_2:H_2 mixture of 1:4, a total pressure of 1 bar and temperatures between 500 - 800 K. Forward and backward activation energies and vibrational partition functions (υ) at 673 K are provided for elementary reactions taking place on the catalytic surface. The forward and backward activation energies are with respect to the most stable adsorption energy, except for CO^* and H^* which were destabilized with 40 and 20 kJ/mol, respectively. All vibrational partition functions are calculated using the most stable adsorption energies of each reaction intermediate.
Necessary input values for adsorption and desorption are: surface area of the adsorption site (A), mass of the reactant (m), sticking coefficient, remaining entropy of the adsorbed intermediate ($q_{\text{vib,ads}}$), desorption energy (E_{des}), thermodynamic coefficients A-H and lateral interaction potential. The thermodynamic coefficients are listed in Table S 12.

Table S 12. Thermodynamic coefficients A-H for gaseous species.

Thermodynamic table for Shomate equation[1]				
Coefficient	CO_2	H_2	CH_4	H_2O
A	24.99735	33.066178	-0.703029	30.092
B	55.18696	-11.363417	108.4773	6.832514
C	-33.69137	11.432816	-42.52157	6.793435
D	7.948387	-2.772874	5.862788	-2.53448
E	-0.136638	-0.158558	0.678565	0.082139
F	-403.6075	-9.980797	-76.84376	-250.881
G	228.2431	172.707974	158.7163	223.3967
H	-393.5224	0	-74.8731	-241.8264

1. Chase, M.W., J. NIST-JANAF Thermochemical Tables. J. Phys. Chem. Ref. Data, Monogr. 9 1998, No. Fourth Edition, 1–1951

The lateral correction is applied on a per-atom basis i.e., the lateral interaction penalty for CO_2 is determined by the penalty of one carbon atom and two oxygen atoms:

$$E_{\text{lat}}^{\text{CO}_2} = E_{\text{lat}}^{\text{C}} + 2E_{\text{lat}}^{\text{O}} \tag{1}$$

Each per-atom contribution is exponentially dependent on the surface coverage according to:

$$E_{\text{lat}}^{\text{x}} = E_{\theta=1}^{\text{x}} \times (101^{\theta_{\text{lat}}} - 1)/100 \tag{2}$$

where $E_{\theta=1}^{\text{x}}$ is the lateral penalty for $\theta_{\text{lat}} = 1$. For $\theta_{\text{lat}} = 0$, the lateral penalty will be zero. Values of $\theta_{\text{lat}} > 1$ are allowed and will result in exponentially increasing lateral penalties. Equation 2 is

the mathematical form of "SIMPLELAT" (Table S 13). The lateral interactions were assumed to be surface independent. For a more in-depth explanation of the lateral interaction potential, the reader is referred to literature[6]. Necessary input for the lateral interaction potential is listed in Table S 13.
Input values of the microkinetics simulations for each studied nickel facet are given in Table S 14 to Table S 21.

Table S 13. Snippets of the MKMCXX input file corresponding to the lateral interaction potential

	SIMPLELAT	DIR	LB	UB	LCOV	EXCLUDE
$CO_2 + * \rightleftharpoons CO_2*$	[1*$LC+2*$LO]	0	0.25	0.75	1	*,0.5H*
$H_2 + 2* \rightleftharpoons 2H*$	[2*$LH]	0	0.25	0.75	1	*,0.5H*
$CH_4 + 2* \rightleftharpoons CH_3* + H*$	[1*$LC+4*$LH]	0	0.25	0.75	1	*,0.5H*
$H_2O + * \rightleftharpoons H_2O*$	[2*$LH+1*$LO]	0	0.25	0.75	1	*,0.5H*

```
& regex_rules_start
REGEX_VAR = $LC; 60e3
REGEX_VAR = $LO; 60e3
REGEX_VAR = $LH; 15e3
& regex_rules_end
```

6 Zijlstra, B.; Broos, R. J. P.; Chen, W.; Filot, I. A. W.; Hensen, E. J. M. First-Principles Based Microkinetic Modeling of Transient Kinetics of CO Hydrogenation on Cobalt Catalysts. *Catal. Today* **2020**, *342* (October 2018), 131–141. https://doi.org/10.1016/j.cattod.2019.03.002.

Table S 14. Input values of surface reactions for microkinetics simulations on Ni(111).

Ni(111)				
Elementary reaction	ν forw	ν backw	E act forw	E act backw
$CO_2^* + * \rightleftharpoons CO^* + O^*$	4.98E+12	1.85E+13	4.98E+04	1.54E+05
$CO^* +* \rightleftharpoons C^* + O^*$	1.54E+12	1.39E+13	2.54E+05	1.53E+05
$C^* + H^* \rightleftharpoons CH^* + *$	2.44E+13	1.70E+13	6.05E+04	1.08E+05
$CH^* + H^* \rightleftharpoons CH_2^* + *$	8.81E+12	3.90E+12	5.04E+04	2.10E+04
$CH_2^* + H^* \rightleftharpoons CH_3^* + *$	2.83E+13	1.45E+13	4.94E+04	5.29E+04
$CO_2^* + H^* \rightleftharpoons COOH^* + *$	1.71E+13	2.02E+13	6.61E+04	6.29E+04
$COOH^* + * \rightleftharpoons COH^* + O^*$	4.60E+12	9.07E+12	1.12E+05	1.44E+05
$COH^* + H^* \rightleftharpoons HCOH^* + *$	1.47E+13	6.19E+12	8.17E+04	-8.99E+03
$HCOH^* + H^* \rightleftharpoons H_2COH^*$	2.76E+13	5.08E+13	6.06E+04	4.78E+04
$H_2COH^* + H^* \rightleftharpoons H_3COH^* + *$	1.01E+13	3.96E+11	6.85E+04	7.53E+04
$CO_2^* + H^* \rightleftharpoons HCOO^* + *$	5.53E+12	9.16E+12	5.19E+04	8.49E+04
$HCOO^* + * \rightleftharpoons HCO^* + O^*$	3.60E+12	1.09E+13	1.36E+05	9.87E+04
$HCO^* + H^* \rightleftharpoons H_2CO^* + *$	1.30E+13	5.85E+12	5.72E+04	1.54E+04
$H_2CO^* + H^* \rightleftharpoons H_3CO^* + *$	8.36E+12	4.25E+12	8.87E+04	9.97E+04
$COOH^* + * \rightleftharpoons CO^* + OH^*$	2.22E+13	2.42E+13	2.89E+04	1.15E+05
$COH^* + * \rightleftharpoons CO^* + H^*$	4.26E+12	6.80E+12	7.63E+04	1.51E+05
$COH^* + * \rightleftharpoons C^* + OH^*$	3.49E+12	1.74E+13	1.80E+05	1.32E+05
$HCOH^* + * \rightleftharpoons CH^* + OH^*$	2.62E+12	2.17E+13	6.27E+04	1.53E+05
$H_2COH^* + * \rightleftharpoons CH_2^* + OH^*$	7.87E+12	1.57E+13	5.65E+04	1.31E+05
$H_3COH^* + * \rightleftharpoons CH_3^* + OH^*$	3.19E+12	8.38E+13	1.66E+05	2.37E+05
$HCO^* + * \rightleftharpoons CH^* + O^*$	3.10E+12	1.43E+13	8.92E+04	1.44E+05
$HCO^* + * \rightleftharpoons CO^* + H^*$	6.43E+12	4.75E+12	1.99E+04	1.28E+05
$H_2CO^* + * \rightleftharpoons CH_2^* + O^*$	2.22E+12	1.02E+13	7.30E+04	1.40E+05
$H_3CO^* + * \rightleftharpoons CH_3^* + O^*$	5.93E+12	2.75E+13	1.14E+05	1.73E+05
$HCO^* + H^* \rightleftharpoons HCOH^* + *$	2.45E+13	4.75E+12	1.04E+05	4.63E+04
$H_2CO^* + H^* \rightleftharpoons H_2COH^*$	1.03E+13	8.21E+12	6.60E+04	3.75E+04
$H_3CO^* + H^* \rightleftharpoons H_3COH^*$	2.22E+13	1.36E+12	1.01E+05	6.88E+04
$H^* + O^* \rightleftharpoons OH^* + *$	2.81E+13	9.77E+12	1.07E+05	8.60E+04
$OH^* + H^* \rightleftharpoons H_2O^* + *$	1.45E+13	3.18E+11	1.09E+05	7.24E+04
$OH^* + OH^* \rightleftharpoons H_2O^* + O^*$	1.32E+13	8.36E+11	6.18E+04	4.62E+04

Table S 15. Input values of adsorption and desorption reactions for microkinetics simulations on Ni(111). For A-H and Lat. see Table S 12 and Table S 13, respectively.

Ni(111)							
Elementary reaction	Lat.	A-H	A (m^2)	m (a.u.)	Stick. Coëff.	Q_{vib}(-)	E_{des}(J/mol)
$CO_2 + * \rightleftharpoons CO_2^*$	...	...	2.68E-20	44	0.01	9.729	-2.44E+04
$H_2 + 2^* \rightleftharpoons 2H^*$	...	...	5.37E-20	2	0.05	2.353	7.74E+04
$CH_4 + 2^* \rightleftharpoons CH_3^* + H^*$	...	...	5.37E-20	16	1E-10	104.150	-9.69E+04
$H_2O + * \rightleftharpoons H_2O^*$	...	...	2.68E-20	18	0.85	2.654	7.42E+03

Table S 16. Input values of surface reactions for microkinetics simulations on Ni(100).

Ni(100)				
**Elementary reaction**	ν_{forw}	ν_{backw}	$E_{act\ forw}$	$E_{act\ backw}$
$CO_2^* + * \rightleftharpoons CO^* + O^*$	2.67E+13	3.81E+12	1.46E+04	1.06E+05
$CO^* +* \rightleftharpoons C^* + O^*$	9.03E+11	6.58E+12	1.38E+05	2.09E+05
$C^* + H^* \rightleftharpoons CH^* + *$	6.82E+12	8.48E+12	6.38E+04	3.75E+04
$CH^* + H^* \rightleftharpoons CH_2^* + *$	6.20E+12	7.46E+12	5.91E+04	-6.34E+02
$CH_2^* + H^* \rightleftharpoons CH_3^* + *$	9.59E+12	2.43E+12	1.16E+05	7.58E+04
$CO_2^* + H^* \rightleftharpoons COOH^* + *$	9.89E+13	1.75E+13	8.81E+04	6.35E+04
$COOH^* + * \rightleftharpoons COH^* + O^*$	2.91E+12	3.78E+12	1.20E+05	2.00E+05
$COH^* + H^* \rightleftharpoons HCOH^* + *$	5.77E+11	4.39E+11	1.31E+05	1.52E+04
$HCOH^* + H^* \rightleftharpoons H_2COH^*$	5.73E+12	9.29E+12	4.44E+04	2.96E+04
$H_2COH^* + H^* \rightleftharpoons H_3COH^* + *$	3.06E+12	1.72E+11	7.12E+04	6.57E+04
$CO_2^* + H^* \rightleftharpoons HCOO^* + *$	4.89E+12	9.45E+11	4.47E+04	6.31E+04
$HCOO^* + * \rightleftharpoons HCO^* + O^*$	4.83E+12	3.45E+13	1.58E+05	1.88E+05
$HCO^* + H^* \rightleftharpoons H_2CO^* + *$	7.86E+12	8.66E+12	5.84E+04	2.07E+04
$H_2CO^* + H^* \rightleftharpoons H_3CO^* + *$	1.40E+13	4.20E+11	8.21E+04	5.41E+04
$COOH^* + * \rightleftharpoons CO^* + OH^*$	3.86E+13	1.58E+13	3.00E+04	1.14E+05
$COH^* + * \rightleftharpoons CO^* + H^*$	8.25E+12	5.13E+12	8.51E+04	1.22E+05
$COH^* + * \rightleftharpoons C^* + OH^*$	3.71E+12	8.55E+12	8.77E+04	1.62E+05
$HCOH^* + * \rightleftharpoons CH^* + OH^*$	1.28E+12	4.83E+12	1.99E+04	1.84E+05
$H_2COH^* + * \rightleftharpoons CH_2^* + OH^*$	1.23E+12	3.45E+12	2.40E+04	1.43E+05
$H_3COH^* + * \rightleftharpoons CH_3^* + OH^*$	1.37E+12	1.72E+13	1.51E+05	2.35E+05
$HCO^* + * \rightleftharpoons CH^* + O^*$	6.36E+12	5.94E+12	1.62E+05	2.50E+05
$HCO^* + * \rightleftharpoons CO^* + H^*$	1.41E+13	1.45E+12	6.83E+04	1.12E+05
$H_2CO^* + * \rightleftharpoons CH_2^* + O^*$	1.51E+13	1.54E+13	1.09E+05	1.74E+05
$H_3CO^* + * \rightleftharpoons CH_3^* + O^*$	1.61E+12	1.38E+13	1.15E+05	1.68E+05
$HCO^* + H^* \rightleftharpoons HCOH^* + *$	1.44E+13	1.82E+12	1.52E+05	4.29E+04
$H_2CO^* + H^* \rightleftharpoons H_2COH^*$	2.64E+13	4.89E+12	1.37E+05	5.09E+04
$H_3CO^* + H^* \rightleftharpoons H_3COH^*$	5.36E+12	1.86E+12	9.95E+04	3.58E+04
$H^* + O^* \rightleftharpoons OH^* + *$	2.45E+12	1.25E+12	1.52E+05	1.19E+05
$OH^* + H^* \rightleftharpoons H_2O^* + *$	2.69E+13	7.12E+11	1.29E+05	7.47E+04
$OH^* + OH^* \rightleftharpoons H_2O^* + O^*$	8.42E+11	4.38E+10	7.28E+04	5.06E+04

Table S 17. Input values of adsorption and desorption reactions for microkinetics simulations on Ni(100). For A-H and Lat. see Table S 12 and Table S 13, respectively.

Ni(100)							
**Elementary reaction**	**Lat.**	**A-H**	**A (m^2)**	**m (a.u.)**	**Stick. Coëff.**	**Q_{vib}(-)**	**E_{des}(J/mol)**
$CO_2 + * \rightleftharpoons CO_2^*$	...	...	6.20E-20	44	4.00E-04	12.754	4.54E+04
$H_2 + 2^* \rightleftharpoons 2H^*$	...	...	1.24E-19	2	0.5	6.699	7.78E+04
$CH_4 + 2^* \rightleftharpoons CH_3^* + H^*$	...	...	1.24E-19	16	5.00E-09	27.545	-7.73E+04
$H_2O + * \rightleftharpoons H_2O^*$	...	...	6.20E-20	18	0.68	6.617	2.00E+04

Table S 18. Input values of surface reactions for microkinetics simulations on Ni(110).

Ni(110)				
Elementary reaction	ν_{forw}	ν_{backw}	$E_{act\ forw}$	$E_{act\ backw}$
$CO_2^* + * \rightleftharpoons CO^* + O^*$	1.02E+13	5.09E+12	3.94E+04	5.71E+04
$CO^* +* \rightleftharpoons C^* + O^*$	5.81E+11	9.48E+12	1.50E+05	9.95E+04
$C^* + H^* \rightleftharpoons CH^* + *$	8.52E+12	6.55E+12	7.01E+04	7.60E+04
$CH^* + H^* \rightleftharpoons CH_2^* + *$	2.15E+13	4.66E+12	5.80E+04	2.70E+04
$CH_2^* + H^* \rightleftharpoons CH_3^* + *$	4.09E+12	4.75E+12	2.62E+04	5.50E+04
$CO_2^* + H^* \rightleftharpoons COOH^* + *$	1.94E+13	1.87E+13	8.81E+04	6.59E+04
$COOH^* + * \rightleftharpoons COH^* + O^*$	4.40E+13	3.84E+13	2.23E+05	1.71E+05
$COH^* + H^* \rightleftharpoons HCOH^* + *$	7.31E+12	1.15E+13	8.13E+04	7.89E+04
$HCOH^* + H^* \rightleftharpoons H_2COH^*$	9.78E+12	7.97E+12	3.29E+04	1.05E+04
$H_2COH^* + H^* \rightleftharpoons H_3COH^* + *$	4.76E+12	2.74E+11	6.05E+04	6.78E+04
$CO_2^* + H^* \rightleftharpoons HCOO^* + *$	4.48E+12	4.05E+12	2.51E+04	5.25E+04
$HCOO^* + * \rightleftharpoons HCO^* + O^*$	1.90E+12	7.24E+12	1.34E+05	6.93E+04
$HCO^* + H^* \rightleftharpoons H_2CO^* + *$	1.10E+13	1.43E+13	3.52E+04	2.92E+04
$H_2CO^* + H^* \rightleftharpoons H_3CO^* + *$	2.18E+13	3.08E+12	1.00E+05	9.91E+04
$COOH^* + * \rightleftharpoons CO^* + OH^*$	1.23E+13	2.48E+12	3.37E+04	1.12E+05
$COH^* + * \rightleftharpoons CO^* + H^*$	3.04E+12	1.81E+12	3.21E+04	1.24E+05
$COH^* + * \rightleftharpoons C^* + OH^*$	3.35E+12	1.27E+13	6.13E+04	1.41E+05
$HCOH^* + * \rightleftharpoons CH^* + OH^*$	8.04E+12	1.48E+13	6.62E+04	1.55E+05
$H_2COH^* + * \rightleftharpoons CH_2^* + OH^*$	1.03E+13	5.05E+12	6.81E+04	1.48E+05
$H_3COH^* + * \rightleftharpoons CH_3^* + OH^*$	2.21E+13	2.19E+14	1.56E+05	2.57E+05
$HCO^* + * \rightleftharpoons CH^* + O^*$	8.31E+12	1.51E+13	9.84E+04	1.09E+05
$HCO^* + * \rightleftharpoons CO^* + H^*$	5.87E+13	8.51E+12	3.14E+04	8.69E+04
$H_2CO^* + * \rightleftharpoons CH_2^* + O^*$	6.71E+12	2.03E+12	8.23E+04	6.84E+04
$H_3CO^* + * \rightleftharpoons CH_3^* + O^*$	2.65E+13	6.58E+13	1.82E+05	1.98E+05
$HCO^* + H^* \rightleftharpoons HCOH^* + *$	1.63E+13	6.28E+12	6.38E+04	2.47E+04
$H_2CO^* + H^* \rightleftharpoons H_2COH^*$	2.48E+13	5.98E+12	1.42E+05	8.67E+04
$H_3CO^* + H^* \rightleftharpoons H_3COH^*$	8.17E+12	7.98E+11	9.66E+04	4.99E+04
$H^* + O^* \rightleftharpoons OH^* + *$	1.15E+13	4.47E+12	9.82E+04	1.37E+05
$OH^* + H^* \rightleftharpoons H_2O^* + *$	7.28E+12	4.95E+11	1.41E+05	1.00E+05
$OH^* + OH^* \rightleftharpoons H_2O^* + O^*$	1.18E+13	2.07E+12	1.10E+05	3.11E+04

Table S 19. Input values of adsorption and desorption reactions for microkinetics simulations on Ni(110). For A-H and Lat. see Table S 12 and Table S 13, respectively.

Ni(110)							
Elementary reaction	Lat.	A-H	A (m^2)	m (a.u.)	Stick. Coëff.	Q_{vib}(-)	E_{des}(J/mol)
$CO_2 + * \rightleftharpoons CO_2^*$	...	...	2.19E-20	44	0.1	11.768	5.87E+04
$H_2 + 2^* \rightleftharpoons 2H^*$	...	...	4.38E-20	2	0.96	4.362	7.94E+04
$CH_4 + 2^* \rightleftharpoons CH_3^* + H^*$	...	...	4.38E-20	16	1.00E-08	20.944	-7.00E+04
$H_2O + * \rightleftharpoons H_2O^*$	...	...	2.19E-20	18	1	5.652	3.23E+04

Table S 20. Input values of surface reactions for microkinetics simulations on Ni(211).

Ni(211)				
Elementary reaction	ν forw	ν backw	E act forw	E act backw
CO_2* + * ⇌ CO* + O*	3.43E+10	7.52E+12	9.05E+04	1.58E+05
CO* +* ⇌ C* + O*	1.04E+12	7.52E+12	1.58E+05	1.68E+05
C* + H* ⇌ CH* + *	1.07E+13	7.52E+12	6.15E+04	3.77E+04
CH* + H* ⇌ CH_2* + *	1.94E+13	7.52E+12	7.43E+04	2.57E+04
CH_2* + H* ⇌ CH_3* + *	5.12E+13	7.52E+12	4.36E+04	5.87E+04
CO_2* + H* ⇌ COOH* + *	2.00E+12	7.52E+12	9.00E+04	6.74E+04
COOH* + * ⇌ COH* + O*	3.82E+12	7.52E+12	1.65E+05	1.75E+05
COH* + H* ⇌ HCOH* + *	1.62E+13	7.52E+12	9.71E+04	4.30E+04
HCOH* + H* ⇌ H_2COH*	1.04E+13	7.52E+12	3.27E+04	2.00E+04
H_2COH* + H* ⇌ H_3COH* + *	3.61E+13	7.52E+12	7.90E+04	5.88E+04
CO_2* + H* ⇌ HCOO* + *	4.17E+12	7.52E+12	8.72E+04	1.18E+05
HCOO* + * ⇌ HCO* + O*	7.76E+12	7.52E+12	1.67E+05	1.33E+05
HCO* + H* ⇌ H_2CO* + *	1.41E+13	7.52E+12	8.73E+04	2.92E+04
H_2CO* + H* ⇌ H_3CO* + *	1.11E+13	7.52E+12	7.82E+04	1.06E+05
COOH* + * ⇌ CO* + OH*	9.52E+13	7.52E+12	3.68E+04	1.35E+05
COH* + * ⇌ CO* + H*	1.29E+13	7.52E+12	3.13E+04	1.11E+05
COH* + * ⇌ C* + OH*	3.49E+12	7.52E+12	9.01E+04	1.88E+05
HCOH* + * ⇌ CH* + OH*	5.90E+12	7.52E+12	1.12E+05	2.40E+05
H_2COH* + * ⇌ CH_2* + OH*	1.80E+12	7.52E+12	1.36E+05	2.28E+05
H_3COH* + * ⇌ CH_3* + OH*	1.19E+11	7.52E+12	1.47E+05	2.74E+05
HCO* + * ⇌ CH* + O*	1.55E+13	7.52E+12	1.67E+05	2.25E+05
HCO* + * ⇌ CO* + H*	1.57E+13	7.52E+12	1.84E+03	7.31E+04
H_2CO* + * ⇌ CH_2* + O*	1.60E+13	7.52E+12	1.20E+05	1.88E+05
H_3CO* + * ⇌ CH_3* + O*	8.67E+12	7.52E+12	1.98E+05	2.53E+05
HCO* + H* ⇌ HCOH* + *	2.55E+13	7.52E+12	1.79E+05	1.17E+05
H_2CO* + H* ⇌ H_2COH*	4.84E+13	7.52E+12	7.41E+04	5.69E+04
H_3CO* + H* ⇌ H_3COH*	1.63E+14	7.52E+12	1.04E+05	3.87E+04
H* + O* ⇌ OH* + *	9.38E+12	7.52E+12	8.80E+04	9.57E+04
OH* + H* ⇌ H_2O* + *	4.27E+13	7.52E+12	1.35E+05	7.15E+04
OH* + OH* ⇌ H_2O* + O*	8.33E+12	7.52E+12	1.06E+05	3.51E+04

Table S 21. Input values of adsorption and desorption reactions for microkinetics simulations on Ni(211). For A-H and Lat. see Table S 12 and Table S 13, respectively.

Ni(211)							
Elementary reaction	Lat.	A-H	A (m^2)	m (a.u.)	Stick. Coëff.	Q_{vib}(-)	E_{des}(J/mol)
CO_2 + * ⇌ CO_2*	...	...	6.10E-20	44	9.33E-02	12.526	4.15E+04
H_2 + 2* ⇌ 2H*	...	...	5.24E-20	2	0.7	2.648	6.37E+04
CH_4 + 2* ⇌ CH_3* + H*	...	...	5.24E-20	16	2.80E-07	15.129	-1.13E+05
H_2O + * ⇌ H_2O*	...	...	6.10E-20	18	1	5.125	3.72E+04

Output

Degree of Rate Control

Heatmaps of the Degree of Rate Control (DRC) coefficients of each elementary reaction step from 500-800 K for each nickel facet are given in Figure S 44. Both CO^{*} and H^{*} were destabilized with 40 and 20 kJ/mol, respectively. Note a different range in the coefficients for Ni(100) compared to the other facets.

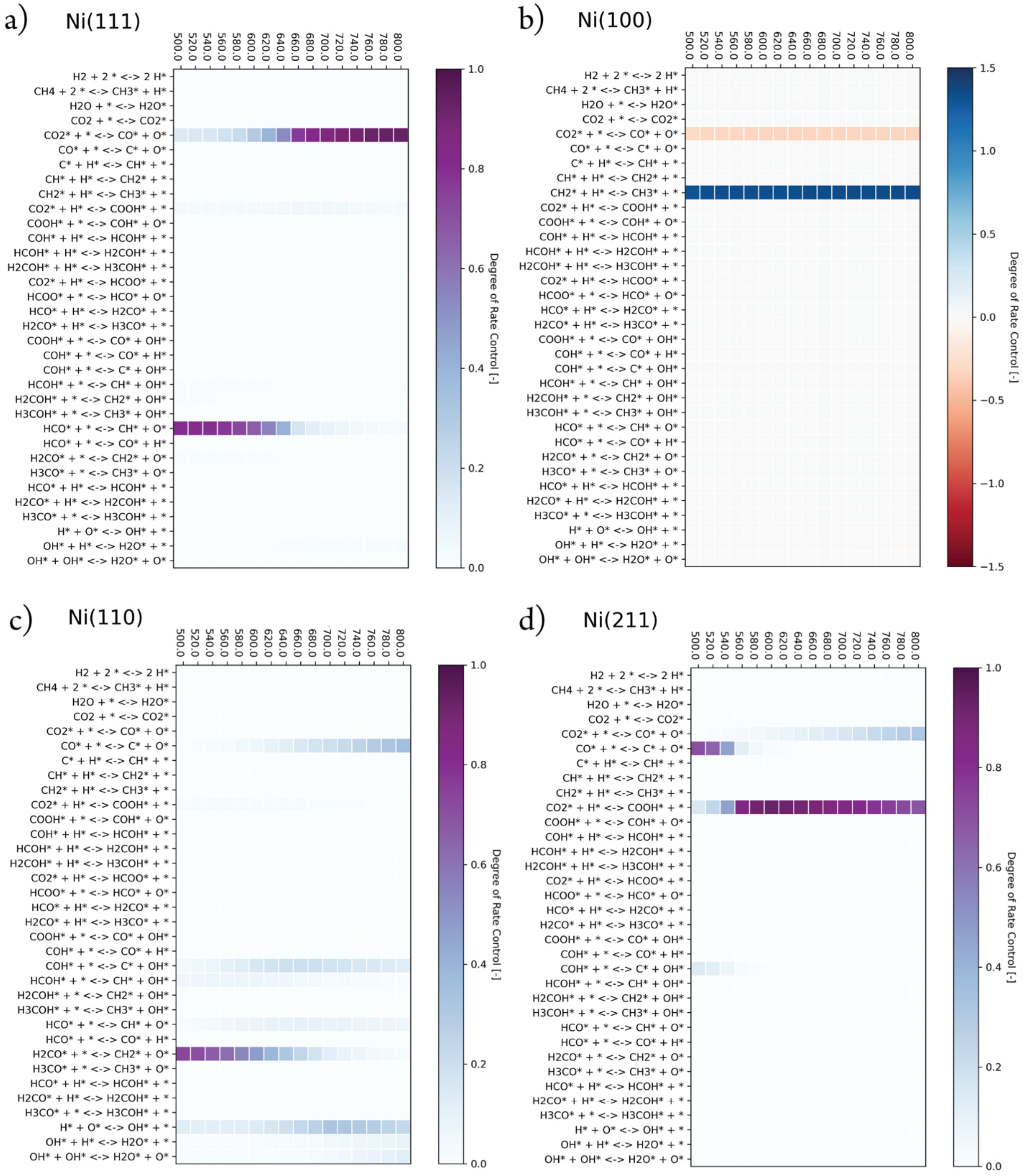

Figure S 44. Degree of Rate Control (DRC) coefficients of each elementary reaction step as a function of temperature for a) Ni(111), b) Ni(100), c) Ni(110) and d) Ni(211).

Rate methane formation

For the boundary conditions of the MKM simulations we have used the stoichiometric ratio of 1:4 for the partial pressure of CO_2 and H_2 and the total pressure was set at 1 bar. The obtained methane production rates as a function of temperature are tabulated below (Table S 22).

Table S 22. Methane production rate from 500 - 800 K for four nickel facets.

Rate methane formation (s^{-1})				
**Temperature (K)**	**Ni(111)**	**Ni(100)**	**Ni(110)**	**Ni(211)**
500	6.51E-10	1.23E-09	4.66E-04	4.01E-06
520	3.89E-09	3.77E-09	1.36E-03	1.64E-05
540	1.97E-08	1.05E-08	3.62E-03	4.96E-05
560	8.54E-08	2.66E-08	8.91E-03	8.64E-05
580	3.18E-07	6.27E-08	2.03E-02	1.10E-04
600	1.02E-06	1.38E-07	4.32E-02	1.27E-04
620	2.78E-06	2.87E-07	8.58E-02	1.40E-04
640	6.46E-06	5.64E-07	1.59E-01	1.51E-04
660	1.20E-05	1.06E-06	2.74E-01	1.59E-04
680	1.94E-05	1.90E-06	4.37E-01	1.65E-04
700	2.94E-05	3.29E-06	6.29E-01	1.69E-04
720	4.23E-05	5.49E-06	8.43E-01	1.73E-04
740	5.83E-05	8.87E-06	1.08E+00	1.76E-04
760	7.76E-05	1.39E-05	1.34E+00	1.78E-04
780	1.00E-04	2.13E-05	1.61E+00	1.79E-04
800	1.27E-04	3.17E-05	1.88E+00	1.80E-04

Flux diagram at 580 K for Ni(111)

B. Kreitz et al[7]. developed a microkinetic model for the hydrogenation of CO_2 on Ni(111) at 573 K by means of automatic mechanism generation combined with a global sensitivity analysis. They found that 75 % of CO^* formation occurs via direct CO_2^* dissociation and the other 25 % is formed via various elementary reaction steps in the carboxyl pathway. They also found that the only significant source of CH^* originates from the dissociation of HCO^*.

In order to compare these findings with our microkinetic model, we constructed a flux diagram of Ni(111) at 580 K, which is depicted below (Figure S 45). We see that 88 % of CO^* formation occurs via direct CO_2^* dissociation and the other 12 % is formed via $COOH^*$ intermediate, which is part of the carboxyl pathway. Just like we saw at 640 K, also at 580 K we see that for Ni(111) there is only a significant formation of CH^* via the HCO^* intermediate. The results from our microkinetic model are in very close agreement with proposed mechanism by B. Kreitz et al.[7].

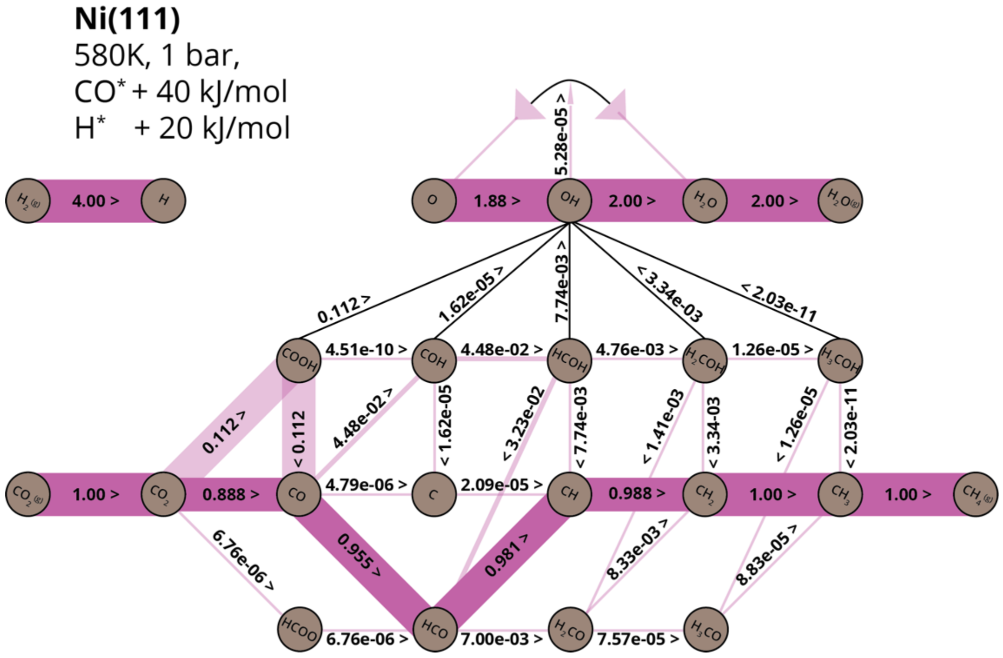

Figure S 45. Flux diagram of Ni(111) at 580 K and 1 bar. Bars of elementary reaction steps with significant - but not the largest flux – are transparent. The thickness of the bar that connects the nodes of the reaction network scales with the size of the flux between these nodes. The direction of the flux is indicated with ‘<’ or ‘>’.

[7] Kreitz, B.; Sargsyan, K.; Blöndal, K.; Mazeau, E. J.; West, R. H.; Wehinger, G. D.; Turek, T.; Goldsmith, C. F. Quantifying the Impact of Parametric Uncertainty on Automatic Mechanism Generation for CO 2 Hydrogenation on Ni(111) . *JACS Au* **2021**, No. 111. https://doi.org/10.1021/jacsau.1c00276

I Sensitivity Analysis on the Correction of CO* Overbinding

In this part we examine the effect of the correction for CO* overbinding. For this we performed several MKM simulations were CO* was increasingly destabilized with respect to its most stable adsorption energy, by adding 0, 15, 30 and 40 kJ/mol to its adsorption energy. Other settings were kept the same compared to the simulations as presented in the manuscript. Thus, the most stable adsorption energy was used for other reaction intermediates and H* was destabilized with 20 kJ/mol. The simulations were performed with an initial CO_2:H_2 mixture of 1:4, a total pressure of 1 bar and temperatures between 500 - 800 K. The same lateral interaction potential was included as presented in Table S 13.
Below, the results of apparent activation energy, reaction rate, surface coverage, reaction order in H_2 and CO_2, degree of rate control and reaction fluxes are presented.

Reaction rate
In Figure S 46, the reaction rates are plotted on a logarithmic scale as a function of temperature. Clearly, an increase in CO* destabilization results in a higher reaction rate for Ni(111), Ni(110) and Ni(211). For Ni(211) an upper limit in the reaction rate can be observed by the plateau around 640 K. This limitation is the result of the absence of carbonaceous species at higher temperatures. The destabilization of CO* does not have an effect on the reaction rate for Ni(100). This is likely to be caused by the fact that Ni(100) is suffering from a high surface coverage of C*. Destabilizing the CO* intermediate increases the formation of C*, which on its turn blocks the surface for the formation of methane.

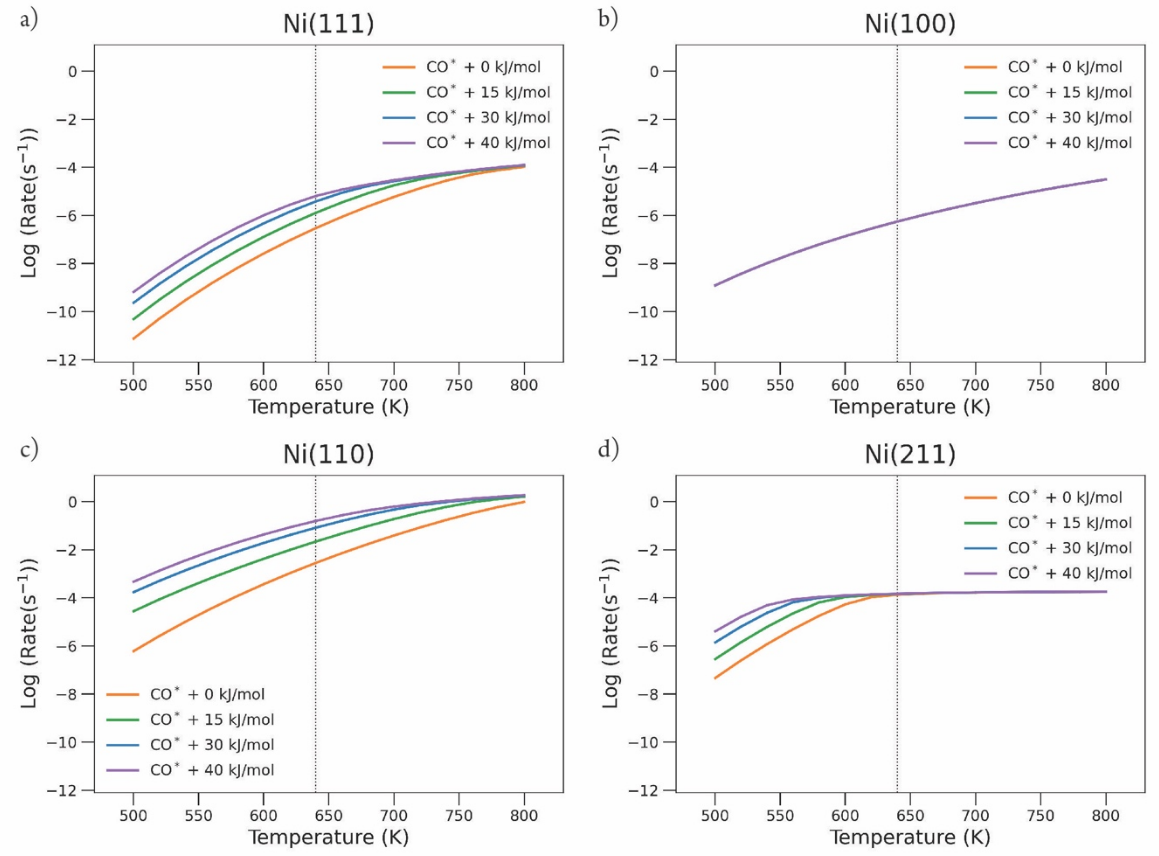

Figure S 46. Reaction rate on a logarithmic scale as a function of temperature with an increasing destabilization of CO* from 0 to 40 kJ/mol. a) Ni(111), b) Ni(100), c) Ni(110) and d) Ni(211).

Apparent activation energy

The apparent activation energy as a function of temperature is plotted in Figure S 47. For Ni(111), Ni(110) and Ni(211) a decrease in the stability of CO* results in a shift of the inflection towards lower reaction temperatures. These temperature profiles indicate that the transition from an inactive to an active catalyst occur at lower temperature. The apparent activation energy at 640 K for the most active facet, Ni(110), is 163.1, 133.6, 115.5 and 98.8 kJ/mol for CO* destabilization of 0, 15, 30 and 40 kJ/mol respectively. Reported apparent activation energies for CO_2 methanation over nickel catalysts supported on various metal oxides range from 77-92kJ/mol[8,9,10,11,12]. The calculated apparent activation energy of 98.8 kJ/mol for Ni(110) at 640 K gives a satisfactory agreement with literature.

The destabilization of CO* does not have an effect on the apparent activation energy on Ni(100) in the studied temperature regime. This is likely to be caused by the fact that Ni(100) is suffering from a high surface coverage of C*, even when CO* is not destabilized.

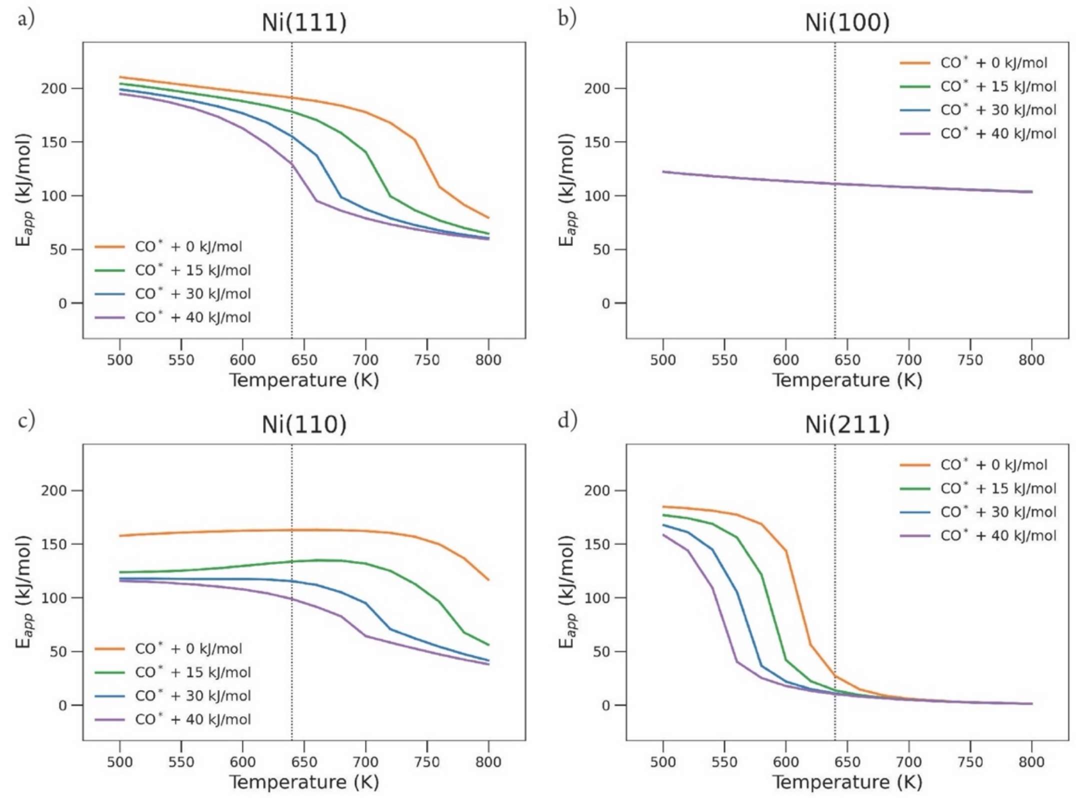

Figure S 47. Apparent activation energy as a function of temperature with an increasing destabilization of CO* from 0 to 40 kJ/mol. a) Ni(111), b) Ni(100), c) Ni(110) and d) Ni(211).

---

[8] Wang, W.; Wang, S.; Ma, X.; Gong, J. Recent Advances in Catalytic Hydrogenation of Carbon Dioxide. *Chem. Soc. Rev.* **2011**, *40* (7), 3703–3727. https://doi.org/10.1039/c1cs15008a.

[9] Vogt, C.; Monai, M.; Sterk, E. B.; Palle, J.; Melcherts, A. E. M.; Zijlstra, B.; Groeneveld, E.; Berben, P. H.; Boereboom, J. M.; Hensen, E. J. M.; Meirer, F.; Filot, I. A. W.; Weckhuysen, B. M. Understanding Carbon Dioxide Activation and Carbon–Carbon Coupling over Nickel. *Nat. Commun.* **2019**, *10* (1), 1–10. https://doi.org/10.1038/s41467-019-12858-3.

[10] Van Herwijnen, T.; Van Doesburg, H.; De Jong, W. A. Kinetics of the Methanation of CO and CO2 on a Nickel Catalyst. *J. Catal.* **1973**, *28* (3), 391–402. https://doi.org/10.1016/0021-9517(73)90132-2.

[11] Weatherbee, G. D.; Bartholomew, C. H. Hydrogenation of CO2 on Group VIII Metals. I. Specific Activity of Ni SiO2. *J. Catal.* **1981**, *68* (1), 67–76. https://doi.org/10.1016/0021-9517(81)90040-3 *J. Catal.*.

[12] Peebles, D. E.; Goodman, D. W.; White, J. M. Methanation of Carbon Dioxide on Ni(100) and the Effects of Surface Modifiers. *J. Phys. Chem.* **1983**, *87* (22), 4378–4387. https://doi.org/10.1021/j100245a014.

I. Sensitivity Analysis on the Correction of CO^* Overbinding

Surface coverage

The surface coverages as a function of temperature are plotted in Figure S 48. The color represents the magnitude in which CO^* was destabilized, while a marker is indicative for the type of reaction intermediate.

A similar trend in surface coverage can be observed for Ni(111), Ni(110) and Ni(211). With an increase in CO^* destabilization the surface coverage of CO^* decreases, while the relative amount of H^* and vacant sites increases. The destabilization of CO^* does not have an effect on the surface coverage for Ni(100), since this facet is highly covered with C^* over the whole temperature range.

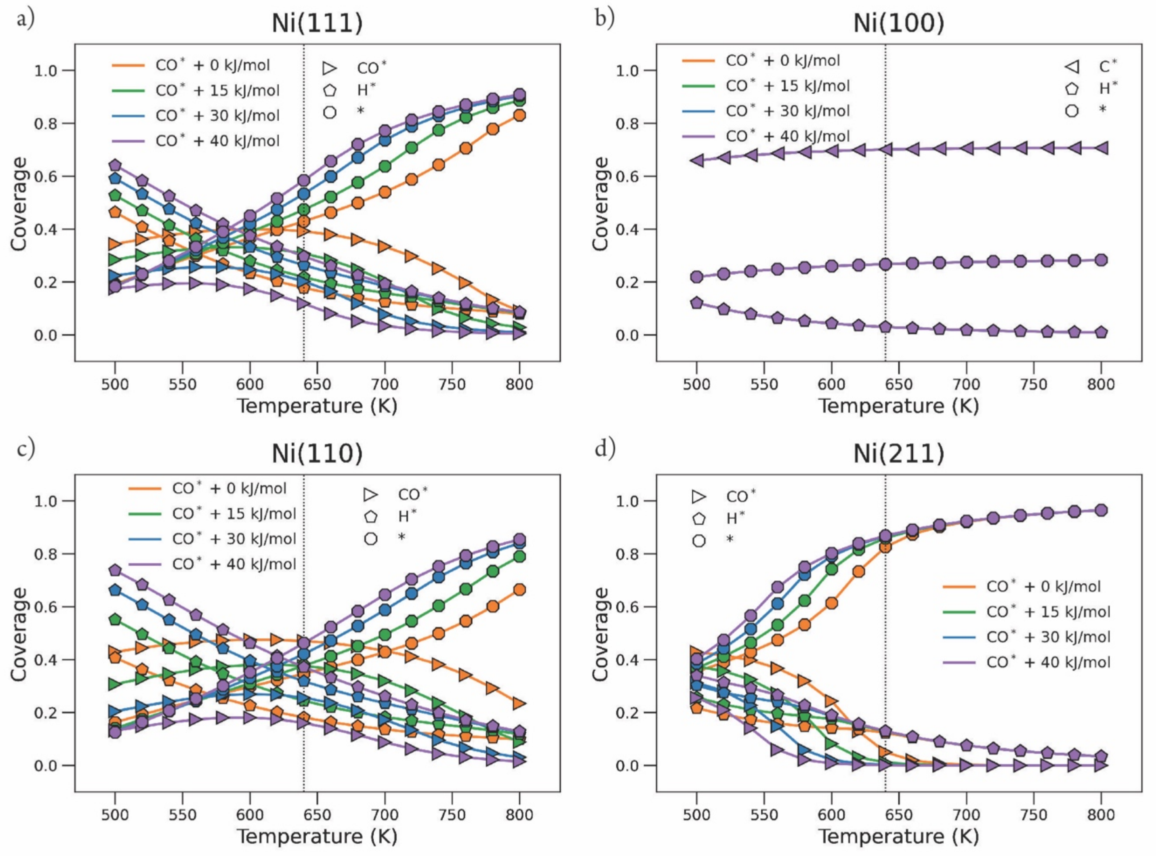

Figure S 48. Surface coverage as a function of temperature with an increasing destabilization of CO^* from 0 to 40 kJ/mol. The color represents the magnitude in which CO^* was destabilized, while the marker is indicative for the type of reaction intermediate. a) Ni(111), b) Ni(100), c) Ni(110) and d) Ni(211).

Reaction orders

On the next page (Figure S 49) the reaction orders in CO_2 and H_2 are plotted as a function of temperature. Note: the scale of the y-axis differs among different facets. In general, the reaction order in CO_2 increases with an increase in CO^* destabilization. For the order in H_2 a decrease is observed with an increase in CO^* destabilization. Two special cases can be assigned: firstly, for Ni(110) the reaction order in H_2 is the smallest when CO^* is not destabilized. Secondly, in the case of Ni(211), at low temperatures the order in H_2 decreases with an increase in CO^* destabilization, while the opposite is true for temperatures higher then 580 K. For Ni(100) a change in the stability of CO^* does not influence the reaction orders.

I. Sensitivity Analysis on the Correction of CO^* Overbinding

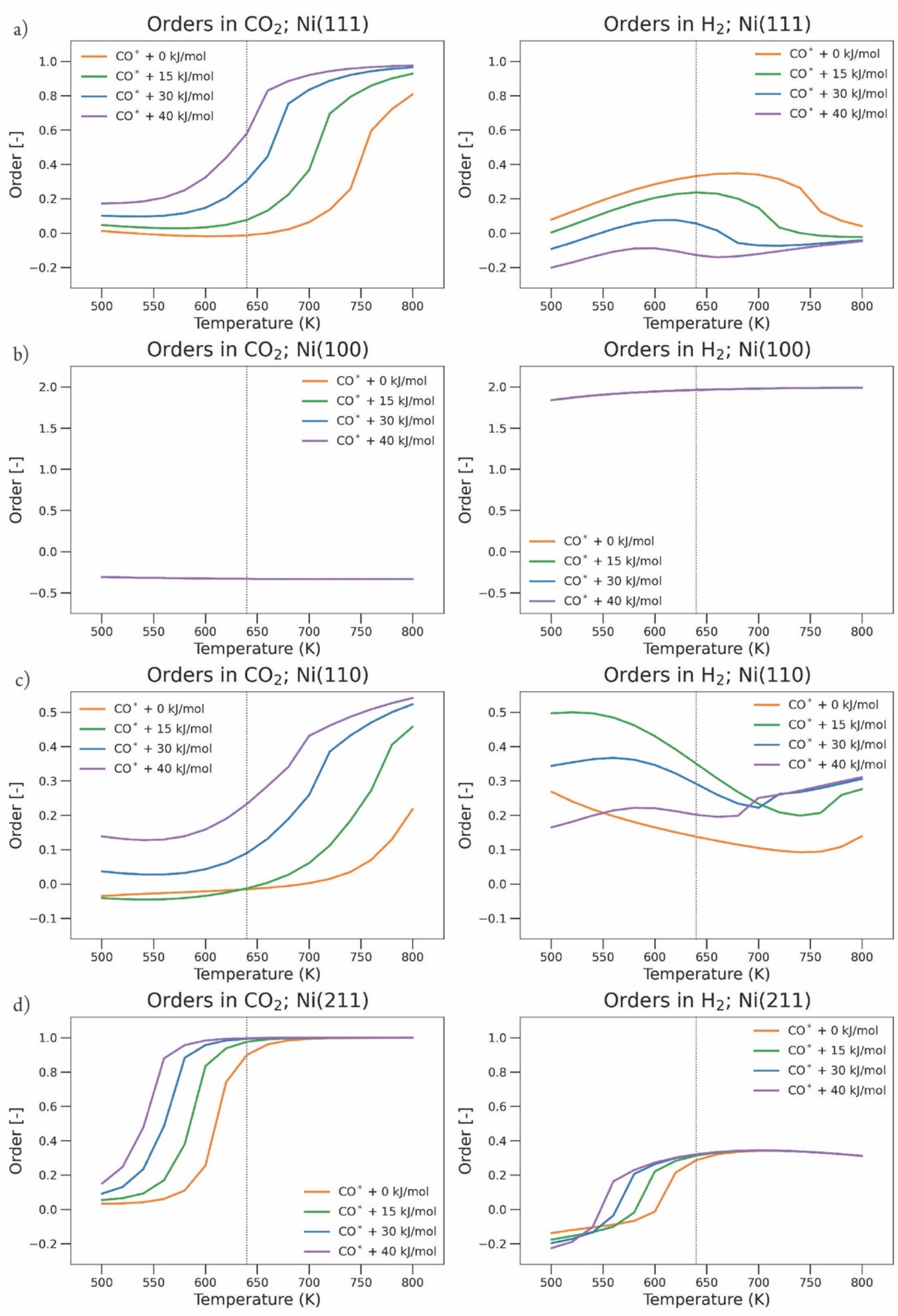

Figure S 49. The reaction orders in CO_2 (left) and H_2 (right) as a function of temperature with an increasing destabilization of CO^* from 0 to 40 kJ/mol. Note: the scale of the y-axis differs among different facets. a) Ni(111), b) Ni(100), c) Ni(110) and d) Ni(211).

I. Sensitivity Analysis on the Correction of CO^* Overbinding

Degree of rate control
Below, as a function of temperature the coefficients of the degree of rate control (DRC) analysis are presented in heatmaps. Note: the scale for Ni(100) runs from -1.5 to 1.5 (Figure S 52), while for Ni(111) (Figure S 50), Ni(110) (Figure S 53) and Ni(211) (Figure S 51) the scale runs from 0 to 1.

CO^* destabilization does not have an effect on the DRC for Ni(100).
For the other facets, the trend in the DRC-coefficients of rate controlling elementary reaction steps shift to lower or higher temperatures. For example, in the case of Ni(111) the dissociation of CO_2^* towards CO^* and O^* becomes increasingly rate controlling at lower temperatures with a destabilization in CO^*, while the dissociation of HCO^* towards CH^* and O^* becomes less rate controlling at higher temperatures. These effects are a direct consequence of the corresponding decrease in CO^* coverage on Ni(111), see p.67. With a decrease in CO^* coverage, the first C-O bond dissociation becomes more controlling compared to the dissociation of the second C-O bond.
This can also be observed for Ni(211), where the activation of CO_2^* towards $COOH^*$ becomes more rate controlling at lower temperatures and the direct CO^* dissociation becomes less rate controlling at higher temperatures. The DRC-coefficient of the direct CO_2^* dissociation towards CO^* and O^* remain roughly constant. This already indicates that the reaction flux of hydrogen assisted CO_2^* dissociation trough $COOH^*$ is higher compared to the direct CO_2^* dissociation.
More subtle changes are observed in the case of Ni(110). Direct CO^* dissociation becomes less rate controlling when CO^* is destabilized. When CO^* is destabilized with +15, +30 and +40 kJ/mol the trend of the DRC-coefficients as a function of temperature remains roughly the same.

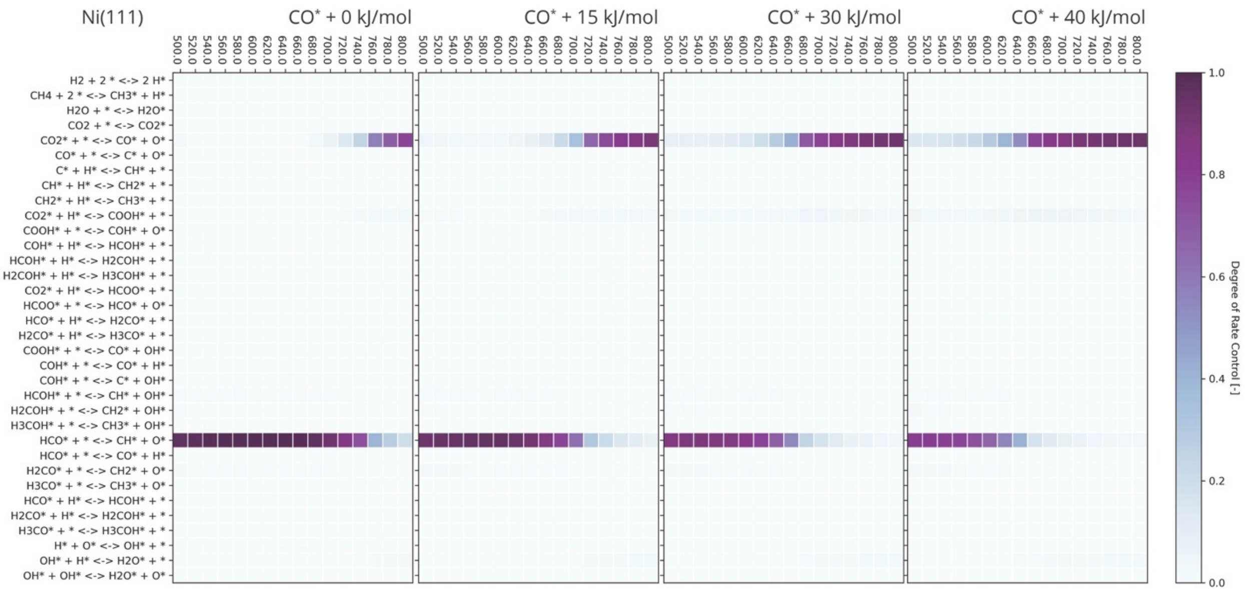

Figure S 50. Degree of Rate Control (DRC) coefficients of each elementary reaction step for Ni(111) as a function of temperature with an increasing destabilization of CO^* from 0 to 40 kJ/mol.

I. Sensitivity Analysis on the Correction of CO^* Overbinding

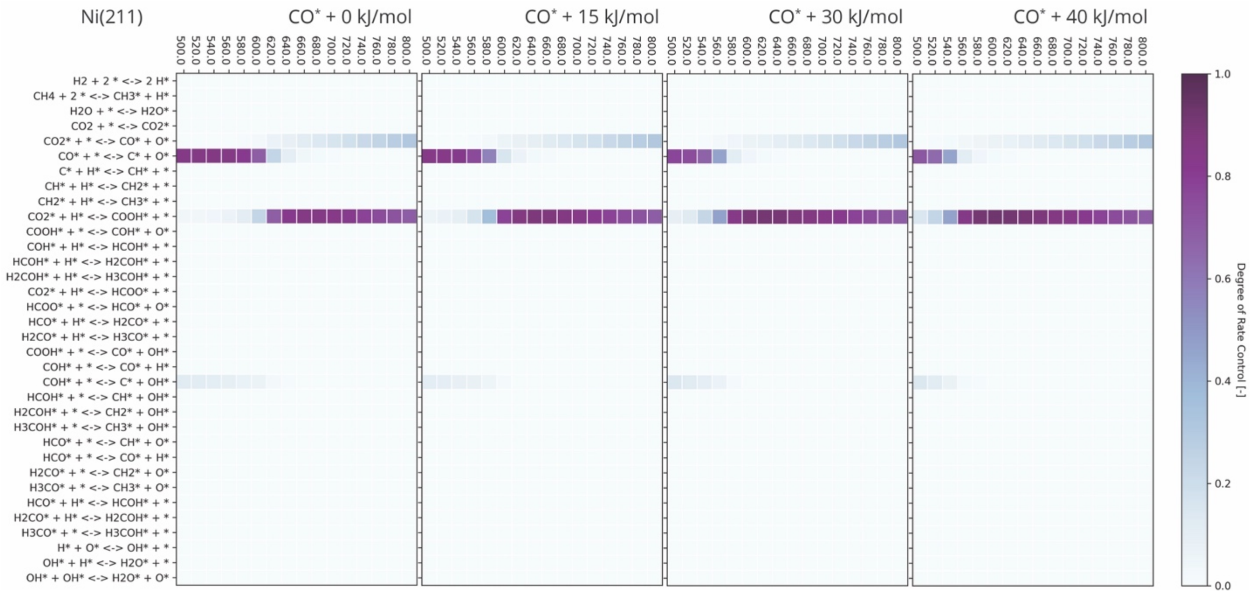

Figure S 51. Degree of Rate Control (DRC) coefficients of each elementary reaction step for Ni(211) as a function of temperature with an increasing destabilization of CO^* from 0 to 40 kJ/mol.

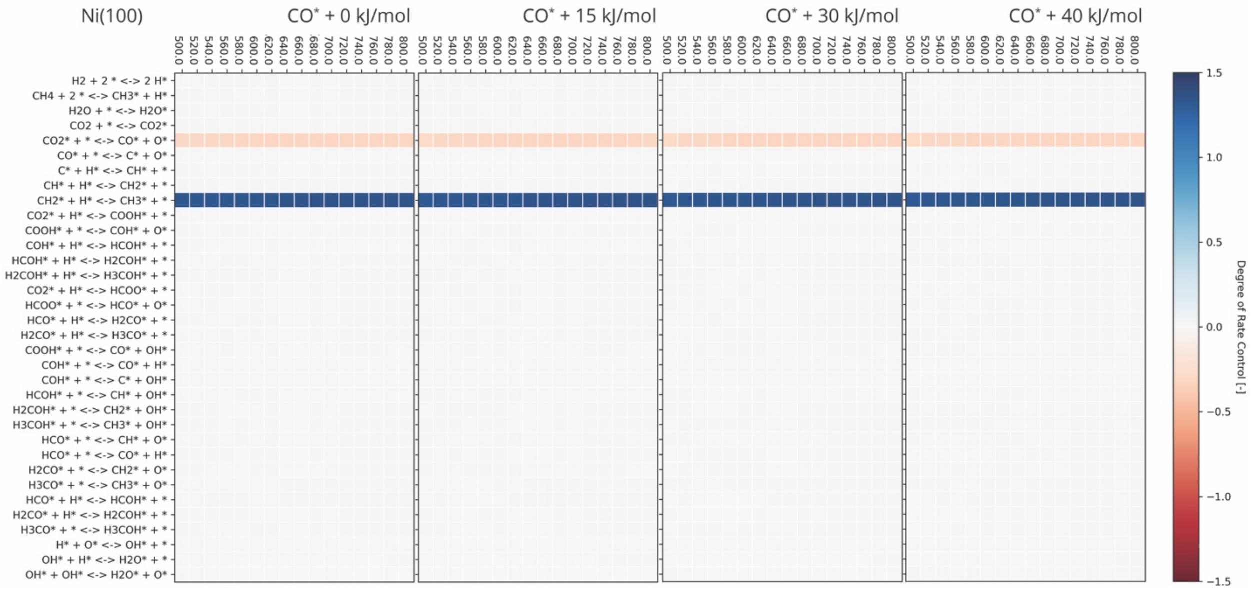

Figure S 52. Degree of Rate Control (DRC) coefficients of each elementary reaction step for Ni(100) as a function of temperature with an increasing destabilization of CO^* from 0 to 40 kJ/mol.

I. Sensitivity Analysis on the Correction of CO* Overbinding

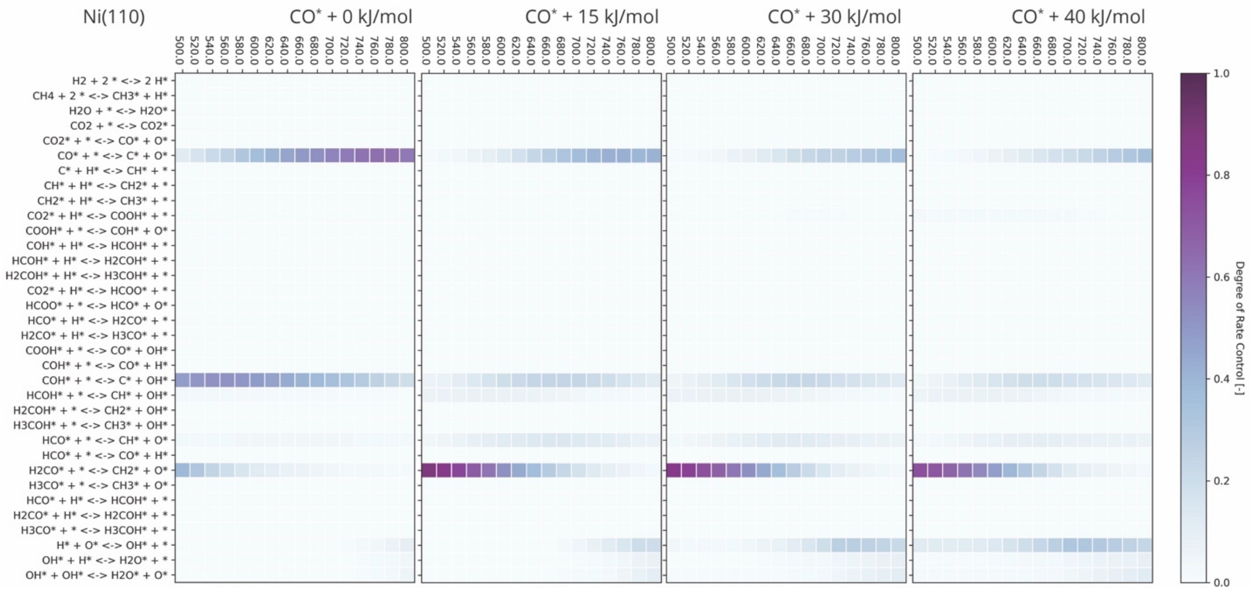

Figure S 53. Degree of Rate Control (DRC) coefficients of each elementary reaction step for Ni(110) as a function of temperature with an increasing destabilization of CO* from 0 to 40 kJ/mol.

Flux diagrams

The flux diagrams corresponding to 640 K are depicted in Figure S 54 to Figure S 57.

For Ni(111), Ni(100) and Ni(211) the significant flux remain the same with a change in CO* stability. Comparing the diagrams of Ni(111) only small differences can be seen in the magnitude of flux between nodes. The flux diagrams of Ni(100) are identical where CO* is destabilized with 30 and 40 kJ/mol. Comparing the first two diagrams of Ni(100), only small differences can be seen in the magnitude of flux between nodes.

For Ni(211) the flux diagrams where CO* is destabilized with 0, 30 or 40 kJ/mol are identical. Only the destabilization of CO* with 15 kJ/mol results in a different flux diagram, with the most prominent difference in the flux between the nodes of CO – C and O – OH.

The most apparent differences can be observed for Ni(110). When CO* is not destabilized, the main flux goes via the direct carbide mechanism on Ni(110). With an increase in the destabilization of CO*, the main flux shifts form direct CO* dissociation towards H-assisted CO* dissociation via H_2CO^*. When CO* is destabilized with 30 and 40 kJ/mol, CO_2^* dissociation via COOH* becomes significant. However, the flux for direct CO_2^* dissociation remain larger.

I. Sensitivity Analysis on the Correction of CO^* Overbinding

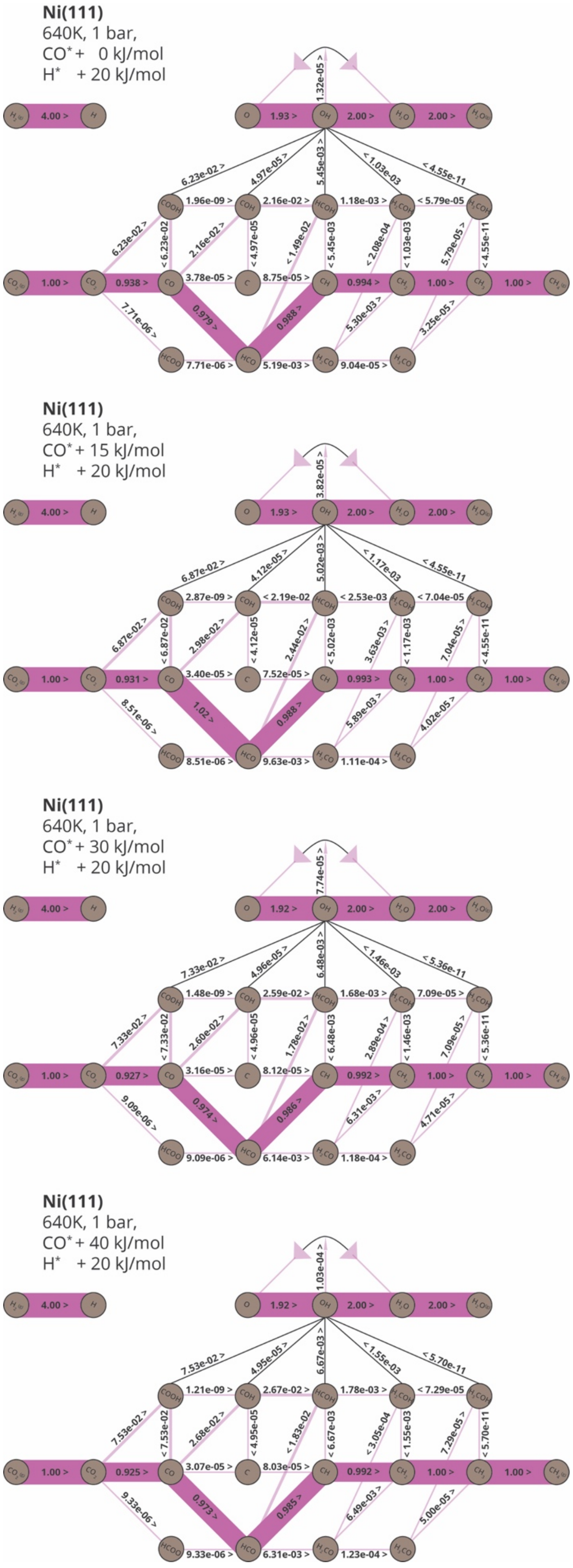

Figure S 55. Flux diagrams at 640 K for Ni(111) with an increasing destabilization of CO^* from 0 to 40 kJ/mol.

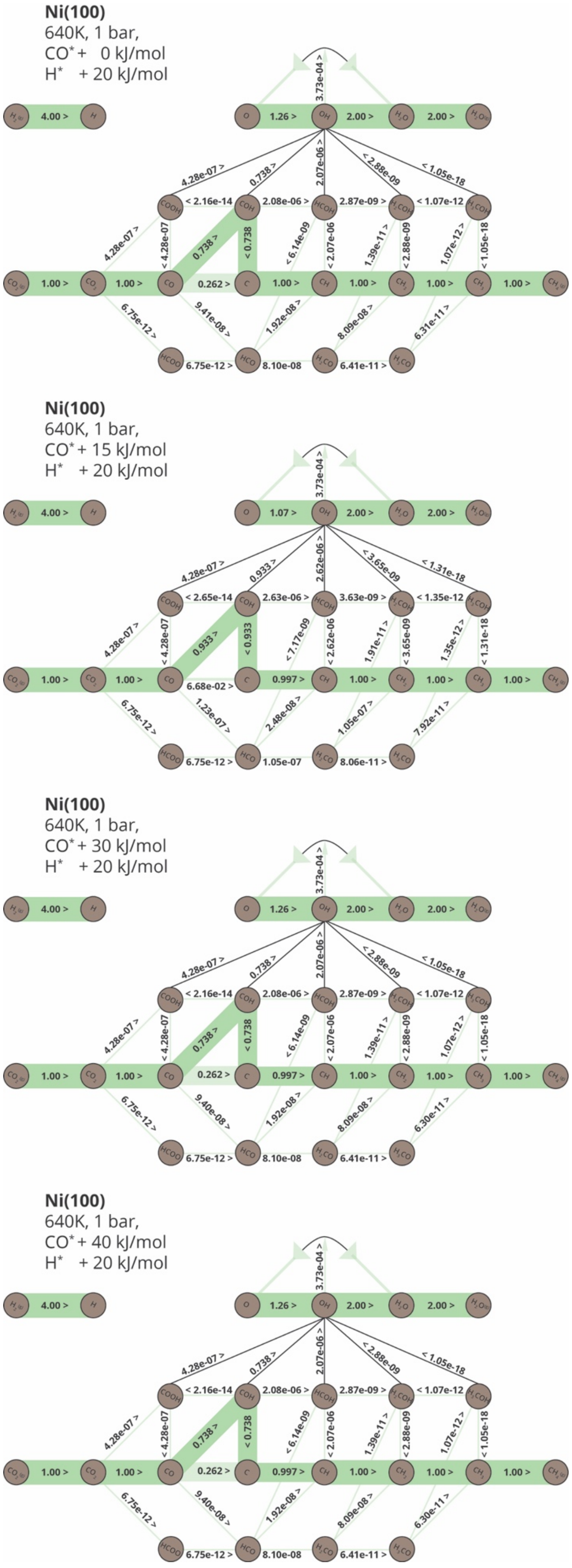

Figure S 54. Flux diagrams at 640 K for Ni(100) with an increasing destabilization of CO^* from 0 to 40 kJ/mol.

I. Sensitivity Analysis on the Correction of CO^* Overbinding

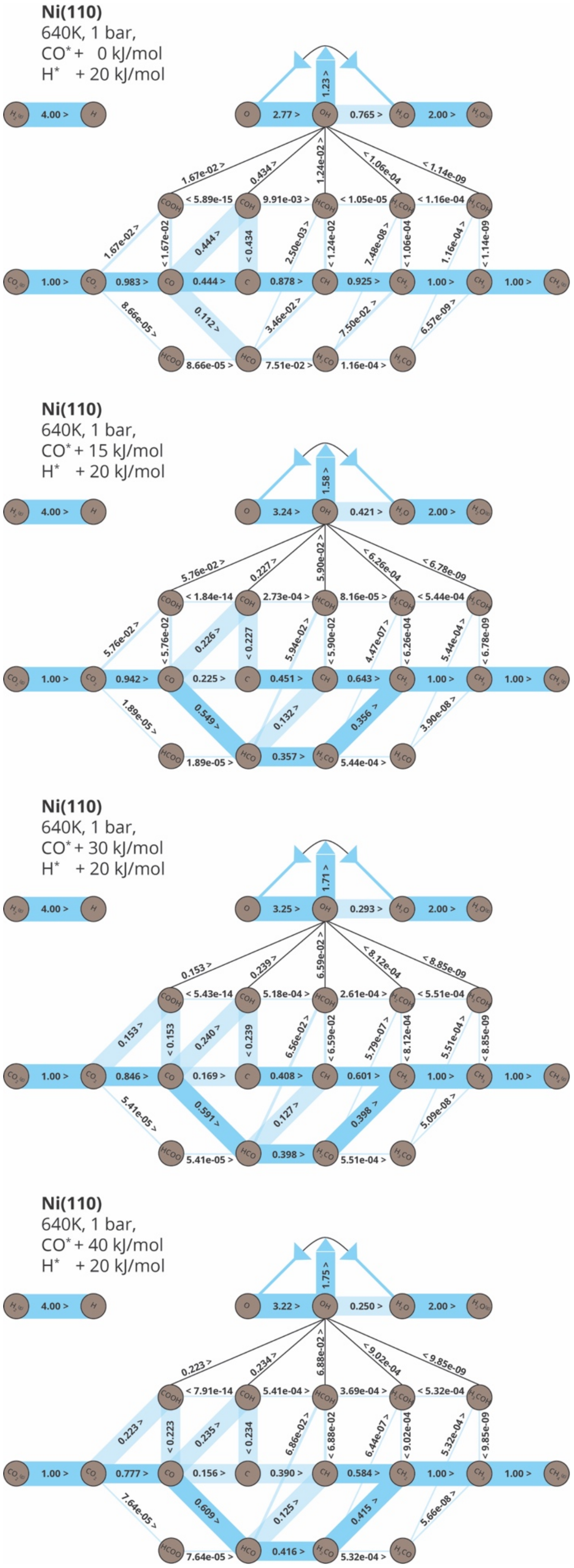

Figure S 57. Flux diagrams at 640 K for Ni(110) with an increasing destabilization of CO^* from 0 to 40 kJ/mol.

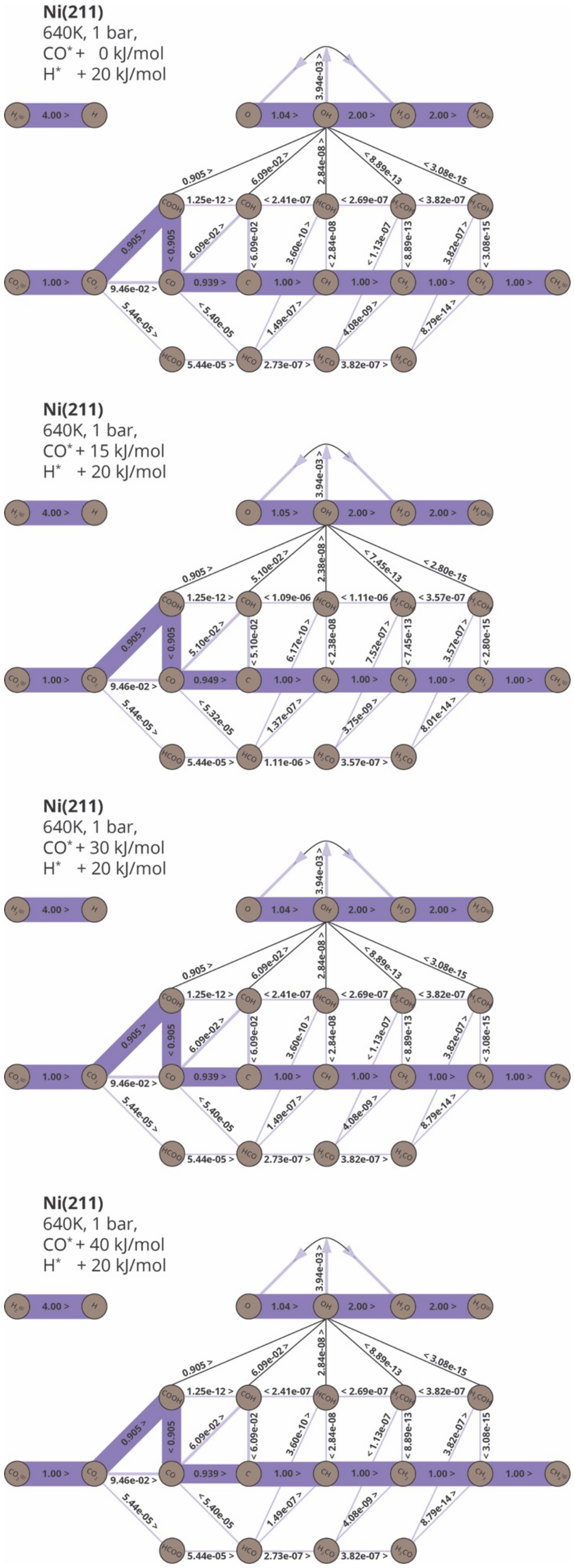

Figure S 56. Flux diagrams at 640 K for Ni(211) with an increasing destabilization of CO^* from 0 to 40 kJ/mol.

J Sensitivity Analysis on the Lateral Interaction Potential

For the inclusion of the lateral interaction potential, we used the same pragmatic approach as described in literature[6], where it was used for a cobalt system. There it is stated that the lateral interaction is mainly caused by the exponential nature of electronic repulsion of electron density on the adsorbates. Nickel and cobalt are same row neighbors in the periodic table and their electronegativities are almost identical[13] (1.91 and 1.88, respectively), therefore it is expected that the part of electron density on the adsorbates causing the lateral interaction is quite similar for the two transition metals. In order to properly validate and justify the use of the same atom-based lateral interaction penalties, we conducted a series of MKM simulations using different penalty values and analyzed the effect on the apparent activation energy, the reaction orders and the surface coverage. Table S 23 shows the values we used for testing the lateral interaction potential.

Table S 23. Tested penalties for the lateral interaction potential.

Lateral Interaction Penalty			
Carbon	Oxygen	Hydrogen	
90	90	30	
75	75	25	
75	75	20	
70	75	25	
70	70	5	
70	70	0	
65	75	25	
60	60	15	*
45	45	5	
10	10	0	

* Values used in literature[6]

Other settings were kept the same compared to the simulations as presented in the manuscript. Thus, the most stable adsorption energy was used for other reaction intermediates, CO^* was destabilized with 40 kJ/mol and H^* was destabilized with 20 kJ/mol. The simulations were performed with an initial CO_2:H_2 mixture of 1:4, a total pressure of 1 bar and temperatures between 500 - 800 K.

[6] Zijlstra, B.; Broos, R. J. P.; Chen, W.; Filot, I. A. W.; Hensen, E. J. M. First-Principles Based Microkinetic Modeling of Transient Kinetics of CO Hydrogenation on Cobalt Catalysts. *Catal. Today* **2020**, *342* (October 2018), 131–141. https://doi.org/10.1016/j.cattod.2019.03.002.

[13] Science Notes and Projects. https://sciencenotes.org/list-of-electronegativity-values-of-the-elements (accessed September 13, 2022).

J. Sensitivity Analysis on the Lateral Interaction Potential

Apparent activation energy

In Figure S 58, the apparent activation energies (E_{app}) are plotted for each of the four facets with a change in lateral interaction potential. For Ni(211) the apparent activation energy is not effected by the lateral interaction potential at the temperature of interest. For Ni(111) and Ni(110) the differences are subtle and the E_{app} seems to be converged if the lateral interaction > C10-O10-H0. The results for Ni(100) are more spread out into three regions. The lowest E_{app} is obtained when the penalty for hydrogen is very small (H = 0 kJ/mol and H = 5 kJ/mol), which means that compared to C and O containing intermediates relatively much hydrogen is present on the surface (see analysis of the surface coverage at p.79). This means that the hydrogenation of C^* is more facile. The opposite is true when the penalty for hydrogen is very high (H = 25 kJ/mol and H = 30 kJ/mol). The resulting E_{app} is higher because it is more difficult to hydrogenate carbonaceous species. The intermediate region is obtained with a hydrogen penalty of 15 and 20 kJ/mol.

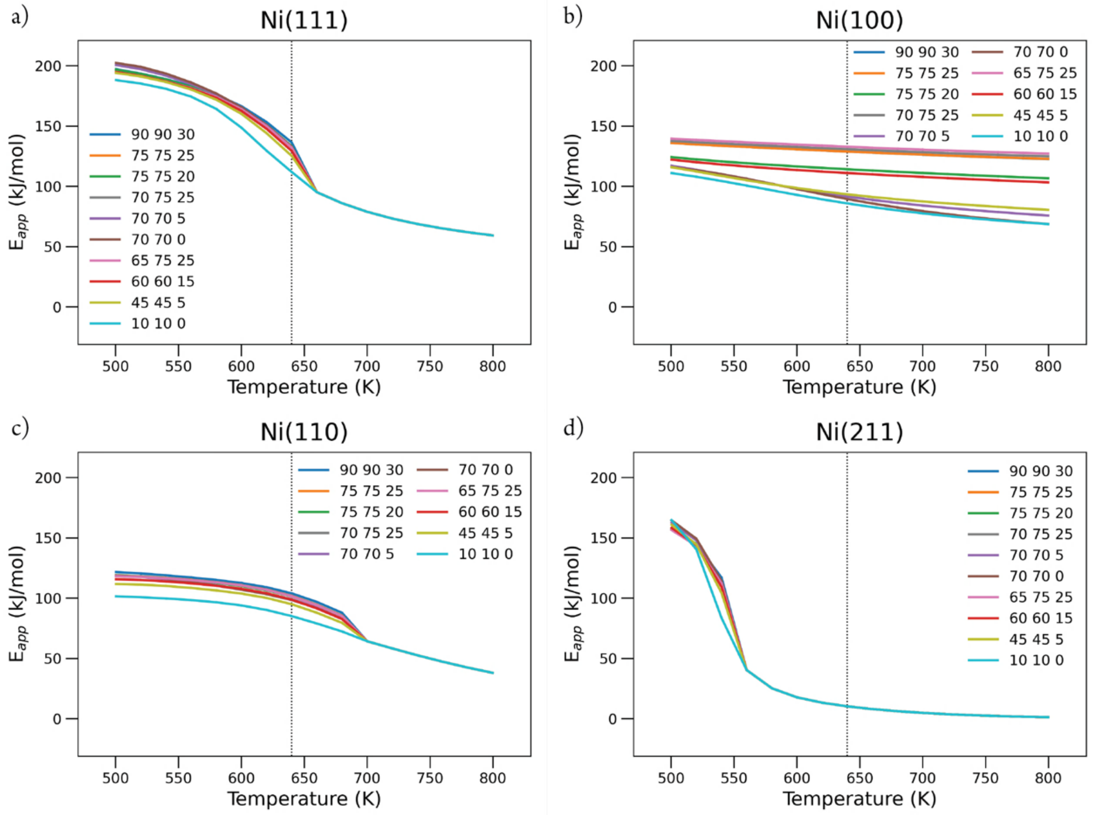

Figure S 58. Apparent activation energy as a function of temperature with different penalties for the lateral interaction potential. In the legend the first, second and third number refer to the lateral interaction penalty for C, O and H, respectively. a) Ni(111), b) Ni(100), c) Ni(110) and d) Ni(211).

Reaction orders

On the next page the reaction orders in CO_2 and H_2 are plotted as a function of temperature (Figure S 59). Note: the scale of the y-axis differs among different facets. The reaction orders in CO_2 and H_2 are not sensitive on the penalty values for the lateral interaction potential for Ni(211). In case of Ni(111) and Ni(110) the orders are well converged at 640 K when the penalties of the potential > C10-O10-H0. For Ni(100) results are again spread in three regions, depending on the hydrogen penalty.

J. Sensitivity Analysis on the Lateral Interaction Potential

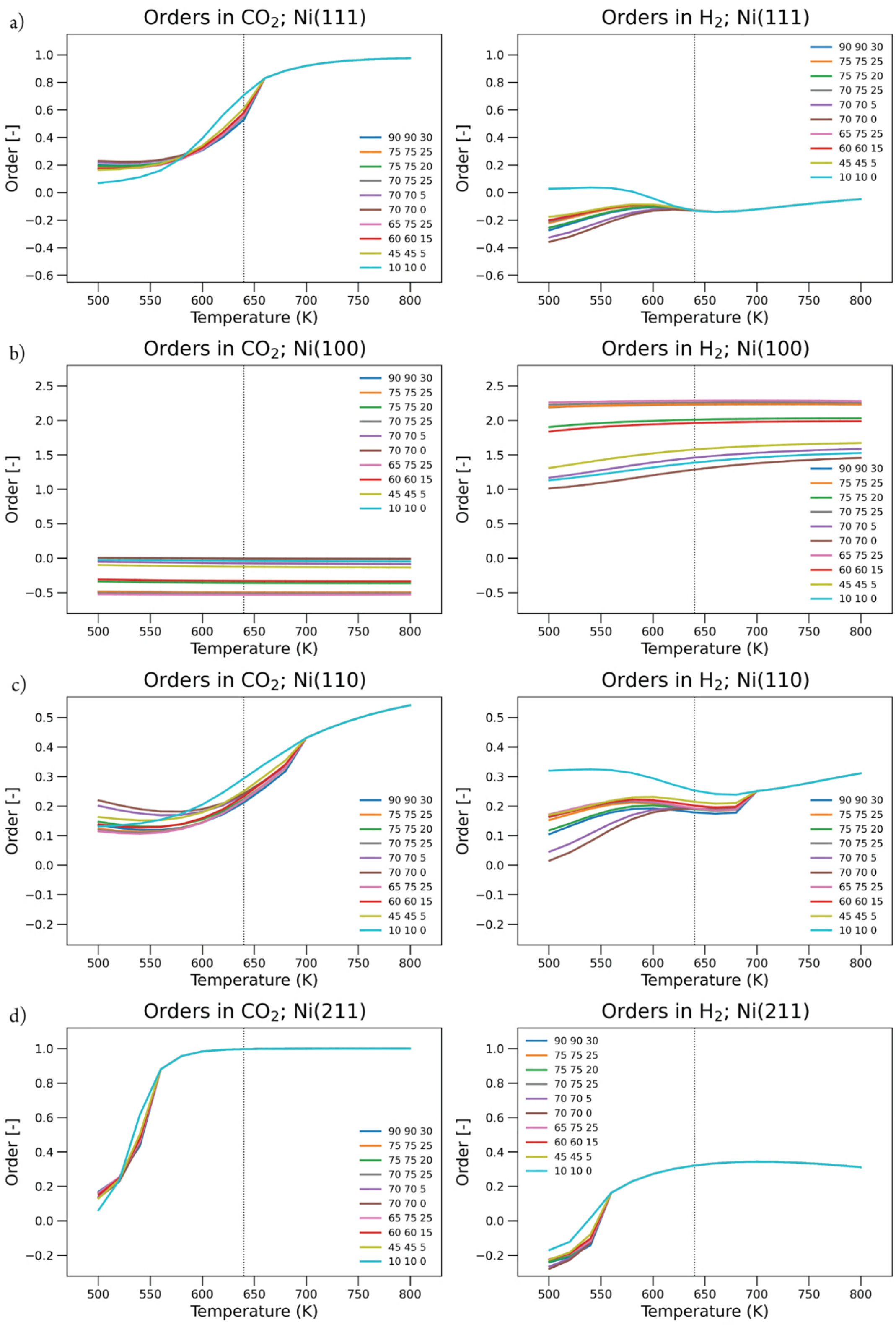

Figure S 59. The reaction orders in CO_2 (left) and H_2 (right) as a function of temperature with different penalties for the lateral interaction potential. In the legend the first, second and third number refer to the lateral interaction penalty for C, O and H, respectively. Note: the scale of the y-axis differs among different facets. a) Ni(111), b) Ni(100), c) Ni(110) and d) Ni(211).

J. Sensitivity Analysis on the Lateral Interaction Potential

Coverage

In Figure S 60, the resulting surface coverage on the terrace sites – Ni(111) and Ni(100) – are presented. For Ni(111) it is apparent that the lateral interaction potential is not affecting the trends in surface coverage at 640 K. However, at lower temperatures it is clear that the penalties should have a minimum value (> C10-O10-H0) in order to prevent unphysically high coverage of the surface. For example, at 500 K the surface is covered 40% with CO* and 50% of H*. In the case of Ni(100) there is a direct effect between the coverage with C* and H* with a changing potential. This relates back to the fact that the most stable reaction intermediate on Ni(100) is carbon. With the lateral interaction potential from literature[13] (C60-O60-H15) the total surface coverage seems to be converged at 75%.

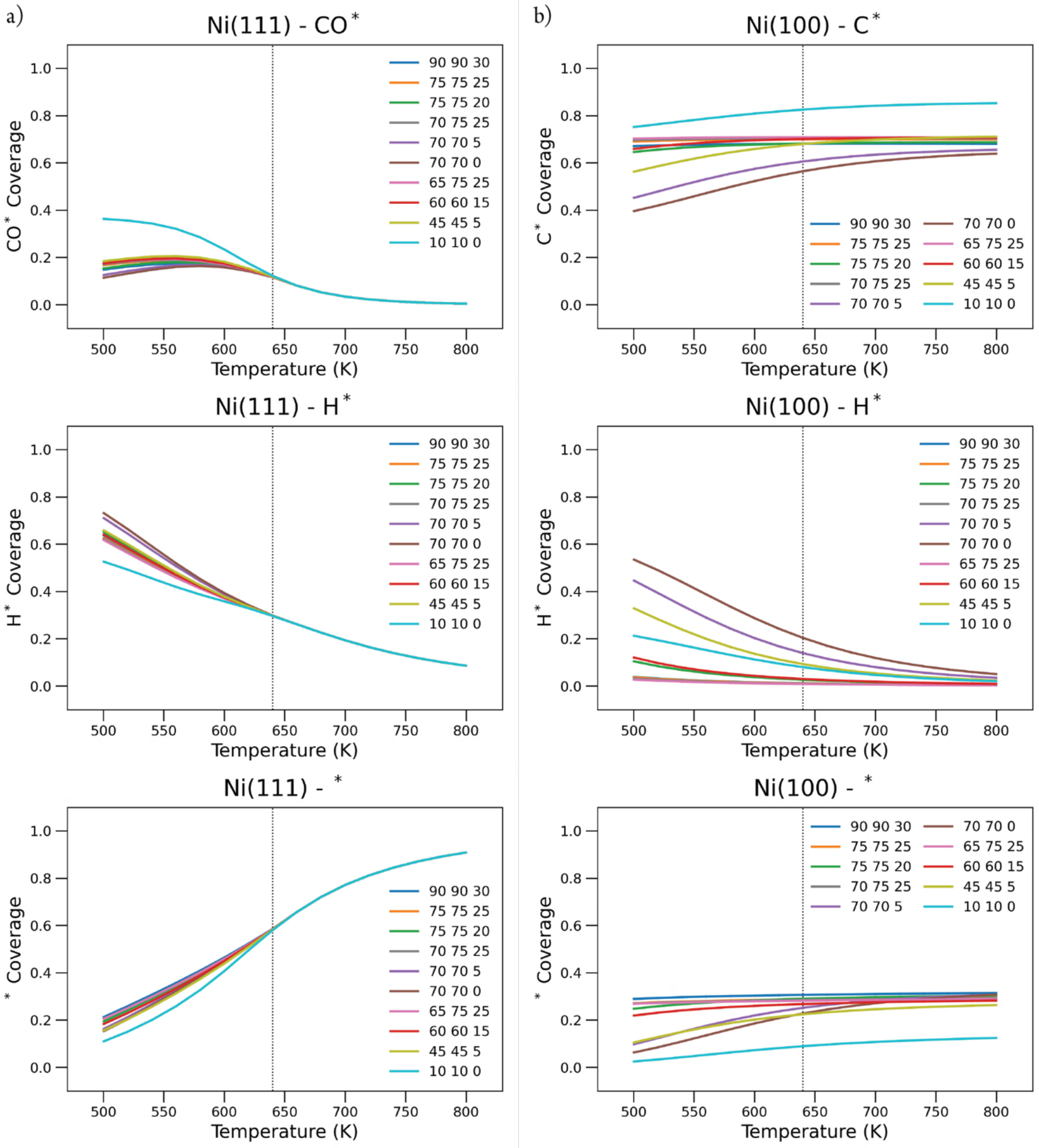

Figure S 60. Surface coverage as a function of temperature with different penalties for the lateral interaction potential. In the legend the first, second and third number refer to the lateral interaction penalty for C, O and H, respectively. a) The surface coverage of CO*, H* and * for Ni(111). b) The surface coverage of C*, H* and * for Ni(100).

J. Sensitivity Analysis on the Lateral Interaction Potential

The coverage plots obtained for the stepped facets Ni(110) and Ni(211) shown in Figure S 61, clearly reveal that the lateral interaction potential does not affect the trends in the surface coverage.

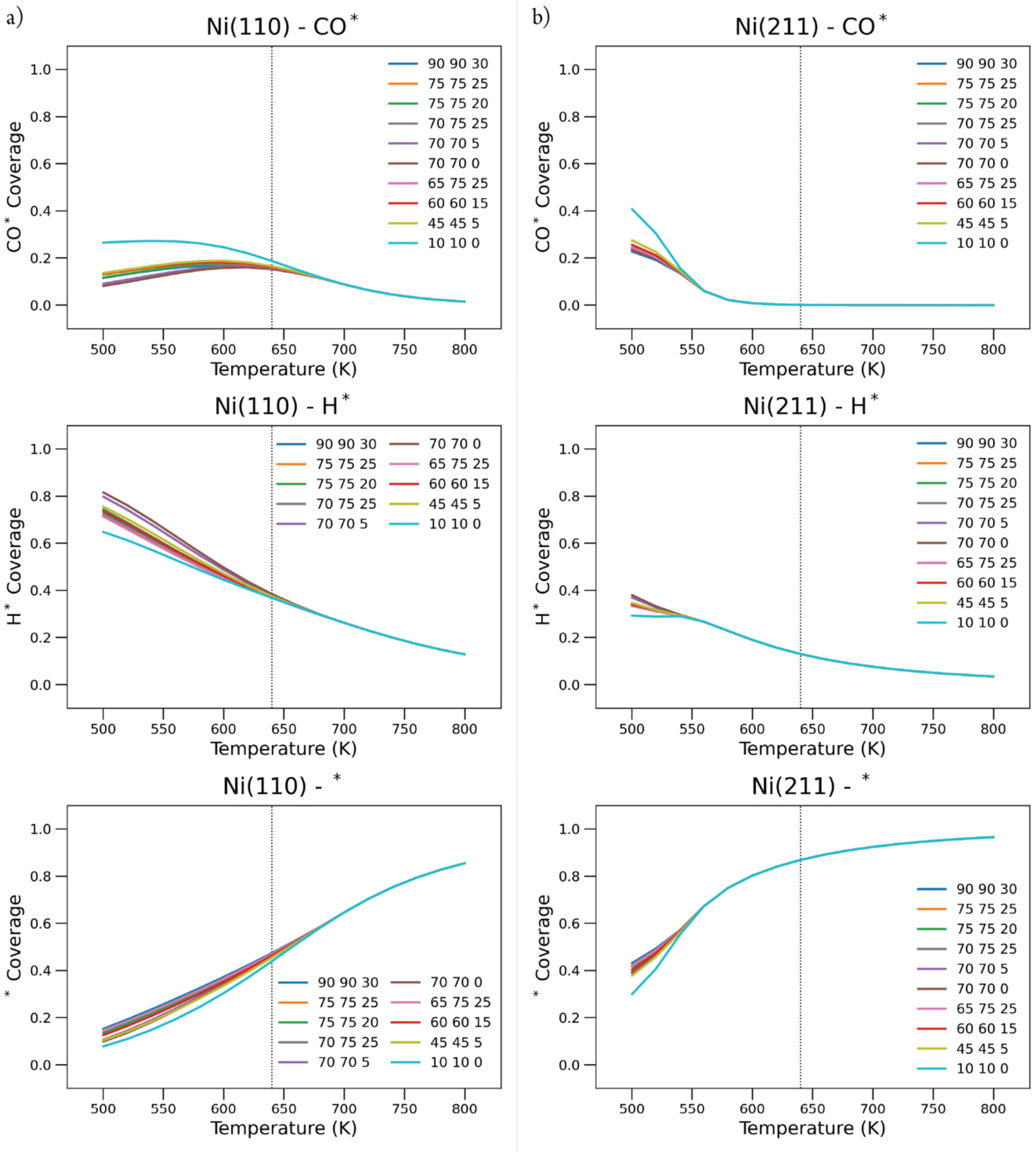

Figure S 61. Surface coverage as a function of temperature with different penalties for the lateral interaction potential. In the legend the first, second and third number refer to the lateral interaction penalty for C, O and H, respectively. The surface coverage of CO^*, H^* and * for a) Ni(110) and b) Ni(211).

Conclusion lateral interaction potential

Based on the results in the E_{app}, reaction orders in H_2 and CO_2 and the surface coverage we have seen that the lateral interaction potential applied on a cobalt system[6] is justified to use in our study on nickel.

K Wulff Constructions

Wulff Constructed Nanoparticles

The surface density of the different types of active sites as a function of the Wulff constructed nanoparticle size is shown in Figure S 62. On the next page in Table S 24, an overview of the Wulff constructions with their size, number of bulk atoms and corresponding partition of each facet is tabulated.

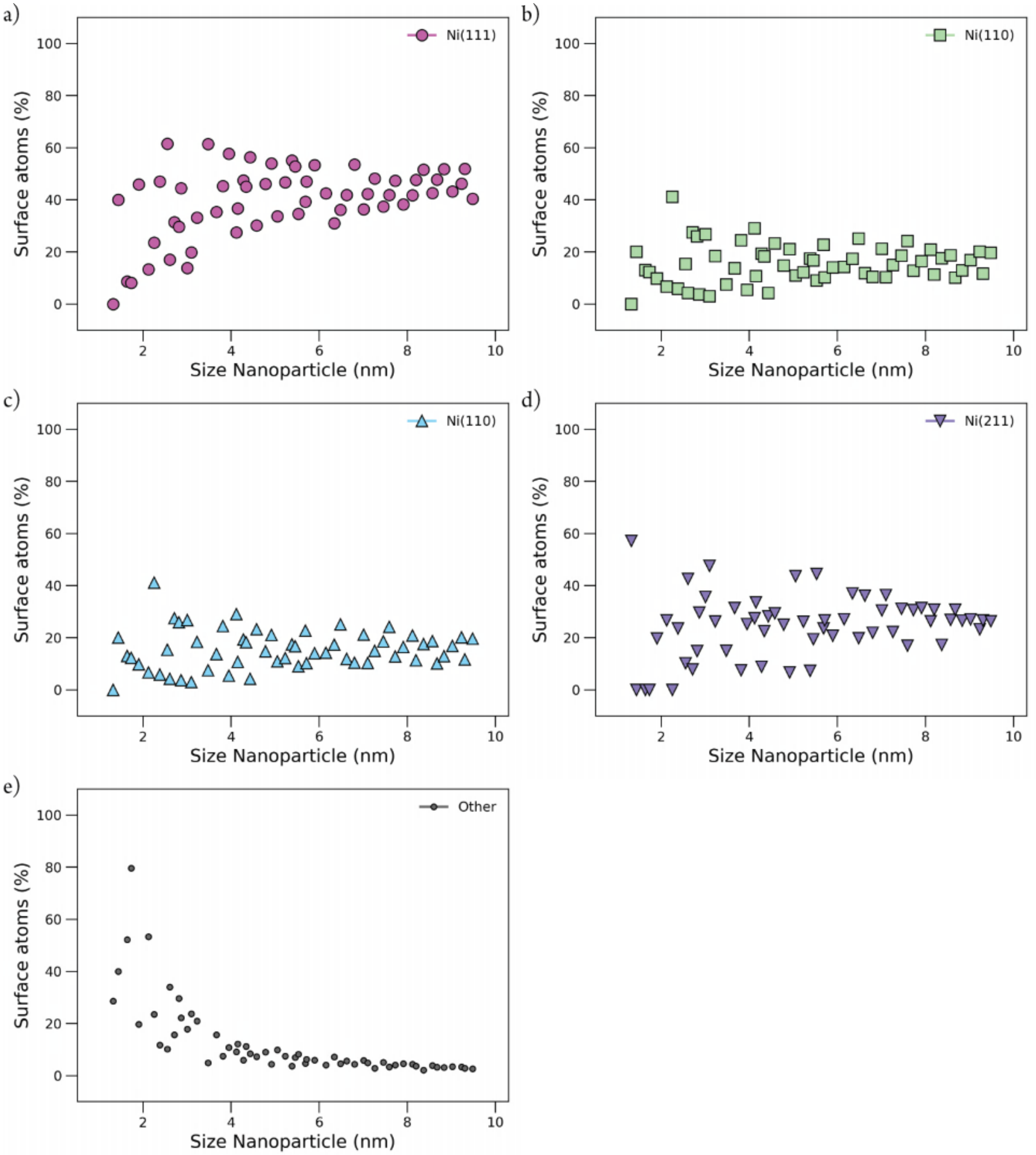

Figure S 62. As a function of the Wulff constructed nanoparticle size the surface density of a) Ni(111), b) Ni(100), c) Ni(110), d) Ni(211) and e) Other atoms.

Table S 24. Overview of Wulff constructed nanoparticles with their size and total number of atoms, number of surface- and bulk-atoms, as well as the corresponding partition of each facet per Wulff constructed nanoparticle.

Wulff Constructed Nanoparticles								
	# Atoms			# Atoms assigned				
**Size (nm)**	**Total**	**Surface**	**Bulk**	**Ni(111)**	**Ni(100)**	**Ni(110)**	**Ni(211)**	**Other**
1.03	19	18	1	0	0	12	0	6
1.19	43	42	1	0	6	12	0	24
1.32	55	42	13	0	6	0	24	12
1.44	79	60	19	24	0	12	0	24
1.64	135	92	43	8	24	12	0	48
1.73	141	98	43	8	0	12	0	78
1.90	201	122	79	56	6	12	24	24
2.12	321	180	141	24	0	12	48	96
2.26	369	204	165	48	24	84	0	48
2.38	405	204	201	96	24	12	48	24
2.55	459	234	225	144	6	36	24	24
2.61	603	282	321	48	6	12	120	96
2.71	675	306	369	96	54	84	24	48
2.82	699	324	375	96	0	84	48	96
2.87	735	324	411	144	0	12	96	72
3.01	959	404	555	56	24	108	144	72
3.10	1007	404	603	80	24	12	192	96
3.23	1157	458	699	152	6	84	120	96
3.48	1289	482	807	296	54	36	72	24
3.67	1769	612	1157	216	24	84	192	96
3.81	1865	636	1229	288	96	156	48	48
3.95	1979	666	1313	384	6	36	168	72
4.12	2459	786	1673	216	54	228	216	72
4.15	2555	786	1769	288	54	84	264	96
4.28	2675	810	1865	384	150	156	72	48
4.34	2771	852	1919	384	24	156	192	96
4.43	2831	852	1979	480	24	36	240	72
4.58	3439	980	2459	296	96	228	288	72
4.78	3829	1058	2771	488	54	156	264	96
4.92	3949	1082	2867	584	150	228	72	48
5.05	4585	1212	3373	408	24	132	528	120
5.23	5113	1284	3829	600	96	156	336	96
5.38	5257	1308	3949	720	216	228	96	48
5.45	5407	1362	4045	720	54	228	264	96
5.53	6043	1458	4585	504	54	132	648	120
5.69	6499	1530	4969	600	150	348	360	72
5.71	6643	1530	5113	720	150	156	408	96
5.89	7027	1620	5407	864	96	228	336	96
6.15	8127	1772	6355	752	216	252	480	72
6.34	9621	2010	7611	624	150	348	744	144
6.49	9957	2058	7899	744	294	516	408	96
6.63	10401	2124	8277	888	96	252	768	120
6.80	11121	2196	8925	1176	216	228	480	96
7.01	12615	2442	10173	888	150	516	744	144
7.10	12843	2442	10401	1032	150	252	888	120
7.26	13251	2490	10761	1200	294	372	552	72
7.46	15395	2780	12615	1040	216	516	864	144
7.59	15779	2828	12951	1184	384	684	480	96
7.73	16373	2906	13467	1376	150	372	888	120
7.91	18533	3138	15395	1200	294	516	984	144
8.12	19349	3276	16073	1368	216	684	864	144
8.20	19649	3276	16373	1560	216	372	1008	120
8.37	20321	3348	16973	1728	384	588	576	72
8.57	23015	3666	19349	1560	294	684	984	144
8.67	23315	3666	19649	1752	294	372	1128	120
8.84	24203	3804	20399	1968	216	492	1008	120
9.02	27091	4076	23015	1760	384	684	1104	144
9.23	28057	4226	23831	1952	294	852	984	144
9.30	28429	4226	24203	2192	294	492	1128	120
9.48	30637	4458	26179	1800	486	876	1176	120

Turnover Frequency

In Figure S 63a-d, the contribution of each nickel facet to the turnover frequency (TOF) of the Wulff constructed nanoparticles is plotted. The summation of these TOFs is shown in Figure S 63e.

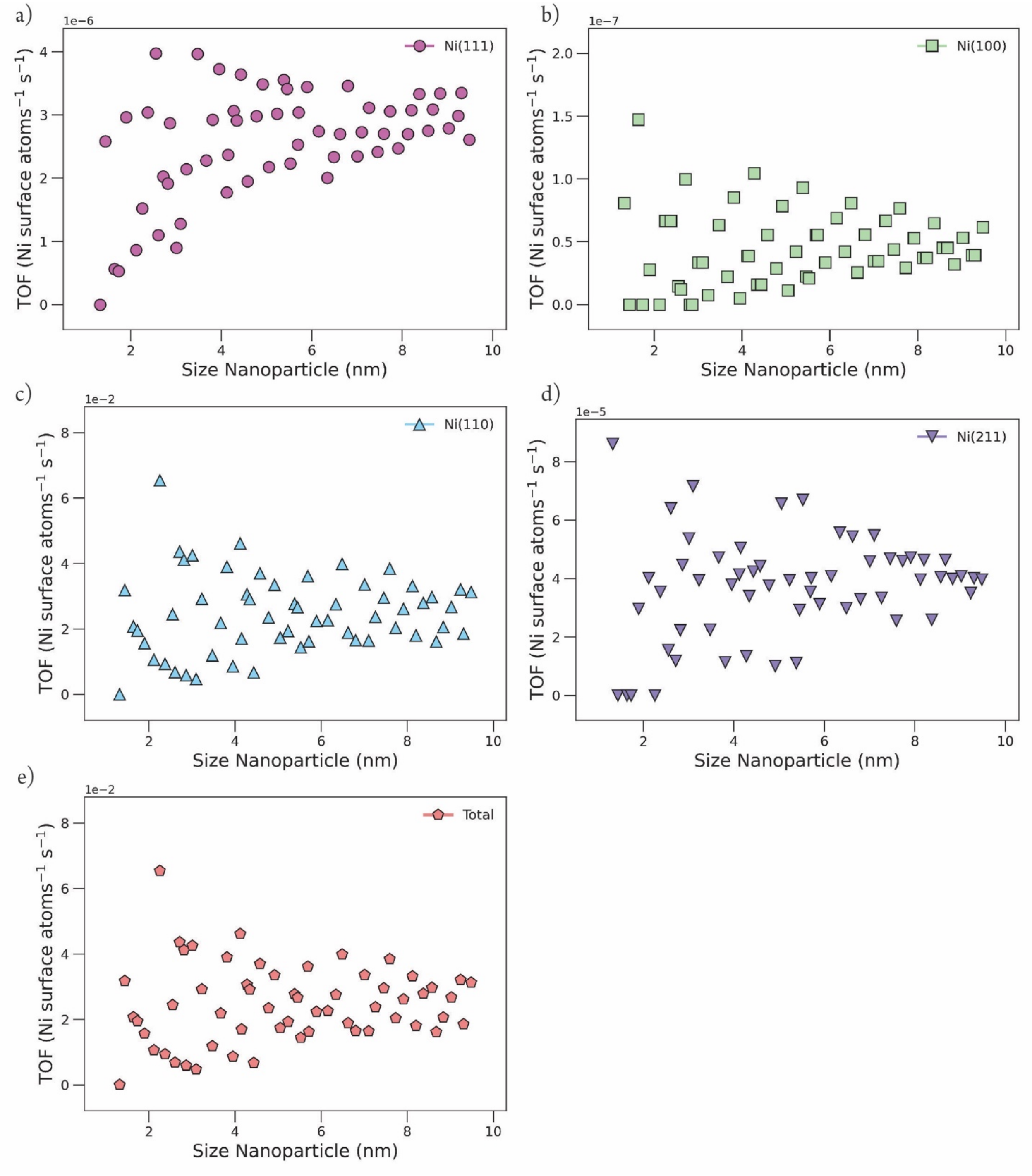

Figure S 63. As a function of the size of the Wulff constructed nanoparticles, the contribution of each nickel facets to the turnover frequency (TOF) of the Wulff constructions is plotted for a) Ni(111), b) Ni(100), c) Ni(110), d) Ni(211). The summation of these TOFs is plotted in e). Note: the scale of the y-axis differs among different facets.

In Figure S 64 we show how an overall trend is obtained from the TOF plots. In Figure S 64a the total TOF (same data as depicted in Figure S 63e on the previous page) is represented with a continuous line. In Figure S 64b we calculated the moving average using a window of 2 after which a polynomial with order 10 was applied in Figure S 64c.

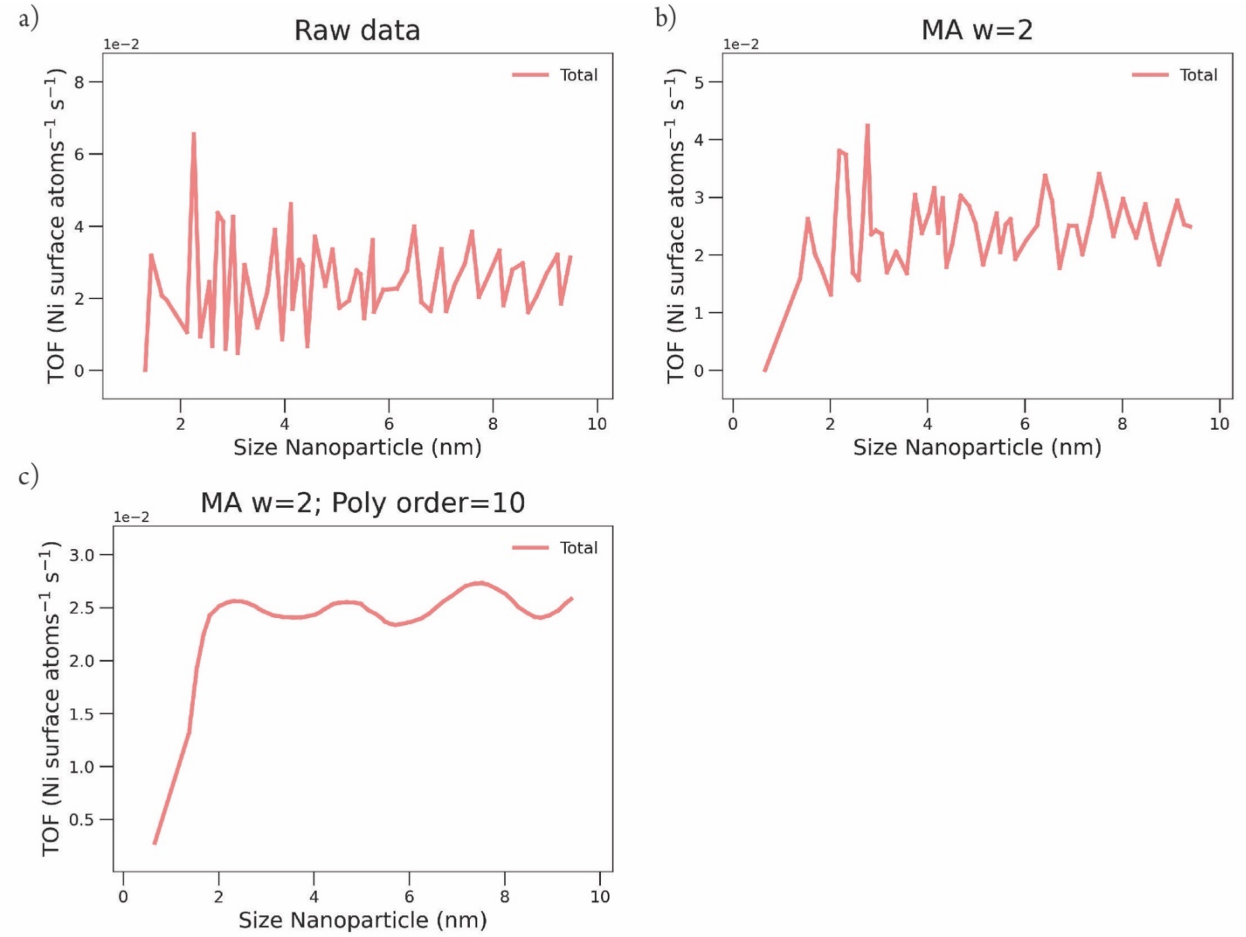

Figure S 64. The turnover frequency (TOF) of the Wulff constructed nickel nanoparticles as a function of its size is shown in a), obtained by summation of the TOFs for Ni(111), Ni(100), Ni(110) and Ni(211). A moving average with window 2 was taken for the TOF and plotted in b). The plot shown in c) represents the total TOF, with moving average of window 2 and smoothened using a polynomial order 10.

The shape of the smoothened TOF plot (Figure S 64c) is not yet comparable to the TOF plot obtained from the catalytic CO_2 hydrogenation over Ni/SiO_2 catalysts, as shown in Fig.1a of the manuscript. Therefore, it can be assumed that the under coordinated atoms do have some activity.

The activity of the undercoordinated atoms was estimated by a first order approximation. Therefore, the under coordinated atoms were assigned with zero activity, with the activity of Ni(211) atoms and with 0.01, 0.1, 0.5 and 1 times the activity of Ni(110) atoms. The resulting TOF plots are depicted in Figure S 65. From this figure it is clear that the characteristic optimum at 2 nm observed in experiments becomes clearly visible when the undercoordinated atoms are assigned with 0.5 or 1 times the activity of Ni(110) atoms.

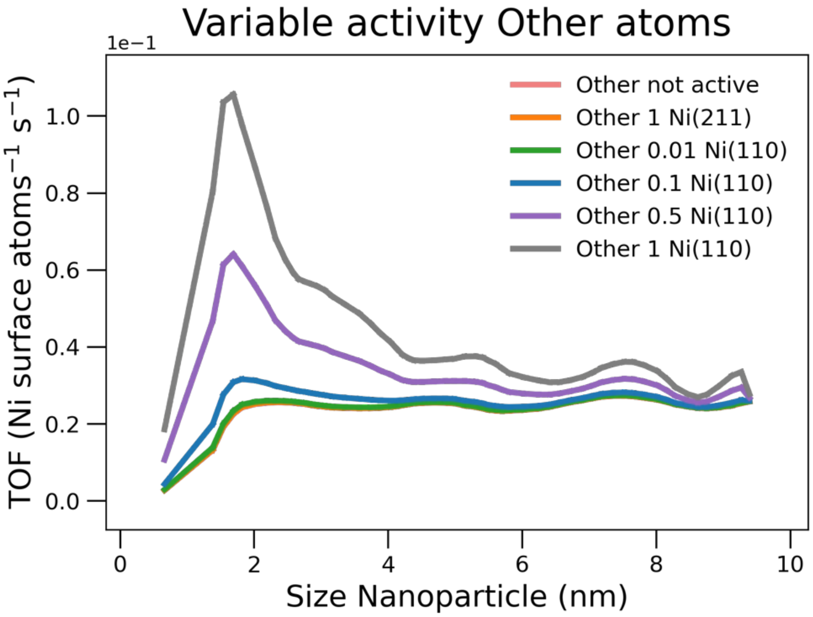

Figure S 65. The turnover frequency (TOF) of the Wulff constructed nickel nanoparticles as a function of its size, obtained by summation of the TOFs of Ni(111), Ni(100), Ni(110) and Ni(211), with an increasing activity for the Other atoms.
